# Proteome-level assessment of origin, prevalence and function of leucine-aspartic acid (LD) motifs

**DOI:** 10.1093/bioinformatics/btz703

**Published:** 2019-10-04

**Authors:** Tanvir Alam, Meshari Alazmi, Rayan Naser, Franceline Huser, Afaque A Momin, Veronica Astro, SeungBeom Hong, Katarzyna W Walkiewicz, Christian G Canlas, Raphaël Huser, Amal J Ali, Jasmeen Merzaban, Antonio Adamo, Mariusz Jaremko, Łukasz Jaremko, Vladimir B Bajic, Xin Gao, Stefan T Arold

**Affiliations:** 1 Computational Bioscience Research Center (CBRC), Division of Computer, Electrical and Mathematical Sciences & Engineering (CEMSE), Saudi Arabia; 2 Computational Bioscience Research Center (CBRC), Division of Biological and Environmental Sciences and Engineering (BESE), Saudi Arabia; 3 Division of Biological and Environmental Sciences and Engineering (BESE), Saudi Arabia; 4 Core Labs, Thuwal, Saudi Arabia; 5 Division of Computer, Electrical and Mathematical Sciences & Engineering (CEMSE), King Abdullah University of Science and Technology (KAUST), Thuwal 23955-6900, Saudi Arabia

## Abstract

**Motivation:**

Leucine-aspartic acid (LD) motifs are short linear interaction motifs (SLiMs) that link paxillin family proteins to factors controlling cell adhesion, motility and survival. The existence and importance of LD motifs beyond the paxillin family is poorly understood.

**Results:**

To enable a proteome-wide assessment of LD motifs, we developed an active learning based framework (*LD m*otif *f*inder; *LDMF*) that iteratively integrates computational predictions with experimental validation. Our analysis of the human proteome revealed a dozen new proteins containing LD motifs. We found that LD motif signalling evolved in unicellular eukaryotes more than 800 Myr ago, with paxillin and vinculin as core constituents, and nuclear export signal as a likely source of *de novo* LD motifs. We show that LD motif proteins form a functionally homogenous group, all being involved in cell morphogenesis and adhesion. This functional focus is recapitulated in cells by GFP-fused LD motifs, suggesting that it is intrinsic to the LD motif sequence, possibly through their effect on binding partners. Our approach elucidated the origin and dynamic adaptations of an ancestral SLiM, and can serve as a guide for the identification of other SLiMs for which only few representatives are known.

**Availability and implementation:**

*LDMF* is freely available online at www.cbrc.kaust.edu.sa/ldmf; Source code is available at https://github.com/tanviralambd/LD/.

**Supplementary information:**

Supplementary data are available at *Bioinformatics* online.

## 1 Introduction

Cellular signal transduction networks rely on the recognition of short linear motifs (SLiMs) by their cognate ligand binding domains (Gould *et al.*, 2010). These motifs are contained on a single contiguous amino acid stretch of typically <15 residues, and do not require to be embedded in a 3D protein framework to be functional. The binding energy of many SLiMs is dominated by only a few residues, resulting in moderate to low binding affinities [with dissociation constants (*K_d_*’s) of 1–150 µM] that are ideal for mediating transient signalling interactions (Diella *et al.*, 2008). On the one hand, this characteristic facilitates emergence and diversification of SLiMs, and hence the restructuration and evolutionary adaptation of an organism’s interactome (Ren *et al.*, 2008). On the other hand, the resulting sequence motif degeneration and binding promiscuity hamper our capacity to computationally identify SLiMs and their biologically relevant binding partners (Edwards and Palopoli, 2015). This difficulty severely limits our capacity to evaluate the spread, adaptation and possibly origin of SLiMs.

**Fig. 1.**
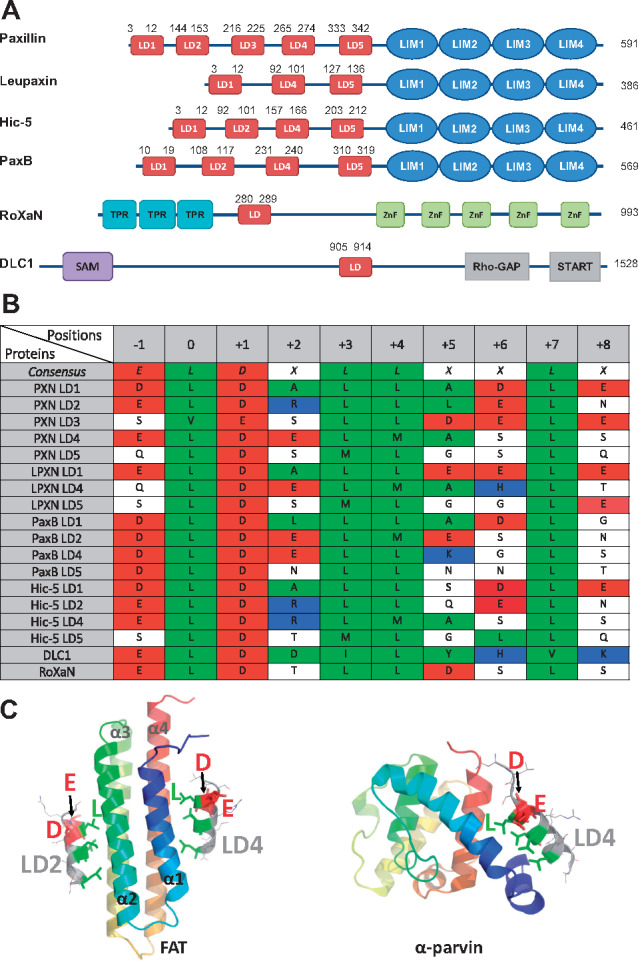
Overview of LD motifs. (**A**) Schematic representation of human paxillin family members (paxillin, leupaxin and Hic-5) and PaxB from *Dictyostelium discoideum*. TPR, tetratricopeptide repeat; Znf, zinc-finger; SAM, sterile *α*-motif; START, STAR-related lipid transfer domain. (**B**) Sequence alignment of selected known LD motifs. Sequence positions are numbered with respect to the first leucine of the LD motif (numbered 0). Acidic (red), basic (blue) and hydrophobic (green) residues are highlighted. PXN, paxillin. LPXN, leupaxin. All sequences are from human proteins, except for PaxB (*D. discoideum*). (**C**) Structure of LD motifs bound to FAK FAT and α-parvin. Ribbon diagrams of FAT and α-parvin are colour-ramped from blue (N-terminus) to red (C-terminus). LD motifs are shown in grey, with key residues shown as stick models in green (hydrophobic) or red (acidic). Position −1 (D), 0 (L) and +1 (E) are labelled

LD motifs were first described in 1996 as novel SLiMs that associate paxillin with the cell adhesion proteins vinculin and the focal adhesion kinase (FAK) (Brown *et al.* 1996, 1998). Paxillin and its family members leupaxin and Hic-5 (also called TGFB1I1 or ARA55) contain in their N-terminal region four or five of these motifs, named after the first two amino acids of their sequence consensus LDXLLXXL (Alam *et al.*, 2014; Brown *et al.*, 1998) (Fig. 1A and B). The N-terminal LD motifs, together with other protein–protein interaction sites located in this region, orchestrate the dynamic assembly of different signalling complexes (Deakin and Turner, 2008). The C-terminal region of paxillin family proteins, which contains four double zinc-finger *l*in-11, *i*sl-1, *m*ec-3 (LIM) domains, mediates recruitment to integrin clusters at sites of cellular adhesion, but also nuclear localization and nuclear receptor interactions (reviewed by Alam *et al.*, 2014). Using these protein interaction motifs, paxillin family proteins establish a communication between cell adhesions and the nucleus, functionally linking gene expression with cell attachment (Ma and Hammes, 2018). Thus, paxillin family members play important roles in embryonic development, epithelial morphogenesis and the immune response (Lopez-Colome *et al.*, 2017). As mediators of cellular motility and survival, paxillin family proteins are also key factors governing associated pathological conditions, such as inflammation, cardiovascular disease and the development, spread and metastasis of tumours (Lopez-Colome *et al.*, 2017). Moreover, LD motifs play a role in infectious diseases, because paxillin LD motifs are targets of a subset of human papilloma viruses, which cause cervical cancers (Vande Pol and Klingelhutz, 2013), and the *Chlamydia* virulence factor TarP uses a sequence mimicking paxillin LD motifs for remodelling actin to facilitate bacterial invasion (Thwaites *et al.*, 2014).

More than a dozen proteins were shown to interact with paxillin family LD motifs, using LD motif binding domains (LDBDs) of at least six different domain architectures (reviewed by Alam *et al.*, 2014). Most of these proteins are important players in membrane-proximal intracellular structures that connect cell-surface receptors with downstream signalling pathways and/or the cytoskeleton, mostly in focal adhesions (FAs) or similar structures (FAK, PYK2, vinculin, talin, GIT, parvin, Bcl-2) (Maziveyi and Alahari, 2017). Other proteins are involved in the transport of mRNA (PABP1) for mRNA delivery to sites of cellular adhesion, or in nuclear export (XPO1, also called CRM1) (Harb *et al.*, 2008; Woods *et al.*, 2005); indeed, some LD motifs of paxillin, leupaxin or Hic-5 were reported to also function as a nuclear export signal (NES) (Alam *et al.*, 2014). In all cases where experimental 3D structures are available, LD motifs form amphipathic helices that use the hydrophobic side chains (LDXLLXXL) of the motif to dock onto an elongated hydrophobic patch on LDBDs, while the negative charge (LDXLLXXL) forms ionic interactions with basic charges next to the hydrophobic patch (Fig. 1C) (Alam *et al.*, 2014; Zacharchenko *et al.*, 2016).

The biological importance of LD motifs, and the discovery of more than a dozen LD motif–interacting proteins (many of which have evolved different LDBD folds) (Alam *et al.*, 2014), motivated efforts to discover LD motifs outside the paxillin family. However, to date, motifs of the LDXLLXXL consensus were only experimentally confirmed in two human proteins: the deleted in liver cancer 1 (DLC1) tumour suppressor gene (Li *et al.*, 2011; Zacharchenko *et al.*, 2016), and the rotavirus ‘X’-associated non-structural protein (RoXaN) (Harb *et al.*, 2008). In addition to these, gelsolin (an actin binding, severing and capping protein mediating osteoclastic actin cytoskeletal organization) was found to bind the LDBD of PYK2 through a similar motif (LDXALXXL) (Wang and Dunbrack, 2003), whereas the FAK LDBD also binds to non-LD motif sequences, such as the CD4 endocytosis motif (Garron *et al.*, 2008) or the DCC-P3 motif (Xu *et al.*, 2018).

Herein we combined computational, biophysical and structural methods to produce a machine-learning tool (called *LD m*otif *f*inder; *LDMF*) for the automated detection of canonical LD motifs in proteomes. Combined with experimental validation we used *LDMF* to assess the origin, prevalence and function of LD motifs across species.

## 2 Materials and methods

### 2.1 Overview on computational method development

**Fig. 2.**
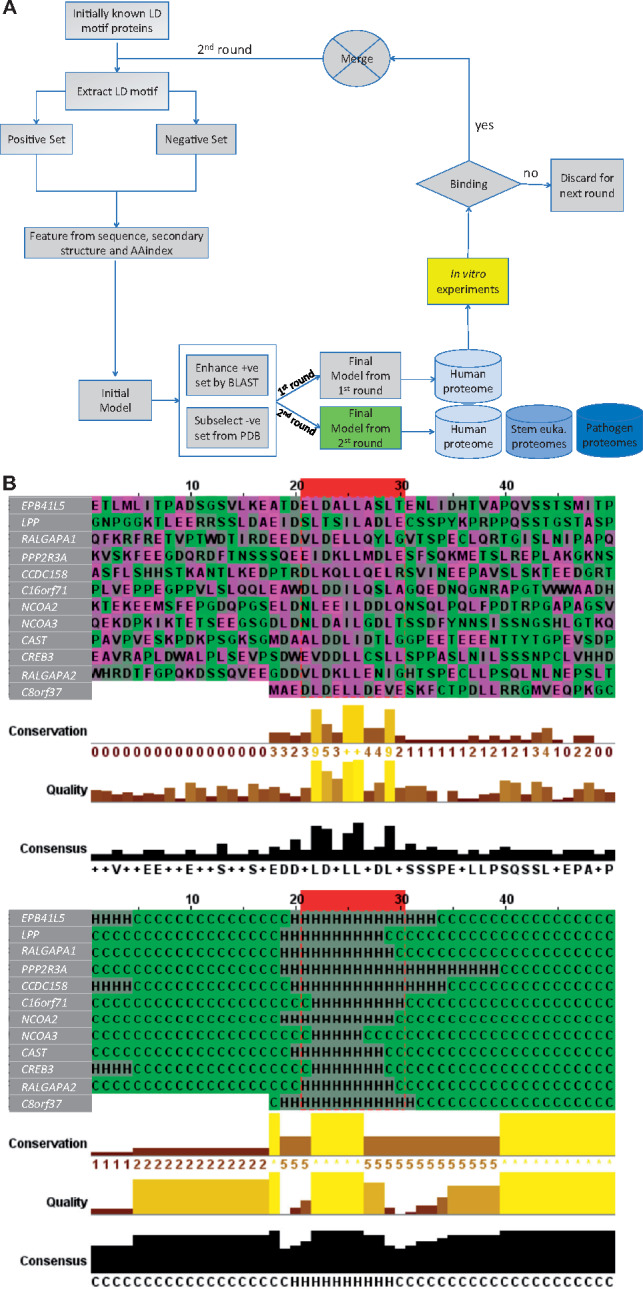
Flowchart of the *LDMF* tool, and features of the predicted LD motif sequences. (**A**) Our learning process contains three iterations. The first iteration was by training a support vector machine (SVM) model based on the 18 known LD motifs as the positive set and randomly drawn sequences as the negative one. Sequence, secondary structure and AAindex features of these sets were used to build an initial model. This model was expected to have poor prediction performance because the randomly drawn negative sequences are expected to be easily differentiable from the positive ones. We then applied this initial model to identify putative LD motifs in close orthologs of our six positive-set proteins, using standard protein–protein unidirectional BLAST (blastp) (Altschul *et al.*, 1997) (see Supplementary Material for details). This step resulted in additional 40 LD motif sequences that we manually checked and added to the positive set. The initial model was then applied to the protein data bank (PDB) to find sequences that satisfy some of the key features, but not all of them. These sequences are similar to the true motifs in some aspects and thus provide a much more difficult negative set for the second iteration of training. These training sets were used to build the ‘final’ first round model, with which we scanned the human proteome (20 159 sequences). All predicted novel LD motifs were synthesized as peptides and used in *in vitro* binding experiments. Those sequences that showed binding were included in the positive set of the final iteration of training. The final model of the second round was used to predict LD motifs in various proteomes. (**B**) The ten amino acids constituting the LD motif core are highlighted inside the red box. The twenty up- and down-stream residues of the flanking regions are shown. *Top:* amino acid sequences. *Bottom:* secondary structure. This figure was generated by Jalview (Waterhouse *et al.*, 2009)

To provide a high-accuracy algorithm for proteome-scale detection of LD motifs, we used a machine-learning approach that combines secondary structure (SS) prediction and physiochemical properties (from the AAindex database) of amino acids of the LD motif region. We formalized this problem as a binary-class classification problem of 10-mers, i.e. a subsequence of 10 amino acids, where LD motifs are considered as the positive set and 10-mers that are not LD motifs are considered as the negative set, as described in Figure 2A. Using the set of amino acid sequences from bona fide LD motifs and their surrounding regions, we built several position weight matrices (PWMs). These PWMs were used to scan proteins for generating scores which were used as feature set for the machine-learning model. We also used predicted SS of bona fide LD motifs and their surrounding regions to construct a set of PWMs. We generated additional features from the bona fide LD motif regions using the physiochemical properties (e.g. hydrophobicity, volume, electric charge) of amino acids. The combination of the features from sequence, SS and physiochemical properties was used to build our machine-learning model to predict LD motifs. For details, please see Supplementary Material.

### 2.2 Bioinformatics

Publicly available web servers were used for prediction of protein disorder, secondary/tertiary structure, transmembrane helices and NES, as described in Supplementary Material.

### 2.3 Proteins and peptides

Human α-parvin-CH_C_ (residues 242–372), the FAT domain of human FAK (892–1052) and the rat GIT1 (647–770) were expressed as GST-fusion proteins in *Escherichia* *coli* BL21 as described previously (Arold *et al.*, 2002; Lorenz *et al.*, 2008; Schmalzigaug *et al.*, 2007) (see Supplementary Material).

Peptides were purchased from GenScript with and without FITC-Ahx N-terminal modification, with the following sequences: LD4 (SASSATRELDELMASLSD), LD2 (NLSELDRLLLELNAVQ), IBP2 (TPTQQELDQVLERISTMR), RGPA2 (GDDVLDKLLENIGHT), CH037 (AEDLDELLDEVESKFATPD), ICAL (DAALDDLIDTLGGP), FIP1 (SAGEVERLVSELSGGT), WHAMM (PGSMDEVLASLRHG), LPP (AEIDSLTSILADLESS), RGPA1 (EDVLDELLQYLGVT), CP071 (EAWDLDDILQSLQGQ), NCOA2 (SELDNLEEILDDLQNSQ), E41L5 (ATDELDALLASLTENLID), PCP2 (PTPEMDSLMDMLASTQ), NCOA3 (GDLDNLDAILGDLTSSD), RHGO7 (DIFPELDDILYHVKGMQ), PPP2R3A (SQEEIDKLLMDLESFSQ), CREB3 (SDWEVDDLLCSLLSPPA), CCDC158 (DPTRDLKQLLQELRSVIN), Scramble (LSDAMETSSLRDALE).

### 2.3 Biophysical binding assays

Differential Scanning Fluorimetry, Direct anisotropy assay, Anisotropy competition assay, Isothermal Titration Calorimetry and Microscale Thermophoresis were carried out using standard procedures at 25°C, in 20 mM HEPES pH 7.5, 150 mM NaCl, 2 mM EDTA, 1 mM TCEP, unless stated otherwise (see Supplementary Material).

### 2.4 Nuclear magnetic resonance

The ^1^H-^15^N HSQC titration experiments were performed at a temperature of 25°C using a Bruker Avance III 950 MHz NMR spectrometer equipped with a triple resonance inverse TCI CryoProbe (see Supplementary Material).

### 2.5 Data-driven molecular docking

The data-driven HADDOCK 2.1 protocol (van Zundert *et al.*, 2016) was used based on the crystal structures of FAT (1ow8 and 1ow7) to generate the models of complexes for FAT: CCDC158 and FAT: LPP. The NMR chemical shift perturbation (CSP) data were used to define the residues potentially involved in binding. Structures were analysed using PyMol (pymol.org) (see Supplementary Material).

### 2.6 Cellular analyses

eGFP–LD fusion constructs contained an N-terminal eGFP followed by a HRV3C protease recognition site (LEVLFQGP) and then four times the same LD motif sequence, separated by glycine–serine–threonine linkers of different lengths to enable multivalent associations with LDBDs. HeLa cells were cultured in Dulbecco’s modified Eagle’s medium with 10% FBS and transfected with plasmid DNA using Lipofectamine 3000. Cell spreading, immuno-localization experiments, live cell imaging were performed under standard conditions, and as previously described (Astro *et al.*, 2011) (see Supplementary Material).

## 3 Results

### 3.1 Development of a computational LD motif identification method

In 1998, Brown and colleagues used the degenerate sequence pattern (L, V)(D, E)X(L, M)(L, M)XXL to search sequence databases for LD motifs. They found this sequence pattern in a diverse array of proteins, suggesting that LD motifs are relatively abundant (Brown *et al.*, 1998). However, this pattern search currently retrieves >6000 sequences in the human proteome, demonstrating that it results in too many false positives. We also attempted to identify LD motifs in humans using existing SLiM finding tools [e.g. SlimSearch4 (http://slim.ucd.ie/slimsearch/), PSSMSearch (http://slim.ucd.ie/pssmsearch/), FIMO (http://meme-suite.org/tools/fimo)] (Grant *et al.*, 2011; Krystkowiak and Davey, 2017; Krystkowiak *et al.*, 2018). However, these tools failed to predict all known LD motifs and/or predicted an excessive amount of hits, suggestive of too many false positives (Supplementary Table S1). Indeed, whereas bona fide LD motifs are computationally characterized as short α-helical segments within disordered protein regions (see Supplementary Material and Supplementary Fig. S1), the LD motif sequence pattern also appears frequently within the folded core of proteins, where it has a structural role rather than functioning as an SLiM (Supplementary Fig. S2).

To achieve accurate computational LD motif prediction, we decided to build a specific tool (see Materials and methods and Supplementary Material). Given the available data and intended analyses, we chose to focus on the canonical LD motif consensus, excluding ligands that bind LDBDs through a different motif (e.g. CD4). For this, we extended the 8-residue LD motif (LDXLLXXL) by one amino acid on each side into a 10-residue core motif (X^−1^L^0^DXLLXXLX^+9^; the first leucine is numbered as 0), because structural analysis shows that positions −1 and +9 can contact the LDBD surface, and hence may contain LD motif-specific information (Alam *et al.*, 2014; Zacharchenko *et al.*, 2016). As a computable proxy for the (generally unknown) 3D structural context of candidate sequences, we used SS predictions of the core sequence and of the 20 upstream and 20 downstream residues. Machine-learning further included the amino acid sequence for the 10-residue core and 20-residue flanking sequences and the Amino Acid Index (AAindex) to extract volume, hydrophobicity and electric charge for the 10-residue core.

An additional difficulty for machine-learning was the imbalance and definition of the positive and negative datasets. The initial positive set contained only the 18 experimentally confirmed LD motifs from 6 proteins: 4 or 5 from each paxillin family protein (human paxillin, leupaxin and Hic-5 and dictyostelium paxillin-B); 1 from DLC1 (we used isoform 2 which is the ‘canonical sequence’ in UniProt) and 1 from RoXaN (Fig. 1A and B). The negative dataset (i.e. all UNIPROT 10-mer sequences that are not LD motifs) is million-times larger than the positive set, yet undefined because the occurrence of LD motifs is unknown. We solved this problem through an iterative active learning approach (Fig. 2A, Supplementary Material). In each iteration, the model was statistically validated by leave-one-out cross-validation on the positive set, which is known to provide the closest estimation to the true generalization power of a machine-learning model. Support vector machine (SVM) was chosen as the classifier due to two reasons: (i) our positive set was limited, on which more sophisticated machine-learning models, such as deep learning, are known to perform poorly; and (ii) SVM only depends on the support vectors (i.e. the samples on the decision margin) and is thus insensitive to the underlying distribution of the samples, which is difficult to be captured for a small sample size. The resulting final *LDMF* tool identifies LD motifs within our test set (using a leave-one-out cross-validation approach) with high sensitivity (88.88%) and accuracy (99.97%; Fig. 2B, Supplementary Table S2). Given that our machine-learning model was trained on imbalanced data with a much larger negative set, the ‘accuracy’ was expected to be high. Hence, the sensitivity should be considered as the appropriate evaluation metric.

### 3.2 Experimental testing of all predicted LD motif candidates

Binding affinities of LD motifs towards their biologically relevant ligands are very low, ranging from a few µM to >100 µM (Alam *et al.*, 2014; Lorenz *et al.*, 2008; Zacharchenko *et al.*, 2016). Such affinities are below the detection limit of many assays, including cell-based pull-down assays with endogenous ligand proteins. Moreover, LD motif interactions are tightly controlled in the cell, and only occur under specific conditions (Alam *et al.*, 2014). Therefore, we combined several orthogonal binding methods to robustly assess the interaction between synthesized peptides and recombinant LDBDs *in vitro*, namely (i) differential scanning fluorimetry (DSF), (ii) a direct anisotropy (DA) assay with labelled candidate peptides, (iii) an anisotropy competition assay (ACA) where unlabelled candidate peptides compete against fluorescently labelled known LD motifs, (iv) isothermal titration calorimetry (ITC) and (v) microscale thermophoresis (MST). Additionally, nuclear magnetic resonance (NMR) was used in special cases to map binding sites (for more details see Supplementary Material).

We tested the affinity of the candidate LD motifs towards two recombinant human LDBDs with broad binding characteristics: firstly, the four-helix FAT domain from FAK (residues 892–1052) that can bind two LD motifs simultaneously (on opposite sides of the helix bundle) (Gao *et al.*, 2004; Hoellerer *et al.*, 2003). FAT binds paxillin LD motifs 1, 2 and 4, but also DLC1 (Li *et al.*, 2011; Zacharchenko *et al.*, 2016). Secondly, we used the second CH domain of α-parvin (α-parvin-CH_C_, residues 242–372), which is a helical domain structurally distinct from FAT domains. This CH domain has one LD motif binding site that interacts with paxillin LD1, 2 and 4 (Lorenz *et al.*, 2008).

**Fig. 3.**
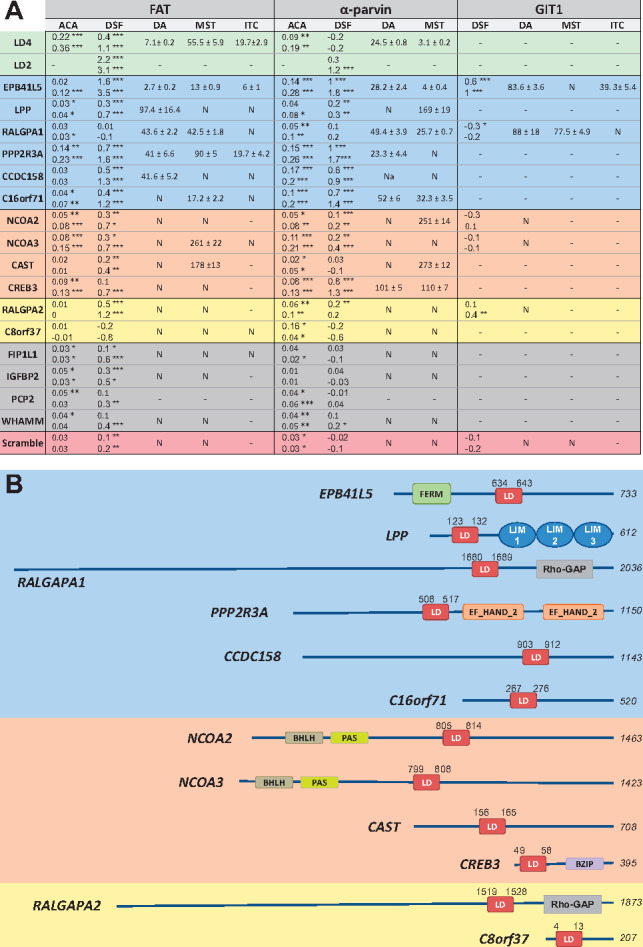
LD motif containing proteins identified in the human proteome. (**A**) Summary of experimental binding assays between putative LD motifs and selected LDBDs. The LD motifs are coloured according to: positive controls (green), negative controls (red), highly likely (blue), less likely (orange), least likely (yellow) and the motifs discarded in round 1 (grey). Values indicate the *K_d_* in µM for direct anisotropy (DA), microscale thermophoresis (MST) and isothermal calorimetry (ITC). ‘N’: no confident *K_d_* could be derived from fitting the data. ‘-’: not determined. For anisotropy competition assay (ACA) and differential scanning fluorimetry (DSF), results are given as relative difference, or indicate *Tm* shifts, respectively. For ACA and DSF values, a *t*-test of significance was performed (*n* = 3), were the null hypothesis is rejected with 95% (*), 99% (**) and 99.9% (***) of confidence. For *K_d_* values, errors are indicated as SEM. (**B**) LD motif containing proteins identified in the human proteome. Protein length and positions of the LD motifs (residues −1 to +8) are labelled. Additional domains are indicated by their PFAM name. Background colouring as in (A)

We decided not to use a simple affinity cut-off as a measure of biological relevance, because, on the one hand, some bona fide LD motifs bind LDBDs with *K_d_*’s above 100 µM (Alam *et al.*, 2014; Lorenz *et al.*, 2008), and we cannot rule out that the lowest binders in our assays have higher affinities towards LDBDs that we did not test. On the other hand, our sensitivity score of ∼89% is not high enough to completely rule out false positives. Consequently, we ranked the peptides into (i) highly likely (*K_d_* values from 1 to 99 µM in quantitative methods, and significant signal in at least one of the qualitative methods); (ii) less likely (*K_d_* values >100 µM and significant signal in at least one of the qualitative methods); (iii) least likely (all others) (Fig. 3A, Supplementary Fig. S3).

### 3.3 Scan of the human proteome using *LDMF*

In the first scan of the human proteome, used for iterative training of our tool, the initial *LDMF* version predicted 13 new LD motifs (Supplementary Table S3). Those with the strongest experimental affinity for FAT and α-parvin (EPB41L5, RALGAPA1, C16orf71, LPP) were included in the training set for the second round of model building. The final *LDMF* tool predicted eight LD motifs in addition to the training set, five that were already predicted in the first round (C8orf37, RALGAPA2, NCOA2, NCOA3 and CAST) and three new ones (PPP2R3A, CCDC158 and CREB3). The final *LDMF* effectively discarded the four first-round candidates with the lowest experimental binding signals (FIP1L1, WHAMM, IGFBP2, PCP2). Thus, *LDMF* suggested 12 new LD motif containing proteins in the human proteome. *In silico*, these 12 LD motif candidates showed the required features of bona fide LD motifs (α-helices located in unstructured and solvent exposed regions, not in a folded core) (Fig. 3B, Supplementary Fig. S4). Experimental testing supported six candidates as ‘highly likely’ (EPB41L5, PPP2R3A, RALGAPA1, C16orf71, LPP, CCDC158), four as ‘less likely’ (NCOA2, NCOA3, CAST, CREB3) and two as ‘least likely’ (C8orf37, RALGAPA2) (Fig. 3, Supplementary Fig. S3).

### 3.4 *LDMF*-identified LD motif proteins are functionally similar to bona fide LD motif proteins

Several of the *LDMF*-identified proteins showed strong functional similarity to known LD motif proteins and their cellular ligands. For example, EPB41L5 (*Band 4.1-like protein 5*, also called YMO1 and LIMULUS, or yurt in *Drosophila*) contains an N-terminal FERM domain (like FAK and Pyk2), localizes to FAs where it controls actomyosin contractility and FA maturation (Schell *et al.*, 2017). LPP (*Lipoma-preferred partner*) shows high similarity to paxillin in that it contains LIM domains, plays a structural role in the (dis)assembly of cell adhesions and shuffles between the nucleus and cell adhesions (including FAs) (Petit *et al.*, 2003). Only two of the *LDMF*-identified proteins were completely uncharacterized, namely the *Coiled-coil domain-containing protein 158* (CCDC158) and C16orf71. The LD motifs identified in the nuclear receptor coactivators 2 and 3 (NCOA2, NCOA3) were not part of their other SLiMs with a highly similar consensus sequence, namely the nuclear receptor box (NR box; LXXLL) motif and the CREBBP/CBP-binding LLXXLXXXL motif (see Supplementary Material and Supplementary Table S4 for an extended description of all *LDMF*-identified human proteins).

To quantify the overall functional similarity between novel and known LD motifs, we used gene ontology (GO) analysis. The distribution of GO semantic similarity between predicted and known LD motif proteins was significantly different from the distribution between predicted and all human proteins (*P*-value = 6.32e^−10^, Mann-Whitney test; Supplementary Fig. S5). Especially, GO terms with a dispensability value (i.e. the semantic similarity threshold at which the term was removed from the list and assigned to a cluster) of zero were the same for the predicted and known LD motif proteins for both biological processes (BP: regulation of cell morphogenesis, biological adhesion and cell-substrate adhesion) and cellular components (CC: FA, basolateral plasma membrane and cell junction). These zero-dispensability terms were similar to those of the proteins containing LDBDs (BP: signal complex assembly, biological adhesion and cell-substrate adhesion; CC: FA, basolateral membrane, cell junction and organelle). We conclude that the *LDMF*-predicted LD motif proteins show high functional similarity to the known LD motif proteins and to LDBD-containing proteins.

### 3.5 Identification of an inverse LD motif consensus

**Fig. 4.**
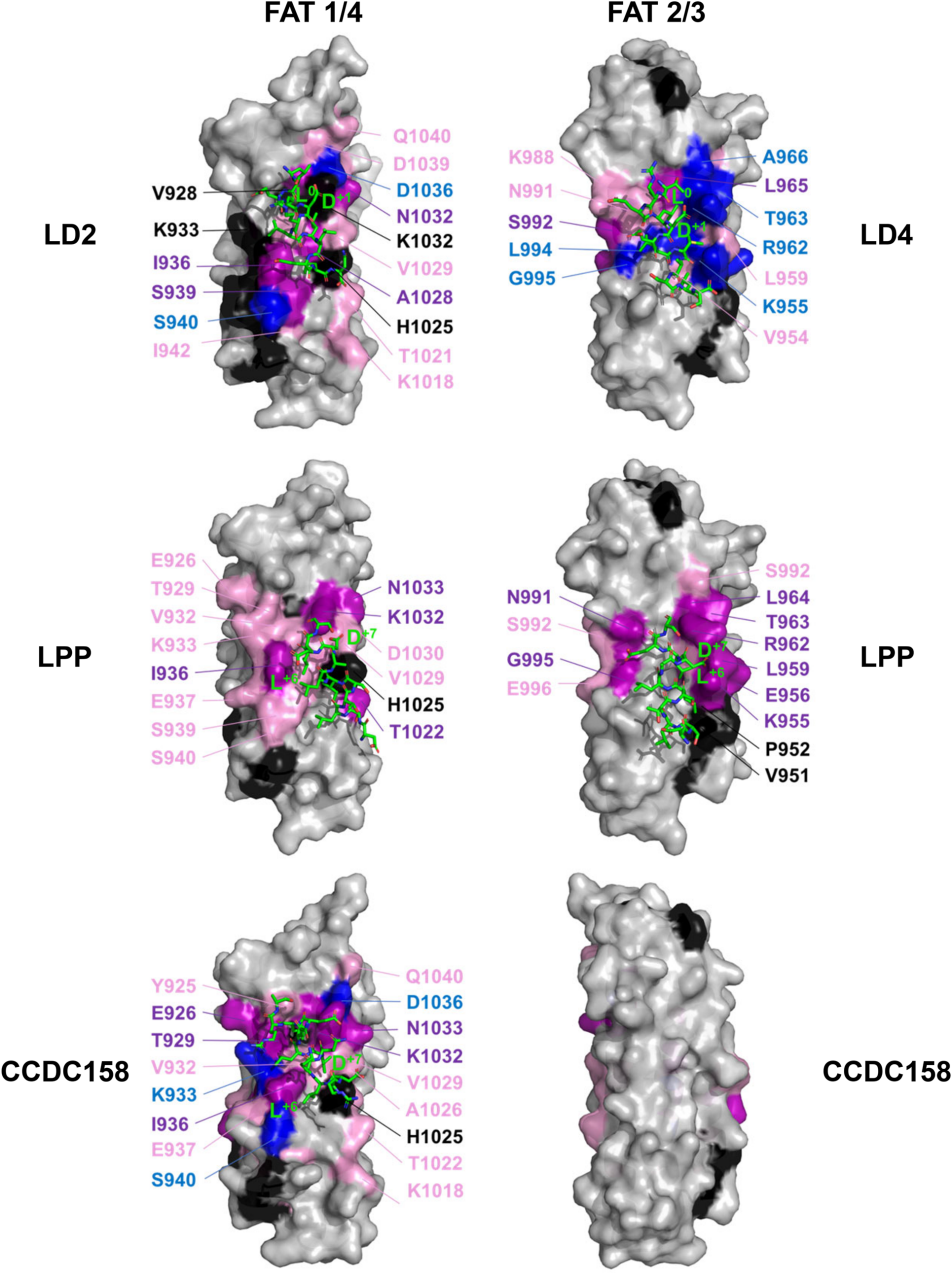
NMR binding site mapping of LD motifs onto the FAK FAT domain. NMR chemical shift changes introduced by titrations with LD motif peptides were mapped onto the molecular surface (grey) of the FAT structure in blue (resonances disappeared), purple (shift changes great than 2 σ) and pink (chemical shift changes between 1 and 2 σ). Unassigned residues and prolines were coloured black. Two sides of the FAT domain are shown: the side composed of helices 1 and 4 (1/4) and the side composed of helices 2 and 3 (2/3). LD motifs are shown as stick models, with carbons coloured in green. Paxillin LD2 and LD4 peptides were taken from the crystal structures 1ow8 and 1ow7, respectively. Positions of LD motifs of LPP and CCDC158 were obtained by NMR-data guided docking. Positions ‘L^0^’ and ‘D^+1^’ of the canonical class I consensus, and positions ‘L^+7^’ and ‘D/E^+6^’of the inverse class II are labelled

Surprisingly, the predicted and experimentally confirmed core LD motif sequences of LPP (SLTSILADLE) and CCDC158 (DLKQLLQELR) did not contain the consensus L^0^D motif. Rather, both sequences contained an LD/E motif in reverse orientation (D/E^+6^L^+7^). Titration with LPP or CCDC158 LD motif sequences caused similar NMR chemical shift changes on 15N-labelled FAT titrated as LD2 or LD4, with LPP showing a preference for the site 2/3, whereas CCDC158 only bound site 1/4 (Fig. 4, Supplementary Fig. S6). These similarities strongly suggested that LPP and CCDC158 occupy the canonical binding sites, despite their reversed LD motif. The pseudo-palindromic nature of the helical LXXLLXXL pattern would allow a reverse LD motif to engage similar electrostatic interactions to common LD motifs if the reverse motif bound in the opposite (−) orientation. Paxillin LD1 (DLDXLLXDLE) has already been shown to bind LDBDs in the opposite direction compared to leupaxin LD1 (ELDXLLXELE) and paxillin LD2 and LD4 (Alam *et al.*, 2014; Vanarotti *et al.*, 2016). Such (+) and (−) binding poses also occur in interactions of proline-rich peptides (Ladbury and Arold, 2011) and NES (Fung *et al.*, 2015). Indeed, NMR-data guided *in silico* docking produced plausible low-energy reverse orientation LPP: FAT and CCDC158: FAT models (Fig. 4).

### 3.6 Prevalence and origin of LD motifs in non-human eukaryotes

**Fig. 5.**
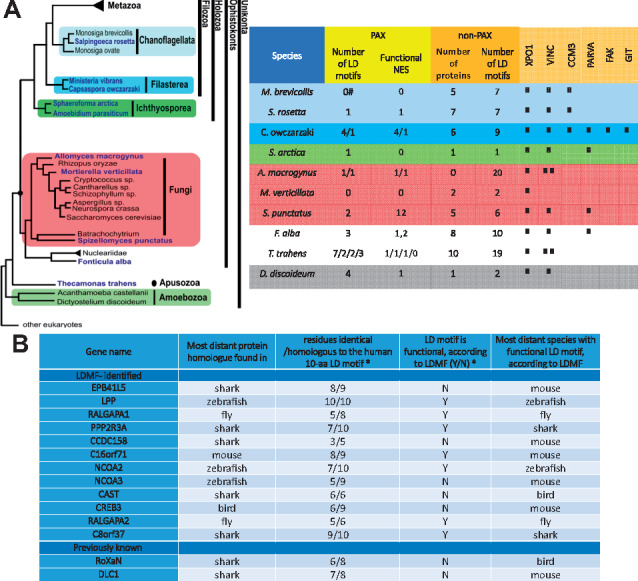
Evolution and adaptation of the LD motif interactome. (**A)** *LDMF*-predicted LD motifs and LDBDs in stem eukaryotes. *Left:* Evolutionary relation of the unicellular eukaryotes analysed. Figure adapted from the Broad Institute’s *Origin of Multicellularity* initiative. *Right:* PAX refers to paxillin homologues, non-PAX to proteins not homologous to paxillin; number of LD motifs (or LD motif containing protein) and NES as identified by LDMF and NetNES (la Cour *et al.*, 2004), respectively. If there are several paxillin homologues in one species, the corresponding numbers are separated by ‘/’. (#): species contains a paxillin homologue without LD motif. The XPO1, VINC, CCM3, PARVA, FAK and GIT columns show the presence of genes homologous to exportin, vinculin, CCM3, α-parvin, FAK and GIT, respectively. The number of ticks corresponds to the number of homologues found. The presence of a functional LDBD in these domains was assessed by sequence alignments and homology modelling. Colouring of the rows for each species matches panel A. (**B**) Conservation of the non-paxillin LD motifs. ‘Distant’ refers to the evolutionary distance to humans. *: with respect to the protein sequence in the most distant species. This table summarizes results of Supplementary Figure S8

Although the function of LD motifs is important for cell-matrix adhesion in metazoans, *LDMF* identified between 1 and 20 non-paxillin LD motif proteins in unicellular stem eukaryotes (Fig. 5A). Whereas some of these proteins were conserved between unicellular eukaryotes, especially those proteins containing kinase domains, the majority was species-specific (39 out of 53 sequences; Supplementary Fig. S7). Paxillin-like LD motif containing proteins were identified in all but two species, *Monosiga brevicollis* and *Mortierella verticillata* (Fig. 5A). Of the six LDBD proteins investigated, XPO1 was found in all proteomes, as expected from its nuclear export function (Fig. 5A;Supplementary Fig. S7). Vinculin was only absent in *M. verticillata*, which had also lost all LDBD proteins tested, except for XPO1 (Fig. 5A;Supplementary Fig. S7). The only two *LDMF*-identified non-paxillin LD motifs for this species were predicted to also function as NES, suggesting that *M. verticillata* had lost LD motif signalling and that the identified LD motifs were retained because of their NES function. Conversely, despite having lost paxillin, *Mo. brevicollis* encoded for two LDBD proteins (vinculin and CCM3) and five predicted LD motif proteins, suggesting that LD motif signalling remains used even in absence of paxillin. All proteins and their accession numbers are provided in Supplementary Table S5.

Strikingly, paxillin was also the only LD motif protein from unicellular stem eukaryotes that had a human homologue. Therefore, we next used *LDMF* to investigate the origin of the human LD motif arsenal. Neither the 12 *LDMF*-identified non-paxillin human LD motifs, nor the 2 non-paxillin human LD motifs from RoXaN and DLC1 had homologues in unicellular yeast; only 2 were found in flies (RALGAPA1, RALGAPA2) and most first appeared in fish (Fig. 5B, Supplementary Fig. S8). Where present, the *LDMF*-identified LD motif sequence generally was more conserved than the flanking residues, as expected for an SLiM. Seven out of the 14 proteins tested had functional LD motifs (according to *LDMF*) already in the most distant species (with reference to humans) in which they were found. We found that in most instances where the LD motif sequence was not predicted to be functional, this sequence was identified as functional NES. In many cases, the transformation into an LD motif (with or without losing the NES function) was achieved by only a few amino acid substitutions. NES have an α-helical secondary and a loose consensus of LXXXLXXLXL, where the X position is highly degenerate and can tolerate both acidic and bulky hydrophobic residues (la Cour *et al.*, 2004), thus offering several possibilities to harbour an overlapping LD motif.

Collectively, our data suggested that the LD signalling pathway originated before the split between amoebozoa and ophistokonts from a core module formed by paxillin and vinculin. Non-paxillin LD motifs appear or disappear frequently among species, with NES being a possible source for *de novo* LD motifs.

### 3.7 Cellular effects caused by the introduction of additional LD motifs

Given our evidence for the *de novo* appearance of LD motifs in several species, we wanted to experimentally assess the consequences of introducing additional LD motifs in cells, as would occur during evolution. We designed enhanced GFP (eGFP)-fused constructs that contained four LD motifs (akin to paxillin family members), separated by flexible linkers (see Materials and methods). In addition to the eGFP-fused tetra-LD4 motif (^eGFP^LD4), we included ^eGFP^EPB41L5 (which had the highest *in vitro* affinity for LDBDs), ^eGFP^LPP (the inversed LD motif) and ^eGFP^C16orf71 (the least characterized of the ‘highly likely’ candidates).

**Fig. 6.**
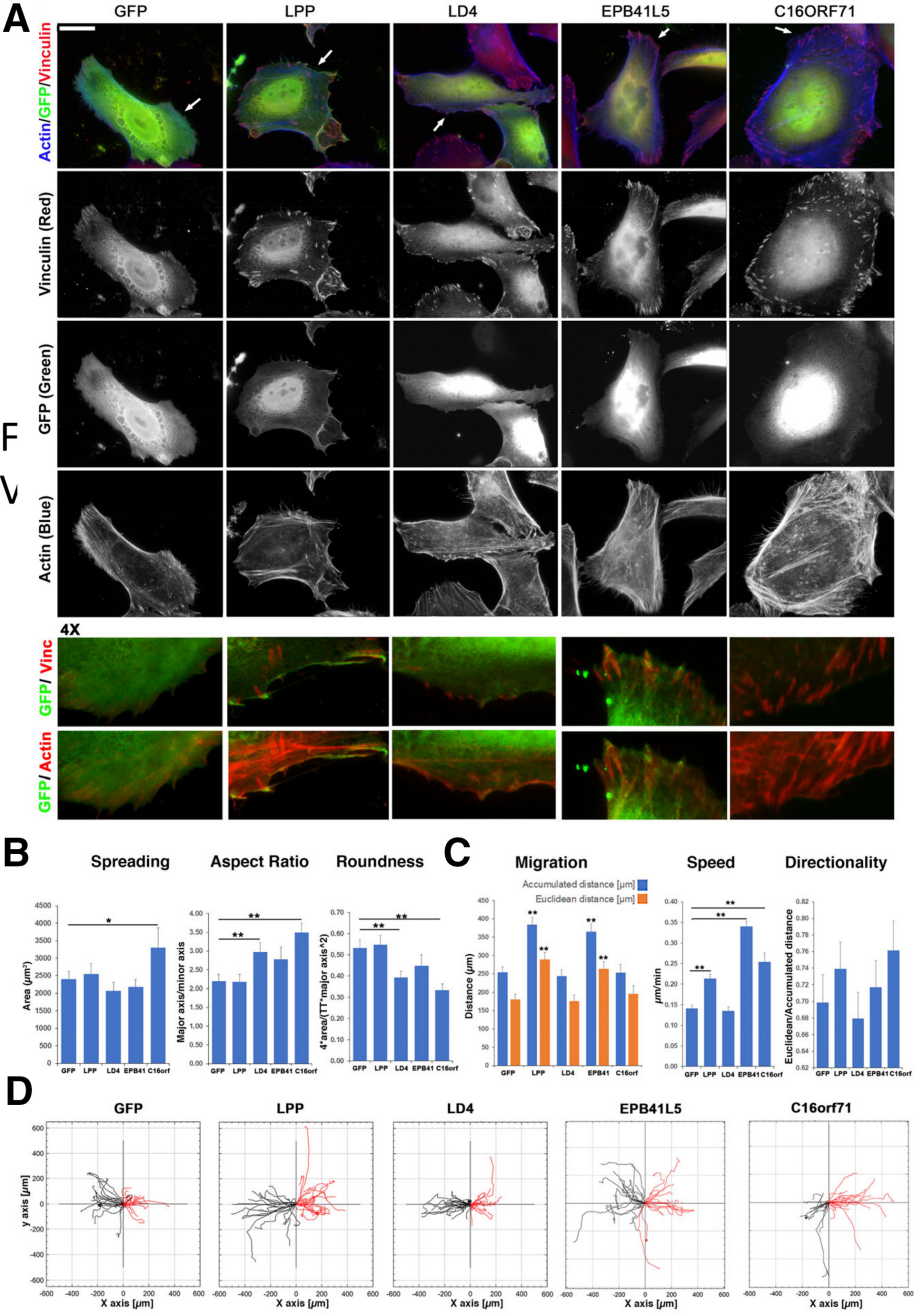
Cellular effects caused by the introduction of additional LD motifs. (**A**) Subcellular localization and Cell morphology. HeLa cells were plated on fibronectin-coated coverslips (25 000 cells) transfected and fixed after 24 h for immunofluorescence. Fixed cells incubated with the indicated antibodies and fluorescent phalloidin to reveal filamentous actin were observed with a fluorescence microscope. ‘4×’ are 2-fold enlargements of areas indicated by arrows. The enlargements show examples of the localization of eGFP-tagged proteins (GFP) in proximity of vinculin-positive FAs (Vinc.; upper panel) and actin fibres (lower panel; yellow areas). Scale bar = 50 µm. (**B**) Spreading assay. Analysis of projected cell areas (left), aspect ratio (middle) and roundness (right) were evaluated from 18 to 27 cells per condition. *P < 0.05, **P < 0.001. (**C**) Analysis of wound healing assay over 30 h: bars represent normalized mean values ± SEM of Total and Euclidean distance (left), speed (µm/min) (middle) and directionality (right; persistence of migration). **P < 0.001. The tracking profile of 35–40 moving cells per condition was quantified for the analysis of the wound healing assay. The data were analysed by the two-tailed distribution and two-sample unequal variance (Student’s *t*-test). Differences in values with *P* < 0.05 and *P* < 0.001 were considered statistically significant. (**D**) Wound healing assay using HeLa cells plated on fibronectin and transfected with eGFP-tagged constructs. Cell tracking for 30 h at 60 min/frame of eGFP control (GFP) and the indicated eGFP-tagged proteins. Black and red trajectories indicate left and right tracks, respectively

When transiently transfected in HeLa cells, ^eGFP^LD4 was less nuclear-localized and more enriched at the cell edge than eGFP alone (Fig. 6A). The cytoplasmic distribution of ^eGFP^LD4 was reminiscent to vinculin, but not as markedly enriched at FAs. Both ^eGFP^EPB41L5 and ^eGFP^LPP displayed the same cellular distribution as ^eGFP^LD4. The exogenous expression of LD4 and LPP motifs decreased the size and density of peripheral adhesions, while ^eGFP^EPB41L5-positive cells showed an enlargement of FAs and an increased overlap between vinculin and actin filaments at the cell cortex (Fig. 6A). Conversely, ^eGFP^C16orf71 had a pronounced nuclear localization and was absent from the cell edge (Fig. 6A). ^eGFP^C16orf71 was also the only of the tested sequences that significantly enhanced both the total cell area and elongation (Fig. 6B), which might be indicative of an aberrant Rho GTPases signalling as suggested by a higher density of actin stress fibre meshworks (Fig. 6A and Supplementary Fig. S9). Finally, a wound healing assay showed an enhanced cell motility and velocity of ^eGFP^LPP and ^eGFP^EPB41L5-positive cells, whereas the expression of C16orf71 increased cell speed (Fig. 6C and D).

We concluded that the simple *de novo* introduction of LD motifs already resulted in features associated with alterations in cell adhesion or spreading. These effects were mild and depended on the LD motif sequences used.

## 4 Discussion

We produced a bioinformatic tool, named *LDMF*, that allowed the proteome-wide detection of LD motifs with high accuracy. The computational detection of SLiMs is a challenging task because of their short and often degenerate consensus motifs. In our case, an additional difficulty was the scarcity of the positive dataset (i.e. known LD motifs), which precluded using deep learning methods. We solved this problem by iteratively combining computational and experimental approaches, to enhance the positive dataset, and by choosing an SVM machine-learning method adapted to the imbalanced training dataset. We showed that despite of the small number of positive samples, our novel multi-iteration active learning model increased the difficulty level of the negative samples and enhanced the positive samples through *in vitro* experiments, and can thus effectively capture the sequential and structural characteristics of LD motifs.

With this tool, together with experimental and bioinformatic analyses, we identified 12 new LD motif containing proteins in the human proteome. In comparison, only three new human LD proteins were discovered using classical experimentation since 1996. Given that a detailed cellular or *in vivo* functional analysis for all candidates was beyond the scope of this manuscript, we cannot rule out that some new LD motifs are false positives, in particular the ones with lowest experimental evidence for binding (RALGAPA2 and C8orf37). Conversely, not having experimentally tested all known LDBDs may open the possibility for false negatives. However, at least *in vitro*, there are only very little differences in binding affinity of paxillin-like LD motifs towards different LDBDs (Alam *et al.*, 2014), and hence the three LDBDs (with a total of four LD binding sites) we used appear sufficient to capture general LD motif: LDBD interactions. Similar limitations also apply to the assignment of NES. Although the algorithm used is robust (la Cour *et al.*, 2004), and the dual function of several LD motifs as NES is well documented, we cannot exclude some assignment errors. However, given that we assessed the NES function of LD motifs predicted by our tool, the tested sequences were also identified as being accessible to ligands (rather than being buried inside a protein core). This additional independent check should further rule out false positives. Importantly, given the large scale of our analysis, our general observations and conclusions are not affected by the presence of some false positives or negatives.

We demonstrated that LD motif signalling evolved in unicellular eukaryotes more than 800 Myr ago, with paxillin and vinculin as core constituents, and suggest NES as a likely source of LD motifs. Such a pre-metazoan origin is supported by the identification of integrin signalling components in unicellular eukaryotes (Sebe-Pedros *et al.*, 2010), and of paxillin homologues in the amoeba *Dictyostelium* *discoideum (*Bukharova *et al.*, 2005*)*.

Our analysis showed that LDBD proteins as well as previously known and *LDMF*-predicted LD motif proteins form a functionally homogenous group, all being involved in cell morphogenesis and adhesion. Indeed, we observed that even isolated LD motif sequences can create similar cellular effects, albeit mild, when transfected into HeLa cells as eGFP fusions. A possible explanation for this intrinsically narrow functional range of LD motifs is that its cellular role is determined by the functional range of its binding domain-containing proteins. Thus, the *de novo* creation (or loss) of LD motifs within NES appears to be a versatile and tolerable means for species-specific adaptation of cell spreading and motility. In particular, the possibility for achieving SLiMs with dual recognition by the nuclear export machinery and adhesion proteins may have provided opportunities for functionally linking cell attachment and motility with nuclear events, such as gene expression.

Collectively, our integrated computational and experimental analysis sheds light onto the origin, evolution and prevalence of a poorly understood SLiM that plays central roles in cancer cell spreading and cardiovascular diseases. The methodology used to produce our *LDMF* tool can be applied to other SLiMs for which only few representatives are known.

## Supplementary Material

btz703_Supplementary_Data
