# Supplementary material for: Proteome-level assessment of origin, prevalence and function of leucine-aspartic acid (LD) motifs
Source: Bioinformatics. 2019 Oct 4;36(4):1121–8. doi: 10.1093/bioinformatics/btz703 (PMC7703752; doi:10.1093/bioinformatics/btz703)
Supplement: btz703_Supplementary_Data [file btz703_supplementary_data.pdf]

Supplementary Material for:

Proteome-level assessment of origin, prevalence and function of Leucine-Aspartic Acid (LD) motifs

Tanvir Alam[1,6,#], Meshari Alazmi[1,8,#], Rayan Naser[2#], Franceline Huser[2#], Afaque A. Momin[2#], Veronica Astro[4], SeungBeom Hong[2], Katarzyna W. Walkiewicz[2,7], Christian G. Canlas[5], Raphaël Huser[3], Amal J. Ali[4], Jasmeen Merzaban[4], Antonio Adamo[4], Mariusz Jaremko[4], Łukasz Jaremko[4], Vladimir B. Bajic[1*], Xin Gao[1*], Stefan T. Arold[2*]

1. King Abdullah University of Science and Technology (KAUST), Computational Bioscience Research Center (CBRC), Division of Computer, Electrical and Mathematical Sciences & Engineering (CEMSE), Thuwal 23955-6900, Saudi Arabia.

2. King Abdullah University of Science and Technology (KAUST), Computational Bioscience Research Center (CBRC), Division of Biological and Environmental Sciences and Engineering (BESE), Thuwal 23955-6900, Saudi Arabia.

3. King Abdullah University of Science and Technology (KAUST), Division of Computer, Electrical and Mathematical Sciences & Engineering (CEMSE), Thuwal 23955-6900, Saudi Arabia.

4. King Abdullah University of Science and Technology (KAUST), Division of Biological and Environmental Sciences and Engineering (BESE), Thuwal 23955-6900, Saudi Arabia.

5. King Abdullah University of Science and Technology (KAUST), Core Labs, Thuwal, 23955-6900, Saudi Arabia.

* Correspondence should be sent to STA: stefan.arold@kaust.edu.sa or XG: xin.gao@kaust.edu.sa or VBB: vladimir.bajic@kaust.edu.sa

# Contributed equally

6. Present address: Information and Computing Technology Division, College of Science and Engineering, Hamad Bin Khalifa University (HBKU), Qatar Foundation, Education City, Doha, Qatar

7. Present address: NanoTemper Technologies GmbH, Floessergasse 4, Munich, Germany

8. Present address: College of Computer Science and Engineering, University of Hail, P.O. Box 2440, Hail, 81481, KSA

Short Title:

Proteome-wide prediction and function of LD motifs

KEYWORDS

focal adhesion; protein-ligand interaction; nuclear export signal; machine-learning; evolution

Content:

Supplementary Material

Supplementary Methods

Supplementary Figures S1 – S9

Supplementary Tables S1 – S5

Supplementary Material

Extended description of LDMF-identified proteins

The following characterisation of all LDMF-identified proteins was obtained by combining literature searches with four computational methods, namely PrePPI (a Bayesian framework that combines structural, functional, evolutionary and expression information (Zhang, et al., 2012)), GeneFriends (an RNAseq-based gene co-expression network (van Dam, et al., 2015)), and CoCiter (which evaluates the significance of literature co-citations (Qiao, et al., 2013)). The results from PrePPI, GeneFriends and CoCiter are compiled in **Supplementary Table 3**.

Proteins with highly likely LD motif sequences:

Band 4.1-like protein 5 (EPB41L5): The EPB41L5 peptide showed interactions in all methods used, and displayed highest affinities of all LD motifs tested (**Fig. 3A)** towards both FAT and α-parvin. PrePPI probability scores for the interaction between EPB41L5 and FAK, PYK2 or Talin are >0.9 (**Supplementary Table 3**). EPB41L5 (also called YMO1 and LIMULUS, or yurt in Drosophila) contains an N-terminal FERM domain and a C-terminal flexible region, which harbours the predicted LD motif (residues 634-643) (**Fig. 3B**). EPB41L5/yurt is a critical regulator of the lateral membrane-associated cytoskeleton. It promotes focal adhesion formation by stimulating the interaction between paxillin and integrin. It localizes to focal adhesions where it controls actomyosin contractility and FA maturation (Schell, et al., 2017)

Lipoma-preferred partner (LPP). The LPP peptide showed qualitative and quantitative binding to both FAT and α-parvin. Interactions between LPP and vinculin, α-parvin, PYK2 or GIT1 are suggested by GeneFriends and CoCiter scores (**Supplementary Table 3**). LPP is a scaffolding protein that plays a structural role in the (dis)assembly of cell adhesions and may be involved in signal transductions from adhesion sites to the nucleus, thus affect activation of gene transcription (Petit, et al., 2000; Petit, et al., 2003). LPP shows many similarities to paxillin family proteins: (i) Its N-terminal half is predicted to be an unstructured region harbouring proline-rich and phospho-tyrosine/threonine sequences in addition to the putative LD motif (residues 123-132); (ii) its C-terminal half contains three LIM domains. (iii) LPP shuffles between the nucleus and cytoplasm and is found at cell adhesions, including focal adhesions; (iv) the putative LPP LD motif sequence overlaps with a functional NES (Gorenne, et al., 2006; Gorenne, et al., 2003; Petit, et al., 2000; Petit, et al., 2003), supported by a PrePPI score of 0.98 for the LPP:XPO1 interaction. Moreover, LPP and FAK appear genetically linked (Gorenne, et al., 2006). LPP and vinculin co-localise at focal adhesions and overexpression of the LPP LIM domains displaces LPP and vinculin from these structures (Gorenne, et al., 2003; Petit, et al., 2000).

Ral GTPase-activating protein subunit alpha-1 (RALGAPA1). The RALGAPA1 peptide showed qualitative and quantitative binding to both FAT and α-parvin. PrePPI scores suggested RGPA1 interactions with FAK (0.76) and PYK2 (1.0). RALGAPA1 obtained a good co-expression score with the ARF GTPase–activating protein GIT2, and, in additional DA and MST experiments, bound with a K_d of ~80 μM to the GIT1 FAH domain (GIT1 and GIT2 are close homologues and have an identical LD motif binding site). RALGAPA1 (also called GARNL1 or TULIP1) is the catalytic α1 subunit of the heterodimeric RalGAP1 complex. RALGAPA1 functions as an activator of Ras-like small GTPases, including RalA and RalB (Shirakawa, et al., 2009). Activated RalA is involved in cell proliferation, migration, and metastasis. The suggested LD motif resides 100 amino acids upstream of the RapGAP domain, in a region predicted to be flexible (residues 1680-1689).

Serine/threonine-protein phosphatase 2A regulatory subunit B'' subunit alpha (PPP2R3A). The PPP2R3A peptide showed qualitative and quantitative binding to both FAT and α-parvin. The predicted LD motif is located in a flexible region upstream of double EF hand

domains (residues 508-517). PPP2R3A modulates substrate selectivity, catalytic activity and subcellular localisation of protein phosphatase 2A (PP2A) (UniProt, 2015). Indirect evidence suggests that PP2A promotes FAK phosphorylation (Kawada, et al., 1999; Moscardo, et al., 2013), interferes with the DLC1:FAK interaction (Ravi, et al., 2015), and is linked to focal adhesion proteins (Ito, et al., 2000).

Coiled-coil domain-containing protein 158 (CCDC158). The predicted LD motif in CCDC158 showed quantitative and qualitative binding to FAT and interacted specifically with the 1/4 site of FAT in NMR. We only measured significant qualitative binding to α-parvin. CCDC158 is an 1113-residue protein that contains 3 extended coiled-coil domains. The predicted LD motif region (residues 903-912) is located in a flexible region between the second and third coiled-coil. No literature is available for CCDC158.

C16orf71 (C16orf71). C16orf71 is an uncharacterised protein of 520 residues. It is predicted to be mostly disordered, and the predicted LD motif region, which is located in the centre of the protein (residues 267-276), showed qualitative and quantitative binding to both FAT and α-parvin.

Proteins with less likely LD motif sequences:

Nuclear receptor coactivator 2 [NCOA2, or steroid receptor coactivator 2 (*SRC-2*)]. NCOA2 is structurally mostly disordered and contains four nuclear receptor box (NR box) LXXLL motifs that mediate hormone-dependent co-activation of several nuclear receptors. A LLXXLXXXL motif in NCOA2 is involved in binding and transcriptional coactivation of CREBBP/CBP (Stashi, et al., 2014). The putative LD motif identified by *LDMF* (residues 805-814) is not part of these motifs, despite the similar consensus. Paxillin family proteins also bind to nuclear receptors, such as the androgen receptor and glucocorticoid receptor (Alam, et al., 2014). The region encompassing the putative NCOA2 LD motif is also predicted to function as an NES, akin to several paxillin LD motifs. NOCA2 obtained a strong CoCiter p-value (0.007) for association with PYK2, and has a large co-expression correlation with GIT2, but failed to show binding to GIT1 FAH in our additional experiments.

Nuclear receptor coactivator 3 (NCOA3, or *SRC-3).* Akin to NCOA2, NCOA3 is a scaffolding protein with many known interactors. NCOA3 has three NR box LXXLL motifs to bind to and co-activate several nuclear receptors, and a LLXXLXXXL motif to bind CREBBP/CBP (Stashi, et al., 2014). The predicted LD motif region (residues 799-808) is not part of a known motif.

Calpastatin (CAST). CAST is a specific inhibitor of the calcium-dependent cysteine protease calpain. The proposed CAST LD motif (residues 156-165) is a helical protein-protein interaction motif located in an otherwise disordered region; it uses its hydrophobic patch to bind to a helical subdomain of calpain, thus stabilising calpain in its inhibited form (Moldoveanu, et al., 2008). Calpain participates in cell migration and anoikis, and among its substrates are Cas, talin, FAK and PYK2 (Carragher, et al., 2003; Cooray, et al., 1996). Calpain also associates with FAT (Carragher, et al., 2003). Hence a possible competitive interaction of the calpastatin LD motif with FAK and/or PYK2 interaction with CAST could potentially promote cleavage by calpain.

Cyclic AMP-responsive element-binding protein 3 (CREB3). CREB3 is a single-pass transmembrane endoplasmic reticulum (ER)-bound transcription factor involved in the unfolded protein response, in cell proliferation and migration, tumor suppression and inflammatory gene expression. The predicted LD motif region (residues 49-58) is located in a flexible acidic transcription activation region downstream of a basic leucine-zipper (bZIP) domain (residues 174-237) and the transmembrane region (residues 255-271).

Proteins with least likely LD motif sequences:

Ral GTPase-activating protein subunit alpha-2 (RALGAPA2). RALGAPA2 is the catalytic α2 subunit of the heterodimeric RalGAP2 complex. The putative LD motif (residues 1519-1528) lies in a poorly ordered region. The RALGAPA2:PYK2 interaction has a PrePPI score of 0.93, shows medium-level co-expression with GIT2, but failed to show significant binding to GIT1 FAH.

C8orf37 (C8orf37). The 207-amino acid C8orf37 protein is widely expressed, with highest levels in brain and heart, and mutations are associated with ciliopathies and retinal dystrophy (Heon, et al., 2016). The putative LD motif (residues 4-13) is in the disordered N-terminal half of the protein.

Supplementary Methods

Computational Methods

As the number of known LD motifs is small, it becomes an imbalanced dataset problem, which usually causes issues for classification methods. Therefore, we used a two-phase approach for building the prediction model. In the first phase, we considered the known LD motifs as the positive set and the remaining 10-mers extracted from these proteins as the negative set. As expected, these extracted 10-mers can be easily differentiated from the true LD motifs because they do not satisfy sequence patterns, secondary structure patterns or physicochemical patterns of the LD motifs. Therefore, a model trained based on such a trivial negative set may not be practically useful. Yet it provides us a rough predictor by assigning different weights to sequence-, secondary structure- and physiochemical- patterns. In a second phase, we used this predictor to obtain more difficult negative sets. This was done by selecting the 10-mers from the proteins in the Protein Data Bank (PDB) which satisfy some of these patterns according to the first predictor, but not all of them. We then used these new negative sets as well to train the final predictor. This results in an active learning framework to train an LD-motif predictor.

Features that characterise bona fide LD motifs in silico.

To first determine features that characterise LD motifs *in silico*, we analysed known LD motif–containing proteins using algorithms to predict protein disorder, secondary and tertiary structures. We found that established LD motifs (paxillin family, DLC1 and RoXan), as well as gelsolin's C-terminal LD-like motif are located within protein regions predicted as disordered (**Supplementary Fig. 1**). Secondary structure prediction assigned a significant α-helix likelihood to those LD motifs, in agreement with structural studies of paxillin LD motifs 1, 2 and 4, DLC1 and gelsolin (**Fig. 1C**) (Alam, et al., 2014; Hoellerer, et al., 2003; Lorenz, et al., 2008; Nag, et al., 2009; Zacharchenko, et al., 2016) (**Supplementary Fig. 1). ***Bona fide* LD motifs are therefore computationally characterized as short α-helical segments within disordered protein regions.

Initial training data set

Our model uses information from protein sequence content of data-windows of length 10AA. Such windows are denoted as core windows. A core window is shifting one residue ahead. So, if a protein has a length L >= 10 AA residues, then there are L-10+1 possible candidate core window to be considered by scanning the protein sequence as containing a putative LD motif.

By surveying the literature, the known LD motifs were found in Paxillin, Leupaxin, PaxB, Hic-5 (Tumbarello, et al., 2002), RoXaN (Vitour, et al., 2004), and DLC1 (Durkin, et al., 2007) and we selected these LD motifs. This resulted in a set of 18 genuine LD motif windows generated from six proteins. We denote this set as the set of known LD motifs (positive set PS1). All the possible windows of length 10AA from the remaining regions of the above-mentioned six proteins were selected as the core windows of the initial negative set (NS1). This produced a set of 4020 windows from six proteins that formed NS1. To consider the importance of surrounding regions of LD motifs, 20AA residues flanking regions on each side of the scanning window were analysed.

Feature extraction from protein sequences

From the set of aligned 18 windows with their flanking sequences, position frequency matrix (PFM) was constructed. If the flanking region of scanning window is shorter than 20AA (at N-terminal and C-terminal region) then the positions are filled up by a gap ('-'). PFM was then normalised to produce Position Weight Matrix (PWM) using normalisation technique analogous

to (Bajic, et al., 2003). We only consider twenty IUPAC unambiguous AA codes (http://www.bioinformatics.org/sms/iupac.html) and gap ('-') for building PWM. We built PWM from the scanning core window ($PWM_{CoreSeq}$) which consists of 10 residues, the two flanking regions each with 20 residues produces two other PWMs (PWM_{UpSeq}, $PWM_{DownSeq}$) and the whole segment (upstream flanking region + core window + downstream flanking region) of 50 (20 + 10 + 20) AA residues produces the additional PWM. Then, during the scanning of protein sequences, we matched the four PWMs with corresponding window segments to get the respective four matching scores (Bajic, et al., 2003). We also considered the average values of the mapping score from the PWM of core window ($PWM_{CoreSeq}$) and PWM of flanking regions (PWM_{UpSeq}, $PWM_{DownSeq}$). Thus, we generated five features for each window. While generating the scores from the core PWM ($PWM_{CoreSeq}$) we used our previous knowledge of the properties of *bona fide* LD motifs (Alam, et al., 2014; Hoellerer, et al., 2003). If there are no acidic residues (Asp or Glu) either at position 0 or 6, we assign the score zero to $PWM_{CoreSeq}$. Proline has a tendency to break the helix. Consequently, if there were two consecutive prolines in core motif we also assigned 0 to $PWM_{CoreSeq}$.

Feature extraction from secondary structure (SS)

We predicted the secondary structure (SS) of the whole protein using PSIPRED (McGuffin, et al., 2000) against the NR database. Each residue in the 50AA window (core + flanking regions) was tagged as belonging to helix ('H') or coil ('C') or strand ('E'). Gap ('-') was also considered for the windows near N/C-terminal of proteins. From the set of 18 windows that correspond to known LD motifs (with flanking regions), we constructed PFM matrices (analogously as mentioned in the previous section) based on SS annotation of residues. PFM was then normalized to PWM. We built the PWM from the scanning core window (PWM_{CoreSS}), the two flanking regions each with 20 residues produces two other PWMs (PWM_{UpSS}, PWM_{DownSS}) and the whole segment (upstream flanking region + core window + downstream flanking region) of 50 (20 + 10 + 20) AA residues produces the additional PWM. Using PWMs, we were able to generate five features from SS information in the analogous manner as explained in the previous section. In these cases, if the core motif part does not have any helical prediction, we assign zero to the core motif score from PWM_{CoreSS}.

Feature extraction using AAindex

From Amino Acid Index (AAindex) database (Kawashima, et al., 2008) three physiochemical properties were extracted: hydrophobicity (Backer, et al., 1992), volume, and electric charge (Fauchere, et al., 1988). For each of the 10 residues in a core window, we calculate the AAindex values of the above-mentioned three properties that produced 30 (3*10) features.

Model Development

We generated an initial model based on the initial training data. Since this model is based on data derived from only six proteins and contains a very small number (18) of known LD motifs, we extended the training set by hypothetical LD motifs and additional negative data. For this, we used a procedure (explained below that, among other things, utilizes the initial model) that is likely to generate motifs highly similar to known LD motifs. Once the training set is expanded this way, we retrained the model as we used initially.

The Initial Model

We extracted five features using primary sequence information, five features using SS information, and 30 features using AAindex for data-windows as discussed previously. Then we used a support vector machine (SVM) model (Cortes and Vapnik, 1995) with linear kernel (Shawe-Taylor and Cristianini, 2004) to build a predictive model (M1). We used 'svmtrain' function of MATLAB 2012b with default parameter setting to build the model (there was no need

to optimize parameters of the SVM model as the default setting provided an excellent performance).

LD Motifs from Homologous Proteins

As we have very limited number of known LD motifs, we tried to increase that number using standard protein-protein BLAST (blastp) hits which are similar to motifs (Altschul, et al., 1997). We used the six proteins that contain the known LD motifs for the blastp program and selected the complete sequence of the proteins with the high score of BLAST hits (E-value:1e-7, bit score > 40, against NR database). Then, we applied our M1 to identify the LD motifs from these proteins homologous to the six proteins that contained known LD motifs. In this way, we predicted 40 more LD motifs from these proteins. These additional 40 candidate LD motifs were also considered as correct and used for building our final model.

Active Learning Dataset from PDB

We downloaded a culling set (Wang and Dunbrack, 2003) of proteins from the Protein Data Bank (PDB) to enhance our negative dataset-. We predicted SS of the full chain using PSIPRED. We built three independent models from the initial dataset based on five sequence features ($M1_{seq}$), five SS features ($M1_{ss}$) and 30 AAindex features ($M1_{aaindex}$). For each of these models, we used an SVM model with linear kernel and default parameter setting.

We applied $M1_{seq}$ to the culling set to predict windows with LD motifs. These windows formed the set S_{seq}. Analogously, we generated sets S_{ss} and $S_{aaindex}$ using $M1_{ss}$ and $M1_{aaindex}$, respectively. Our hypothesis was that a window that does not belong to the intersection of these three sets is less likely to contain LD motifs. So, we included such windows in the negative set. This has resulted in 2,279 additional negative data-windows used for building the final model.

The Second Model (M2)

We extracted the features from all (18+40) positive and all (4020+2279) negative data-windows in the same fashion as discussed previously and we used an SVM with the linear kernel to build a predictive model (M2). We used 'svmtrain' function of MATLAB 2012b with default parameters setting to build the final model. This model predicts 13 new LD motif from human proteome. We applied a version of the 18-fold cross-validation (CV) to assess the model accuracy. We divided the negative set randomly into 18 disjoint subsets. At each step of CV, we excluded a different subset from the negative data and the window that corresponds to one of the 18 known LD motifs. Moreover, from the additional 40 positive data (windows) we excluded all windows from proteins homologous to the excluded one to which the known LD motif belongs. This last step is done in order to avoid dependent data in the training set. Then, the model is derived from the remaining data as described in the section above, and it was tested on the excluded data.

The Final Model

We experimentally (*in vitro*) verified the 13 new LD motifs and found that four of them show a strong binding affinity ("Highly likely" category) towards their binding partners. So, we integrate these four motifs in the roster of true LD motif and build the final model following the same method described above. This final model predicts eight LD motifs. Three were new LD motifs and five were common to previously predicted 13 LD motifs by M2. Using CV approach, mentioned in the above section, the final model achieved over 88.88% sensitivity and accuracy of 99.97% (**Supplementary Table** 1).

Validation of LDMF using Random Sets

To evaluate the robustness of our final model we tested it on random sequences generated by Sequence Manipulation Suite (Stothard, 2000). We generated 1,000 random sequences and applied the model to them. *LDMF* did not predict any LD motif in these sequences.

Availability

LDMF is available at www.cbrc.kaust.edu.sa/ldmf. For the result mentioned in this manuscript, we used the NR database for PSIPRED predictions (McGuffin, et al., 2000). But for our online *LDMF* server, due to the prohibitive time required to obtain the results from the NR database, we used UNIPROT database for PSIPRED predictions.

Bioinformatics

Prediction of protein disorder: MetaPrDos(Ishida and Kinoshita, 2007) and RaptorX (Kallberg, et al., 2012). Prediction of secondary structure: PSIPRED(McGuffin, et al., 2000) and RaptorX (Kallberg, et al., 2012). Prediction of tertiary structures: SwissModel (Schwede, et al., 2003), RaptorX (Kallberg, et al., 2012). Prediction of transmembrane helices and signal peptides: Phobius (Kall, et al., 2007). Prediction of NES: NetNES1.1 server (la Cour, et al., 2004).

Biophysical Binding Assays

Overview and Rationale

For initial high-throughput screening, we used three plate assays: 1) differential scanning fluorimetry (DSF) was chosen as a semi-quantitative label-free binding indicator; 2) a direct anisotropy (DA) assay with labelled candidate peptides was chosen to estimate the interaction affinity; and 3) an anisotropy competition assay (ACA) where unlabelled candidate peptides compete against fluorescently labelled known LD motifs, was chosen to assess whether the (unlabelled) candidate motifs bind to the same sites as the known LD motifs. For all candidates, we used microscale thermophoresis (MST) with labelled peptides as an orthogonal quantitative method. ITC was used as an additional label-free method in selected cases to provide an additional binding K_d, or binding stoichiometry. Nuclear magnetic resonance (NMR) was used in special cases to map binding sites. Peptide sequences included four to eight flanking residues outside the 10-residue core sequence. These additional residues were chosen based on homology modelling, secondary structure and disorder predictions to include helix-capping residues and residues that might additionally contact the LDBDs. Peptides were synthesized with and without a FITC-Ahx N-terminal fluorescent label.

Peptide mimics of paxillin LD4, which were used as positive controls, displayed micromolar K_d values for FAT and α-parvin as expected, and competed efficiently against labelled LD4 in ACA (**Fig. 3A, Supplementary Fig. 3**). Although the presence of LD4 resulted in a significant change in melting temperature *Tm* in DSF with FAT, the *Tm* change with α-parvin was not significant compared to a negative control (a peptide with the scrambled LD4 sequence). This result led us to include an LD2 peptide as a positive control in DSF.

Protein production

Human α-parvin-CH_C (residues 242-372), the FAT domain of human FAK (892-1052), and the rat GIT1 (647-770) were expressed as GST-fusion proteins in *E. coli* BL21 using the expression vectors pGex 6P1, pGexP2, pGex-4T1, respectively. Bacteria were grown in LB medium. α-parvin-CH_C and FAT were expressed at 20°C overnight, whereas GIT1 was expressed for 6h at 30°C, α-parvin-CH_C,FAT and GIT were purified as described previously (Arold, et al., 2002; Lorenz, et al., 2008; Schmalzigaug, et al., 2007).

Differential Scanning Fluorimetry

Experiments were performed in 20 mM HEPES pH 7.5, 150 mM NaCl, 2 mM EDTA, 1 mM TCEP. FAT, α-parvin-CH_C and GIT1 were used at a concentration of 10 μM. Protein stability was assessed for each peptide at 100 and 250 μM. SYPRO Orange was used as fluorescent

dye at 1x the protein concentration. The samples were heated from 20°C to 95°C at a rate of 0.03°C/s on a LightCycler 480 II RT-PCR from Roche. To estimate the melting temperature (Tm), a generalized sigmoid was fitted by least squares and the inflection point was computed.

Direct Anisotropy Assay

Protein was serially diluted in buffer (20 mM HEPES pH 7.5, 150 mM NaCl, 2 mM EDTA, 2 mM DTT, 0.005% Tween-20) and labelled peptides were added at a final concentration of 0.1 μM. Fluorescence anisotropy was measured on a PHERAstar FS device (BMG Labtech) using a fluorescence polarization module 485/520/520, at room temperature. Fluorescence anisotropy was determined as: $1000*(I_{//} - I_{+})/(I_{//}+2*I_{+})$, where $I_{//}$ and I_{+} are parallel and perpendicular components of fluorescence intensity excited by parallel polarized light. Data were analysed with Origin software using a logistic fit.

Anisotropy Competition Assay

First, FAT and α-parvin were titrated, and FITC-Ahx-labelled LD4 was added as described for the direct anisotropy assay. Competition for the LD4 binding site of FAT and α-parvin was then assessed as follows: the proteins were kept at a concentration corresponding to the K_d of their interaction with labelled LD4 (10 μM for FAT and 25 μM for α-parvin), in the presence of 0.1 μM labelled LD4. To that, each non-labelled peptide was added at 100 and 250 μM. When competing for the binding site, the unlabelled peptide displaces labelled LD4 resulting in a lower anisotropy. All measurements were performed as for direct anisotropy assay. Values are represented as a ratio to the point estimated to be the K_d of the protein with LD4 labelled.

Isothermal Titration Calorimetry

Proteins were dialysed in ITC buffer (20mM HEPES pH 7.5, 150mM NaCl, 1mM EDTA, 1mM TCEP). 1.5 ml of protein solution was placed in the cell at a concentration varying depending on the interaction from 50 to 150 μM for FAT and 125 μM for GIT1. Peptides were dissolved into the dialysis buffer to a concentration of between 1 to 1.25 mM and placed in the injection syringe. Titrations were performed at 25 °C. As a control, the peptide was titrated into the buffer and the resulting heats subtracted from the protein-binding curve. ITC was performed either on a Nano ITC (TA Instruments), and data were fitted using NanoAnalyze Software, or using a ITC 200 (GE) and data were fitted using Origin Software.

Microscale Thermophoresis

Serial dilutions of proteins were prepared starting from 630 μM (GIT1), 560 μM (FAT) or 530 μM (α-parvin) in reaction buffer (20 mM HEPES pH 7.5, 150 mM NaCl, 2 mM EDTA, 2 mM DTT, 0.05% Tween-20). Labelled peptides were added to a final concentration of 0.1 μM. The experiment was performed at 20 % LED power and 20, 40 and 60 % MST power in standard capillaries (GIT1) and MST Premium Capillaries (FAT and α-parvin) on a Monolith NT.115 device at 25 °C (NanoTemper Technologies). Thermophoresis and temperature jump were fitted using the K_D formula derived from the law of mass action on the provided NT analysis software.

Nuclear Magnetic Resonance

Cells were grown with ^{15}N-labelled ammonium chloride dissolved in M9 minimal media solution, induced at OD=0.8 with 300uM IPTG and harvested after incubation overnight at 22 °C. Protein samples were purified and NMR samples were prepared by dissolving the ^{15}N-labelled protein in a 10% D_2O/90% H_2O solution with a monomer concentration of 100 μL in a total volume of 500 μL and pH of 7.5. LD motif-containing peptides were dissolved with FAT gel filtration buffer (20 mM HEPES pH=7.5, 150mM NaCl, 2mM EDTA and 2mM DTT). 2 μL of 25 mM 2,2-dimethyl-2-silapentane-5-sulfonate (DSS) sodium salt was added as an internal chemical shift reference for ^{1}H at 0 ppm. The samples were stable over the course of the NMR experiments.

The ^1H-^{15}N HSQC titration experiments were performed at a temperature of 25 °C using a Bruker Avance III 950 MHz NMR spectrometer equipped with a triple resonance inverse TCI CryoProbe. Spectra were acquired with 2048 (^1H) × 200-256 (^{15}N) complex points, a spectral width of 16 ppm for ^1H and 40 ppm for ^{15}N, and averaged for 36-88 scans depending on sample concentration. Changes in chemical shifts for ^1H and ^{15}N were measured in ppm (δH and δN) in comparison to unpublished amino acid backbone NMR assignments of human FAT domain. ^{15}N shift changes were multipled by a scaling factor α=0.2, and then the total change in the chemical shift perturbations (CSPi) was calculated following this equation: $CSPi = \sqrt{\frac{1}{2}[\delta_H^2 + \left(\alpha . \delta_N^2\right)]}$ (Williamson, 2013).

Data-driven Molecular Docking

The data-driven HADDOCK 2.1 protocol (van Zundert, et al., 2016) was used to generate the models of complexes for FAT:CCDC158 and FAT:LPP. Crystal structures of FAT (1ow8 and 1ow7) were used for the modelling. Initial models for CCDC158 and LPP were modelled in helical form based on the LD4 peptide. The NMR chemical shift perturbation (CSP) data was used to define the residues, which could be potentially involved in the binding known as active residues. The residues 915, 926, 929, 933, 934, 936, 938, 940, 956, 1031, 1032, 1033, 1035, 1036 and 1038 were marked on FAT helix 1/4 as active residues for FAT:CCDC158. The residues 914, 916, 934, 936, 938, 1022, 1027, 1031, 1032 and 1033 were marked on FAT helix 1/4 and 948, 955, 956, 957, 959, 962, 963, 964, 991 and 1007 were marked on FAT helix 2/3 as active residues for FAT:LPP. The CSP data was only used to define the binding site and not the binding poses. Structures that were listed in the output clusters with best scores were further analysed using PyMol (pymol.org).

Cellular Analyses

Design and preparation of eGFP-coupled tetra LD motifs

eGFP-LD fusion constructs contained an N-terminal eGFP followed by a HRV3C protease recognition site (LEVLFQGP) and then four times the same LD motif sequence. LD motifs were separated by glycine-serine-threonine linkers of different lengths to enable multivalent associations with LDBDs: LD–GSGST–LD–GSGSTGSGST–LD–GSGSTGSGSTGSGST–LD. LD sequences were LD4: TRELDELMASLSD; LPP: EIDSLTSILADLESS; EPB41L5: ATDELDALLASLTENLID; C16orf71: EAWDLDDILQSLQGQ. Constructs were synthesized as gBlock (IDT) fragments separately for the N-terminal eGFP and the C-terminal tetra-LD motif sequences. CPEC cloning (Quan and Tian, 2009) was used to create the construct-including vectors and confirmed by the sequencing.

Cell lines, transfection, and antibodies

HeLa cells were cultured in DMEM with 10% FBS and transfected with plasmid DNA using Lipofectamine 3000. For cell spreading and immuno-localization experiments, HeLa cells were plated at low density on fibronectin-coated coverslips, transfected and used for immunofluorescence 24h later, as previously described (Astro, et al., 2011). For live cell imaging, HeLa cells were plated on fibronectin-coated 6-well plates, transfected with GFP-tagged plasmids, manually scratched and recorded 36 h after transfection. The pAb against GFP and Vinculin, and the AlexaFluor 647-conjugated phalloidin were from Thermo Scientific. Fixed cells were observed with the EVOS FL Auto 2 Microscope (Thermo Scientific) using a Plan Apochromat 1.42 NA/60X oil objective (Zeiss).

Morphological analysis and functional assays

The measurement of cell area projection, aspect ratio and roundness of transfected HeLa cells spread for 24 hours on fibronectin was evaluated on thresholded images using ImageJ. For wound healing assays, images were captured with a 10x lens at 60-min interval for 30 h using an optical microscope (JuLI™ Stage Real-Time Cell History Recorder, NanoEntek) equipped with a High-sensitivity monochrome CCD (Sony sensor 2/3") and an automated x-y-z stage, with a 0.3 NA/10X objective (Olympus). During live imaging cells were kept at 37°C and 5% CO2 in a cell incubator (Heracell, 150i, Thermo Scientific). Migration paths were calculated from the nuclear positions of GFP-positive cells obtained from 4 fields per well using two plugins available for ImageJ software (Manual tracking and Chemotaxis tool). The track of each cell was used to measure different parameters of migration: total and Euclidean distances (length of the line segment, calculated between the start and the end point of the cell trajectory), cell velocity and directionality (index of the persistence of the cell movement, given by the ratio between the Euclidean and the total distances. This value may change between 0 and 1, where 1 corresponds to the maximum linearity of the trajectory).

Supplementary Figure 1. Features that characterise *bona fide* LD motifs *in silico.*

For each known LD motif, we present the secondary structure predictions (SS3: three states, namely H: helix, E: beta strand, C: coil; SS8: eight states, namely H: α helix, G: 3-helix, I: 5-helix, E: extended β ladder, B: β bridge, T: hydrogen bonded turn, S: bend, L: loop), solvent accessibility (ACC; B: buried; M: medium exposed, E: solvent exposed) and disorder (DISO: order [.] and disorder [*]) as predicted by the RaptorX server (Kallberg, et al., 2014). Amino acid are numbered starting with 20 positions upstream of the LD motif (unless the LD motif is situated at the N-terminus, which is then taken as number 1).

1 Paxillin

LD1

	1	11	21	31
SEQ	MDDLDALLAD	LESTTSHISK	RPVFLSEETP	YS
SS3	CCHHHHHHHH	HHCCCCCCCC	CCCCCCCCCC	CC
SS8	LLHHHHHHHH	HHLLLLLLLL	LLLLLLLLLL	LL
ACC	EEEEEEEEEE	EEEEEEEEEE	EEEEEEEEEE	EE
DISO	**********	**********	**********	**

LD2

	1	11	21	31	41
SEQ	QKSAEPSPTV	MSTSLGSNLS	ELDRLLLELN	AVQHNPPGFP	ADEANSSPPL
SS3	CCCCCCCCCC	CCCCCCCCCC	HHHHHHHHHH	CCCCCCCCCC	CCCCCCCCCC
SS8	LLLLLLLLLL	LLLLLLLLLL	HHHHHHHHHH	LLLLLLLLLL	LLLLLLLLLL
ACC	EEEEEEEEEE	EEEEEEEEBE	EBMEBBEEBE	EEEEEEEEEE	EEEEEEEEEE
DISO	**********	**********	**********	**********	**********

LD3

	1	11	21	31	41
SEQ	PLTKEKPKRN	GGRGLEDVRP	SVESLLDELE	SSVPSPVPAI	TVNQGEMSSP
SS3	CCCCCCCCCC	CCCCCCCCCC	CHHHHHHHHC	CCCCCCCCCC	ECCCCCCCCC
SS8	LLLLLLLLLL	LLLLLLLLLL	LHHHHHHHHH	LLLLLLLLLL	ELLLLLLLLL
ACC	EEEEEEEEEE	EEEEEEEEEE	EBEEMBEEEE	EEEEEEEEEE	EEEEEEEEEE
DISO	**********	**********	**********	**********	**********

LD4

	1	11	21	31	41
SEQ	PQRVTSTQQQ	TRISASSATR	ELDELMASLS	DFKIQGLEQR	ADGERCWAAG
SS3	CCCCCCCCCC	CCCCHHHHHH	HHHHHHHHHH	HHHHHCCCCC	CCCCCCCCCC
SS8	LLLLLLLLLL	LLLLHHHHHH	HHHHHHHHHH	HHHHHTLLLL	LLLLLLHLLL
ACC	EEEEEEEEEE	EEEEEEEBEE	MBEEBBEEME	EEEEEEEEEE	EEEEEEEEEE
DISO	**********	**********	**********	**********	**********

LD5

	1	11	21	31	41
SEQ	MAQGKTGSSS	PPGGPPKPGS	QLDSMLGSLQ	SDLNKLGVAT	VAKGVCGACK
SS3	CCCCCCCCCC	CCCCCCCCCC	CHHHHHHHHH	HHHHHCCCCC	CCCCCCCCCC
SS8	LLLLLLLLLL	LLLLLLLLLL	LHHHHHHHHH	HHHHHTTLLL	LLLLLLLLLL
ACC	EEEEEEEEEE	EEEEEEEEEE	EEEEEEEEEE	EEBEEEEMEE	EEEEEEEEEE
DISO	**********	**********	**********	**********	**********

2 Leupaxin

LD1

	1	11	21	31
SEQ	MEELDALLEE	LERSTLQDSD	EYSNPAPLPL	DQ
SS3	CCHHHHHHHH	HHHHHCCCCC	CCCCCCCCCC	CC
SS8	LLHHHHHHHH	HHHHHLLLLL	LLLLLLLLLL	LL
ACC	EEEEEEEEEE	EEEEEEEEEE	EEEEEEEEEE	EE
DISO	**********	**********	**********	**

LD2

	1	11	21	31	41
SEQ	YSEAQEPKES	PPPSKTSAAA	QLDELMAHLT	EMQAKVAVRA	DAGKKHLPDK
SS3	CCCCCCCCCC	CCCCCCHHHH	HHHHHHHHHH	HHHHHHHHHH	CCCCCCCCCC
SS8	LLLLLLLLLL	LLLLLLHHHH	HHHHHHHHHH	HHHHHHHHHH	LLLLLLLLLL
ACC	EEEEEEEEEE	EEEEEEEEEE	EEEEBMEEBE	EEEEEEEEEE	EEEEEEEEEE
DISO	**********	**********	**********	**********	**********

LD3

	1	11	21	31	41
SEQ	VAVRADAGKK	HLPDKQDHKA	SLDSMLGGLE	QELQDLGIAT	VPKGHCASCQ
SS3	CCCCCCCCCC	CCCCCCCCCC	CHHHHHHHHH	HHHHHCCCCC	CCCCCCCCCC
SS8	LLLLLLLLLL	LLLLLLLLLL	LHHHHHHHHH	HHHHHTTLLL	LLLLLLLLLL
ACC	EEEEEEEEEE	EEEEEEEEEE	EEEEEBEEME	EEBEEMEMEE	MEEEEBEEEE
DISO	**********	**********	**********	**********	**********

3 Pax-B

LD1

	1	11	21	31
SEQ	MATKGLNMDD	LDLLLADLGR	PKSSIKVTAT	VQTTATPSS
SS3	CCCCCCCCCC	HHHHHHHHCC	CCCCCCCCCC	CCCCCCCCC
SS8	LLLLLLLLLL	HHHHHHHHTL	LLLLLLLLLL	LLLLLLLLL
ACC	EEEEEMEMEE	BEEBMEEMEE	EEEEEEEEEE	MEEEEEEEE
DISO	**********	**********	**********	*********

LD2

	1	11	21	31	41
SEQ	VSSQPAPQPP	QQSQQIDGLD	DLDELMESLN	TSISTALKAV	PTTPEEHITH
SS3	CCCCCCCCCC	CCCCCCCCCC	HHHHHHHHHH	HHHHHHHHCC	CCCCCCCCCC
SS8	LLLLLLLLLL	LLLLLLLLHH	HHHHHHHHHH	HHHHHHHHHL	LLLLLLLLLL
ACC	EEEEEEEEEE	EEEEEEEEEE	EEEEEMEEBE	EEBEEEEEEE	EEEEEEEEEE
DISO	**********	**********	**********	**********	**********

LD3

	1	11	21	31	41
SEQ	SQSQPQPYKV	TATNSQPSSD	DLDELLKGLS	PSTTTTTTVP	PPVQRDQHQH
SS3	CCCCCCCCCC	CCCCCCCCHH	HHHHHHHCCC	CCCCCCCCCC	CCCCCCCCCC
SS8	LLLLLLLLLL	LLLLLLLLHH	HHHHHHHTLL	LLLLLLLLLL	LLLLLLLLLL
ACC	EEEEEEEMEM	EEEEEEEEEE	EBMEMBEEME	EEEEEEEEEE	EEEEEEEEEE
DISO	**********	**********	**********	**********	**********

LD4

	1	11	21	31	41
SEQ	NTPNNNNNNN	TNSPKVVHGD	DLDNLLNNLT	SQVKDIDSTG	PTSRGTCGGC
SS3	CCCCCCCCCC	CCCCCCCCCH	HHHHHHHHHH	HHHHCCCCCC	CCCCCCCCCC
SS8	LLLLLLLLLL	LLLLLLLLHH	HHHHHHHHHH	HHHHLLLLLL	LLLLLLLLLL
ACC	EEEEEEEEEE	EEEEEEEEEE	EBEEBBEEBM	EEBEEMEEEE	EEEEEEEEEE
DISO	**********	**********	**********	**********	**********

4 HIC-5

LD1

	1	11	21	31
SEQ	MEDLDALLSD	LETTTSHMPR	SGAPKERPAE	PL
SS3	CCHHHHHHHH	HHHCCCCCCC	CCCCCCCCCC	CC
SS8	LLHHHHHHHH	HHHLLLLLLL	LLLLLLLLLL	LL
ACC	EEEMEEEEEE	EEEEEEEEEE	EEEEEEEEEE	EE
DISO	**********	**********	**********	**

LD2

	1	11	21	31	41
SEQ	AAPAAPPFSS	SSGVLGTGLC	ELDRLLQELN	ATQFNITDEI	MSQFPSSKVA
SS3	CCCCCCCCCC	CCCCCCCCCC	HHHHHHHHHC	CCCCCCHHHH	HHHCCCCCCC
SS8	LLLLLLLLLL	LLLLLLLLLL	HHHHHHHHHH	LLLLLLHHHH	HHHSLLLLLL
ACC	EEEEEEEEEE	EEEEEEEEBM	EBMEBBEEBE	EEEEEMMEEB	BEEBEEEEEE
DISO	**********	**********	**********	**********	**********

LD3

	1	11	21	31	41
SEQ	SLPSSPSPGL	PKASATSATL	ELDRLMASLS	DFRVQNHLPA	SGPTQPPVVS
SS3	CCCCCCCCCC	CCCCCCHHHH	HHHHHHHHHH	HHHHHCCCCC	CCCCCCCCCC
SS8	LLLLLLLLLL	LLLLLLHHHH	HHHHHHHHHH	HHHHHLLLLL	LLLLLLLLLL
ACC	EEEEEEEEEE	EEEEEEEEEE	MBEEBBEMBE	EEEEEEEEEE	EEEEEEEEEE
DISO	**********	**********	**********	**********	**********

LD4

	1	11	21	31	41
SEQ	PVVSSTNEGS	PSPPEPTGKG	SLDTMLGLLQ	SDLSRRGVPT	QAKGLCGSCN
SS3	CCCCCCCCCC	CCCCCCCCCC	CHHHHHHHHH	HHHHHCCCCC	CCCCCCCCCC
SS8	LLLLLLLLLL	LLLLLLLLLL	LHHHHHHHHH	HHHHHTTLLL	LLLLLLLLLL
ACC	EEEEEEEEEE	EEEEEEEEEE	EEEEEEEEEE	EEBEEEEMEE	EEEEEEEEEE
DISO	**********	**********	**********	**********	**********

5 Roxan

	1	11	21	31	41
SEQ	RTLPSTDSLD	DFSDGDVFGP	ELDTLLDSLS	LVQGGLSGSG	VPSELPQLIP
SS3	CCCCCCCCCC	CCCCCCCCCH	HHHHHHHCCC	CCCCCCCCCC	CCCCCCCCCC
SS8	LLLLLLLLLL	LLLLLLLLLH	HHHHHHHTLL	LLLLLLLLLL	LLLLLLLLLL
ACC	EEEEEEEEEE	EBEEEEEEEE	EBMEBBEEME	EEEEEEEEEE	EEEEEEEEEE
DISO	**********	**********	**********	**********	**********

6 DLC1

	1	11	21	31	41
SEQ	SILYSSSGDL	ADLENEDIFP	ELDDILYHVK	GMQRIVNQWS	EKFSDEGDSD
SS3	CCCCCCCCCC	CCCCCCCCCH	HHHHHHHHHH	HHHHHHHHHH	HHCCCCCCCC
SS8	LLLLLLLLLL	LLLLLLLLLH	HHHHHHHHHH	HHHHHHHHHH	HHLLLLLLLL
ACC	EEEEEEEEEE	EEEEEEEMME	EBMEBBEMBM	EMMEEBEEEE	EEEEEEEEEE
DISO	**********	**********	**********	**********	**********

Supplementary Figure 2. Bioinformatic features of LD motif candidates predicted by other tools.

Supplementary Figure 2.1. Computational assessment of the structural context of the 18 potential LD motifs proposed by Brown et al. (Brown, et al., 1998). These motifs were suggested based on a pattern search with the sequence pattern (L,V)(D,E)X(L,M)(L,M)XXL used by Brown et al. (Brown, et al., 1998).

Extended Results: 16 out of the 18 suggested LD motifs were predicted to be an integral part of a folded protein domain. In 15 out of these 16 cases, the hydrophobic patch of the suggested LD motif is inaccessible to solvent and hence ligands. In the one remaining case (LTK), the suggested LD motif is part of the catalytically important αC helix of a protein kinase domain. Thus, unless unlikely large unfolding events occur, these 16 putative motifs cannot function as LD motifs despite containing the correct sequence pattern. For the remaining two of the 18 proteins, the suggested LD motif sequence is located in a flexible region. However, in one case (Eph-2) the putative LD motif is part of a signalling peptide that is cleaved *in vivo*, and hence an unlikely candidate. Only the remaining LD sequence from chicken tensin was a plausible candidate, being located in an unstructured region and implicated in FAs (Lo, 2004).

Summary of Previously suggested LD motifs by Brown *et al.* (Brown, et al., 1998)

	**UNIPROT Entry**	**Motif sequence and location in protein**	**Sequence identity of 3D templates for suggested LD motif region**
1	P09104; γ-Enolase	90-LDNLMLEL-97	100 % identical; *
2	P05937; Calbindin	211-LDALLKDL-218	98 % identical; *
3	P29376; LTK	556-LDFLMEAL-563	77 % identical; *
4	P10911; DBL	662-LDAMLDLL-669	65 % identical; *
5	P22676; Calretinin	220-LDALLKDI-227	59 % identical; *
6	P55039; DRG	276-LDYLLEML-283	55 % identical; *
7	P29461; PTP2	679-LDFLLSIL-686	42 % identical; *
8	P36010; β-Adaptin	409-LDILLELL-416	40 % identity; *
9	P40421; RDG	163-LDDLLVVL-170	40 % identical; *
10	P38570; Integrin αE	375-LDGLLSKL-382	38 % identical; *
11	P52306; RAP1 GDS	27-LDCLLQAL-34	24 % identical
12	P53046; Rho1 GEF	713-LDNMLLFL-720	24 % identical; *
13	P35579; Myosin HC	1422-LDDLLVDL-1429	17 % identical; coiled-coil
14	P24216; Hap2	443-LDVLMTS-450	13 % / 43 % identical (depending on fragment length); *
15	P54762; Eph-2	3-LDYLLLLL-10	Signal peptide; no 3D template
16	P38650; Dynein HC	1361-LDGLLNQL-1368	No template
17	P51592; E3	1453-LDTLLLTL-1460	No template
18	Q04205; tensin	807-LDVLMLDL-814	No template

*: available in the Protein Model Portal www.proteinmodelportal.org.
No shading: proteins where 3D models can be established with good confidence, showing that their LD motifs are implicate in a 3D fold and hence inaccessible for canonical LD motif interactions.
Yellow shaded molecules: no high-quality model exists, but either low-identity structural homology or other functionality make an LD-motif function unlikely.
Green shading: no 3D model is available, and strong biological assumptions to rule out LD-motif function are lacking. However, known biological function speak against it, and the motif is highly degenerate.
Red shading: this motif is potentially likely to be a bona fide LD motif, because of its structural characteristics and supporting biological evidence.

Computational assessment of proposed LD motif–containing proteins (Brown, et al., 1998)

Homology models are coloured according to their secondary structure (magenta: α-helix; cyan: β-strands). The putative LD motif is colored in green, with the LD motif positions 0, 3 and 4 (L^0XXLL) colored in red. MetaPrDos (http://prdos.hgc.jp/ (Ishida and Kinoshita, 2007)) was used for predicting structural order/disorder from the protein sequence. PHOBIUS (http://phobius.sbc.su.se/ {Kall, 2007 #3127}) was used for prediction of transmembrane helices and signal peptides. The structural analysis was carried out using the SWISS-MODEL (https://swissmodel.expasy.org/ (Arnold, et al., 2006)) and RaptorX (http://raptorx.uchicago.edu/ (Kallberg, et al., 2014)) servers.

1 P09104; GAMMA ENOLASE; ENO2

Location in protein: 90-LDNLMLEL-97
Structural Information: 100% Sequence Identity with PDB 2akm. The suggested LD motif is part of the catalytic domain.

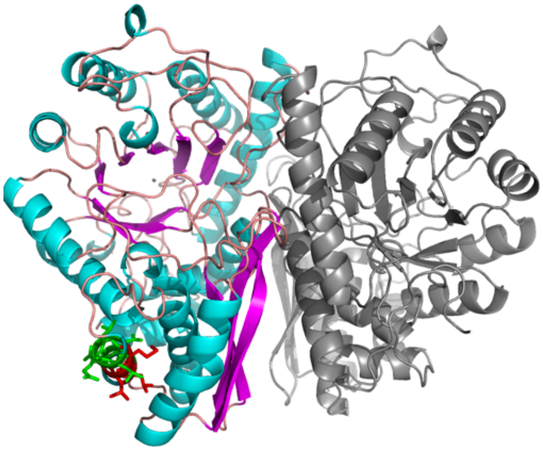

2 P05937; CALBINDIN

Location in protein: 211-LDALLKDL-218
Structural Information: 98% Sequence Identity with PDB Template 2f33A. Forms EF-hand helix-turn-helix.

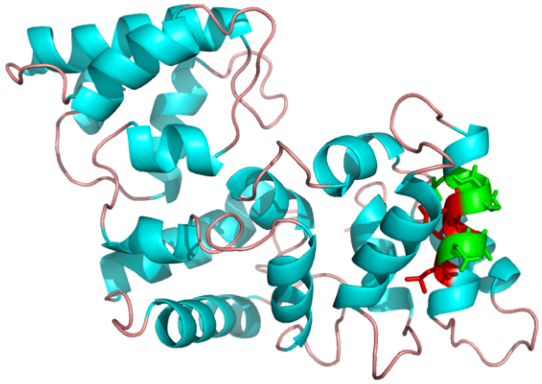

3 P29376; LTK

Location in protein: 556-LDFLMEAL-563
Structural Information: 77% Sequence Identity with PDB 3ics. The LD motif is situated in the αC helix of the protein kinase domain.

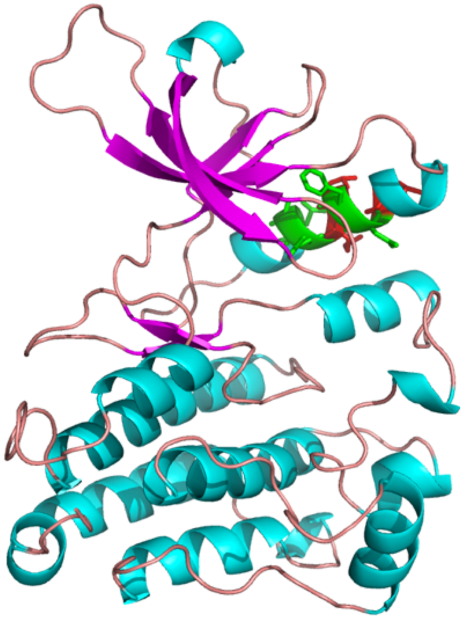

4 P10911; DBL

Location in protein: 662-LDAMLDLL-669
Structural Information: 65 % sequence identity with dbl-homology domain (DH domain); Template PDB 1kz7.

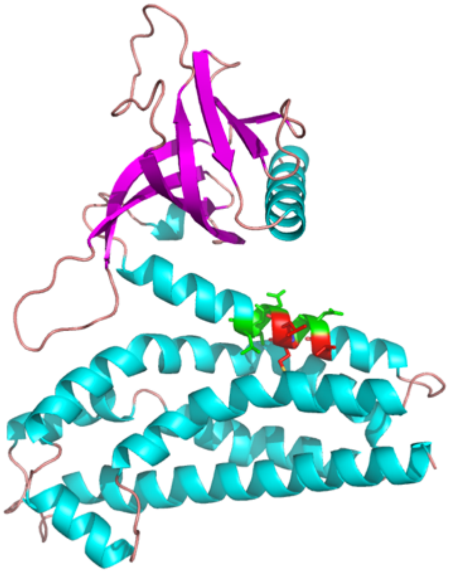

P22676; CALRETININ; CAB29

Location in protein: 220-LDALLKDI-227
Structural Information: 59 % sequence identify with PDB 2f33; forms EF-hand helix-turn-helix.

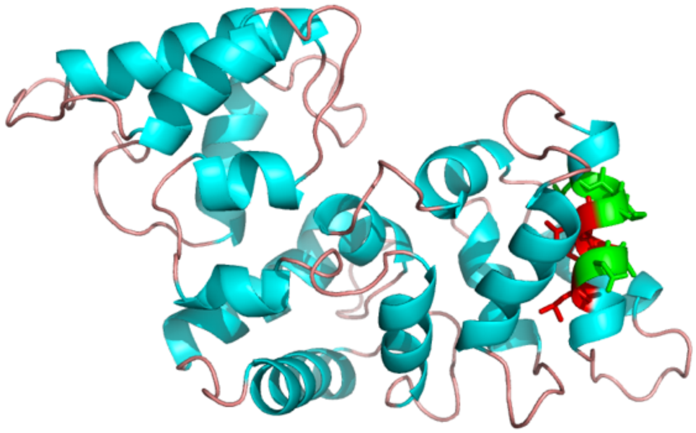

5 P55039; DRG

Location in protein: 276-LDYLLEML-283
Structural Information: 55% sequence identity with PDB 4a9a.

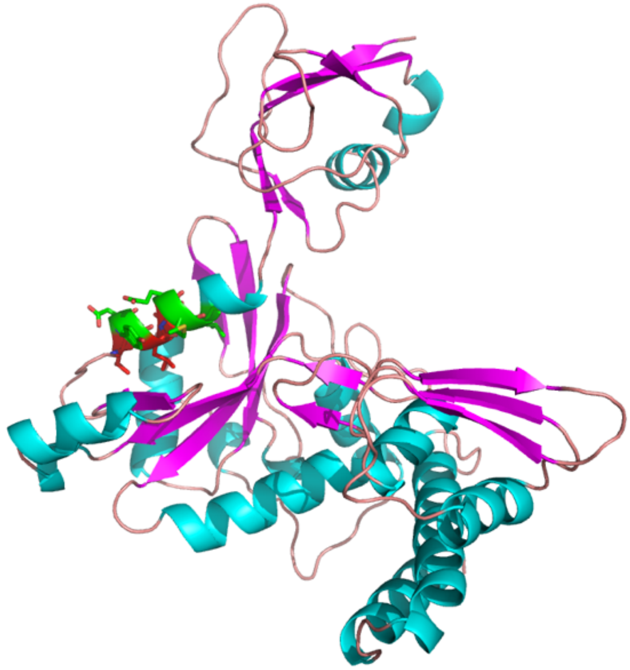

6 P29461; PTP2

Location in protein: 679-LDFLLSIL-686
Structural Information: 23.7% Sequence Identity with PDB 3oc3A and 42% identical with 2cfv in the PTP domain.

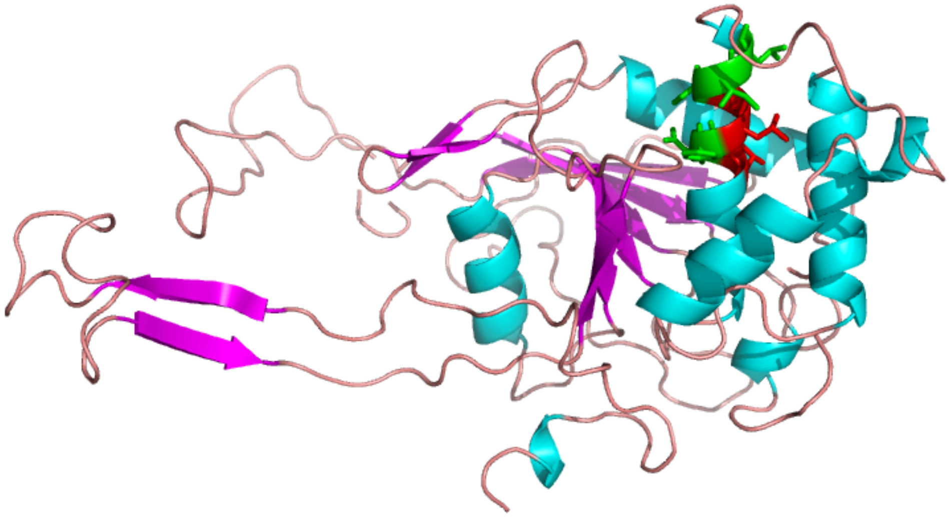

7 P36000; β-ADAPTIN

Location in protein: 409-LDILLELL-416
Structural Information: 40 % Sequence Identity to 4uqi.

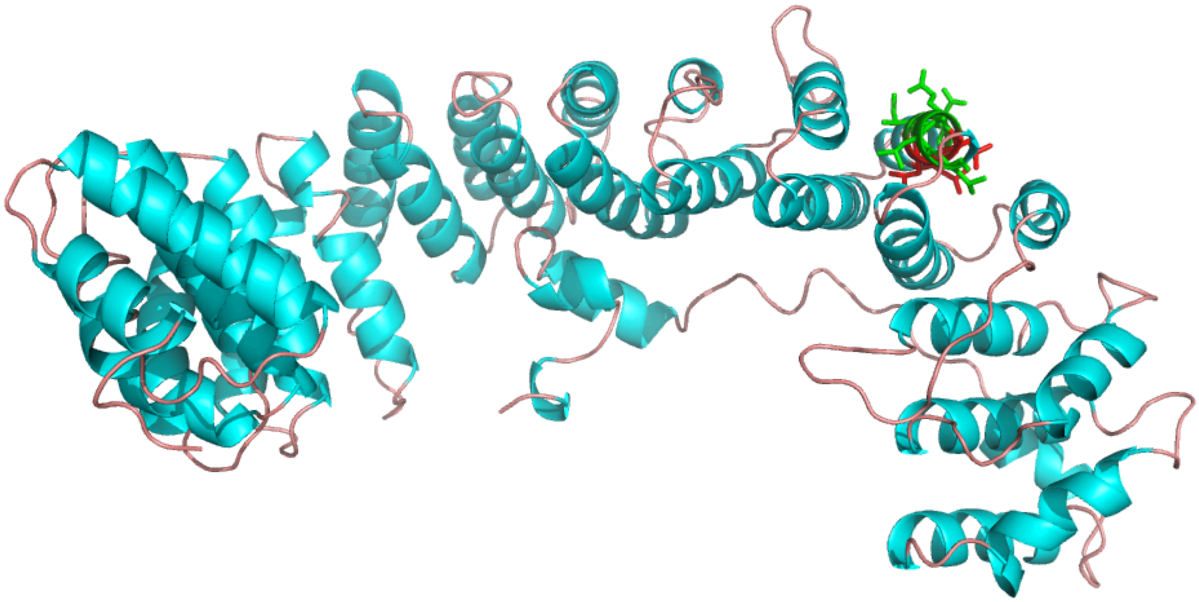

8 P40421; RDGC

Location in protein: 163-LDDLLVVL-170
Structural Information: 40 % sequence identity with PBD 5jjtA; LD motif is inaccessible in the catalytic region.

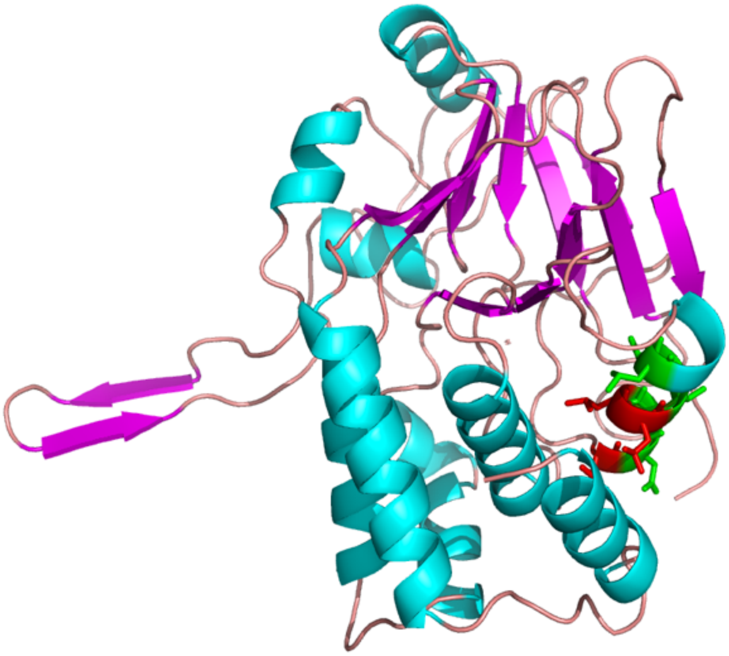

9 P38570; Integrin alpha-E; ITGAE

Location in protein: 375-LDGLLSKL-382
Structural Information: 38% sequence identity with PDB 1na4 in VWFA domain.

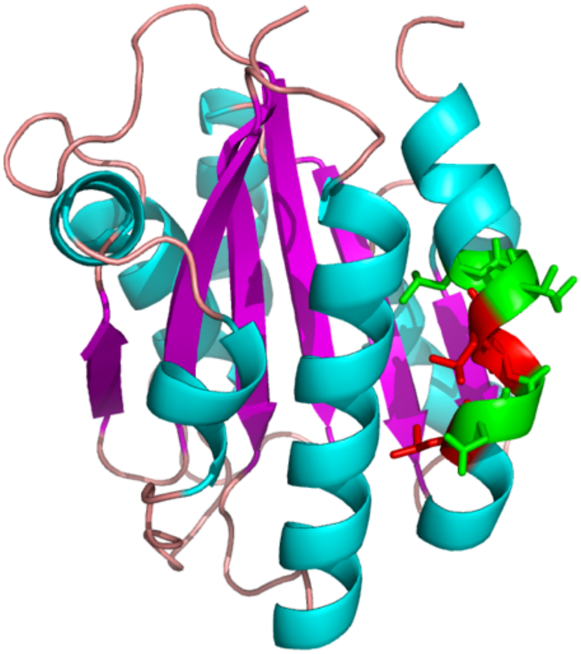

10 P52306; RAP1 GTPase DISSOCIATION STIMULATOR 1; RAP1GDS

Location in protein: 27-LDCLLQAL-34
Structural Information: 24.2% sequence identity with PDB 4hxt in ARM repeat.

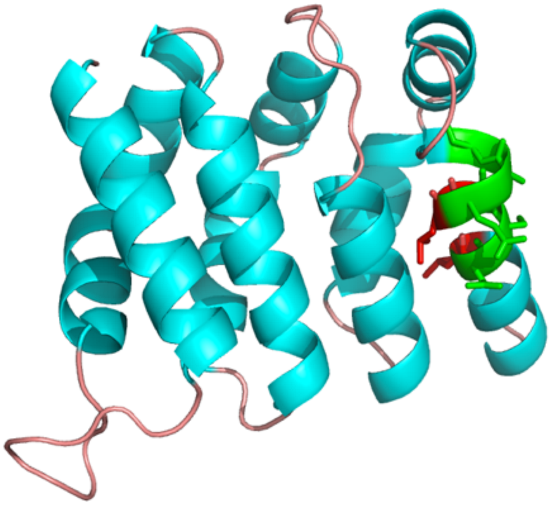

11 P53046; RHO1 GDP-GTP exchange protein 1; ROM1

Location in protein: 713-LDNMLLFL-720
Structural Information: 17.7% identical with PDB 3kz1 (pictured) or 24% identical with PH domain only of 1xcgA.

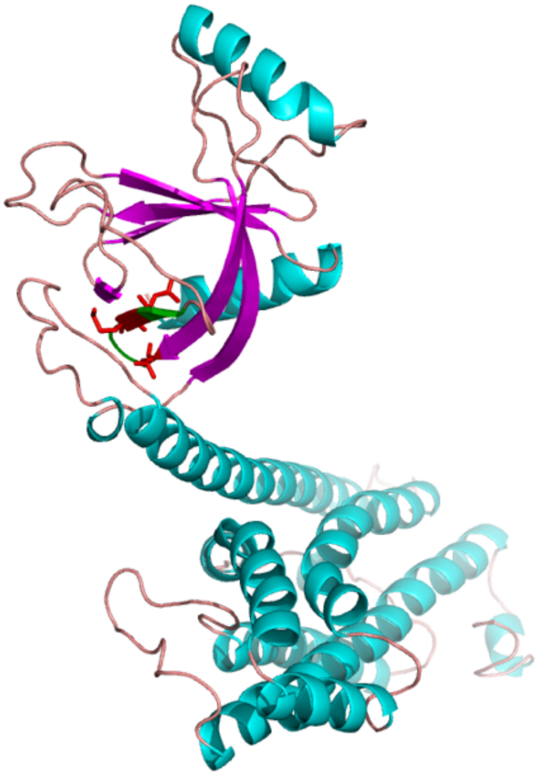

12 P35579; MYOSIN-9; MYH9

Location in protein: 1422-LDDLLVDL-1429
Structural Information: 17% sequence identity to PDB entry 2efr (tropomyosin) in C-terminal coiled-coil region.

13 P24216; HAP2

Location in protein: 443-LDVLMTS-450
Structural Information: 43 % identity with PDB 5krw for residues 385-461, but poor model quality. 13% sequence identify with PDB entry 1gk4, similar to human vimentin coil 2b fragment.

14 P54762; Ephrin type-B receptor 1;EphB1

Location in protein: 3-LDYLLLLL-10
Structural Information: No structure modelling possible for this region. The region is identified as an extracellular signaling peptide (cleaved during maturation) by Phobius (below).

```
ID   Ephrin
FT   SIGNAL        1     17
FT   REGION        1      1       N-REGION.
FT   REGION        2     12       H-REGION.
FT   REGION       13     17       C-REGION.
FT   TOPO_DOM     18    540       NON CYTOPLASMIC.
FT   TRANSMEM    541    563
FT   TOPO_DOM    564    984       CYTOPLASMIC.
//
```

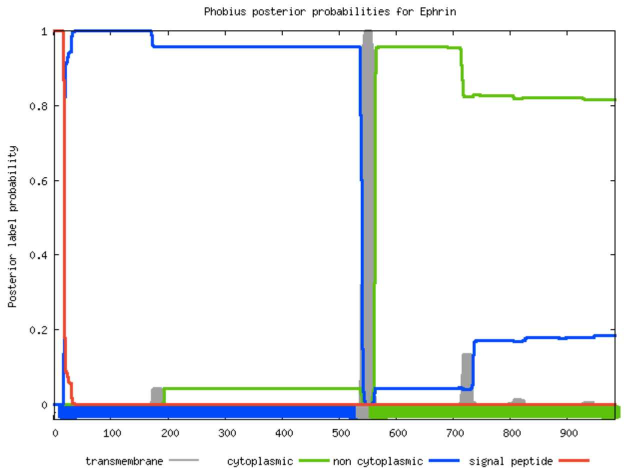

15 P38650; CYTOPLASMIC DYNEIN 1 HEAVY CHAIN 1; DYNC1H1

Location in protein: 1361-LDGLLNQL-1368
Structural Information: No homology model possible. The LD motif is found in the coiled-coil STEM region.

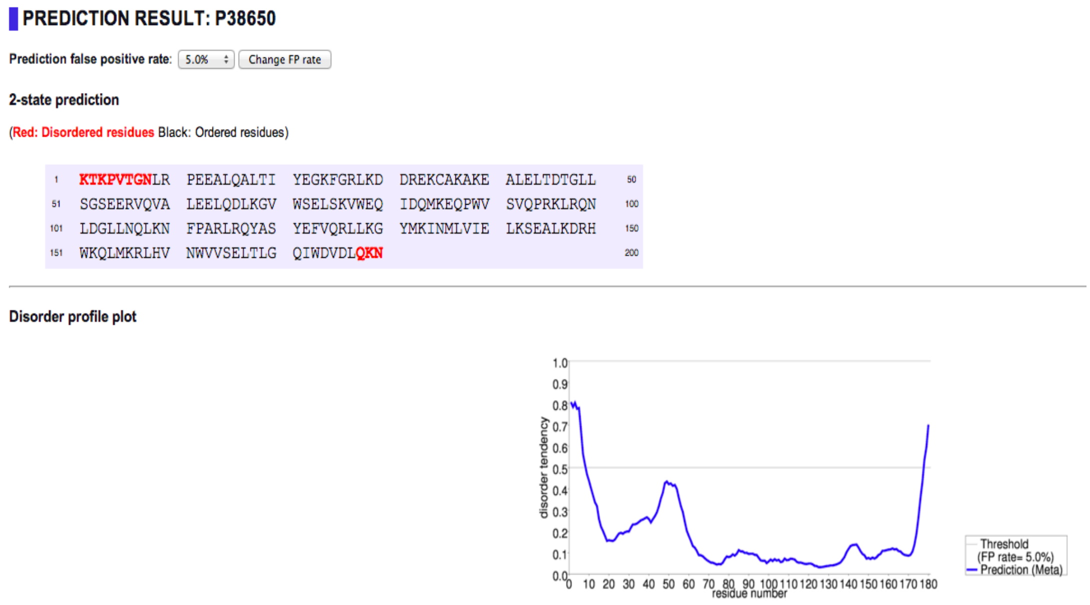

16 P51592; E3 UBIQUITIN-PROTEIN LIGASE; HYD

Location in protein: 1453-LDTLLLTL-1460
Structural Information: No homologous structure for modelling.

17 Q04205; TENSIN; TNS

Location in protein: 807-LDVLMLDL-814
Structural Information: No 3D template is available. This motif is promising, because an interaction between the homologue tensin3 and FAK and Cas has been reported (Cui et al. Mol Cancer Res. 2003). Tensin is also involved in the function of focal adhesions. The LD motif of tensin is located in a disordered region and predicted helical.

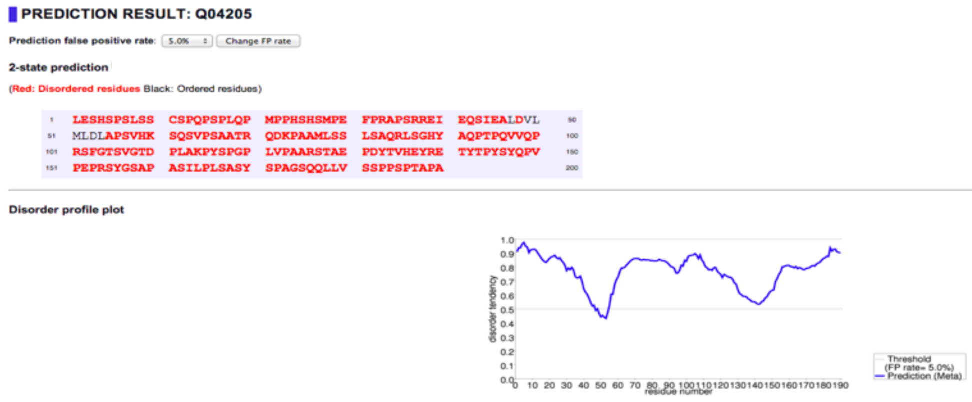

Supplementary Figure 2.2. Secondary structure predictions (SS3: three states, namely H: helix, E: beta strand, C: coil; SS8: eight states, namely H: α helix, G: 3-helix, I: 5-helix, E: extended β ladder, B: β bridge, T: hydrogen bonded turn, S: bend, L: loop), solvent accessibility (ACC; B: buried; M: medium exposed, E: solvent exposed) and disorder (DISO: order [.] and disorder [*]) for the non-paxillin motifs suggested by SlimSearch4 (Krystkowiak and Davey, 2017), which was the only algorithm which predicted a reasonable number of LD motif candidate in the human proteome (see **Supplementary Table 1**). The feature predictions were established by the RaptorX server (Kallberg, et al., 2014). The suggested LD motif region is boxed. Amino acid are numbered starting with 20 positions upstream of the LD motif (unless the LD motif is situated at the N-terminus, which is then taken as number 1).

According to this analysis, 27/34 of the suggested sequences appear to have secondary structure or order/disorder features unfitting for known LD motifs. Of the remaining ones, 4/7 lack the typical amino acid features, in particular the presence of additional acidic charges (GAPD1, F16B1, TENC1, CK072). Hence, only 3/34 motifs would remain as plausible candidates (MIAP, SRTD1, AZI1).

1. E5RHQ5|NPB11_HUMAN

	1	11	21	31	41
SEQ	PPPPTQQHCI	TDNSLSLKTP	LECLLTPLPP	SADDNLKTPP	ECLLTPLP
SS3	CCCCCCCCCC	CCCCCCCCCC	CCCCCCCCCC	CCCCCCCCCC	CCCCCCCC
SS8	LLLLLLLLLL	LLLLLLLLLL	LGLSLLLLLL	LLLTLLLSLL	LLSLLLLL
ACC	EEEEEEEEEM	EEEEMEMEME	MEMMMMEMEE	EEEEEMEEME	EMEEEEME
DISO	**********	**********	**********	**********	********

2. O43166|SI1L1_HUMAN

	1	11	21	31	41
SEQ	MKPYSSSKDS	SPTLASKVDQ	LEGMLKMLRE	DLKKEKEDKA	HLQAEVQH
SS3	CCCCCCCCCC	CCCHHHHHHH	HHHHHHHHHH	HHHHHHHHHH	HHHHHHCC
SS8	LLLLLLLLLL	LLLHHHHHHH	HHHHHHHHHH	HHHHHHHHHH	HHHHHHLL
ACC	EEEEEEEEEE	EEEEEEEEEE	EEEEBEEEEE	EEEEEEEEEE	EEEEEEEE
DISO	**********	**********	**********	**********	********

3. O60941|DTNB_HUMAN

	1	11	21	31	41
SEQ	DELEQRMSAL	QESRRELMVQ	LEELMKLLKE	EEQKQAAQAT	GSPHTSPT
SS3	CHHHHHHHHH	HHHHHHHHHH	HHHHHHHHHH	HHHHHHHCCC	CCCCCCCC
SS8	LHHHHHHHHH	HHHHHHHHHH	HHHHHHHHHH	HHHHHHHLLL	LLLLLLLL
ACC	EEEEEEEEEE	EEEEEEEEEE	EEEEEEEEEE	EEEEEEEEEE	EEEEEEEE
DISO	**********	**********	**********	**********	********

4. O75069|TMCC2_HUMAN

	1	11	21	31	41
SEQ	GPGGALGSPK	SNALYGAPGN	LDALLEELRE	IKEGQSHLED	SMEDLKTQ
SS3	CCCCCCCCCC	CCCCCCCCCC	HHHHHHHHHH	HHHHHHHHHH	HHHHHHCC
SS8	LLLLLLLLLL	LLLLLLLLLL	HHHHHHHHHH	HHHHHHHHHH	HHHHHHLL
ACC	EEEEEEEEEE	EEEEEEEEEE	EEEMMEEMEE	EEEEEEEEEE	EEEEEEEE
DISO	**********	**********	**********	**********	********

5. P12270|TPR_HUMAN

	1	11	21	31	41
SEQ	SRLEEQMNGL	KTSNEHLQKH	VEDLLTKLKE	AKEQQASMEE	KFHNELNA
SS3	CHHHHHHHHH	HHHHHHHHHH	HHHHHHHHHH	HHHHHHHHHH	HHHHHHCC
SS8	LHHHHHHHHH	HHHHHHHHHH	HHHHHHHHHH	HHHHHHHHHH	HHHHHHLL
ACC	EEEEEEEEEE	EEEEEEEEEE	EEEEEEEEEE	EEEEEEEEEE	EEEEEEEE
DISO	....	....	....	....	......**

6. P31949|S10AB_HUMAN

	1	11	21	31	41
SEQ	LSFMNTELAA	FTKNQKDPGV	LDRMMKKLDT	NSDGQLDFSE	FLNLIGGL
SS3	CCCCHHHHHH	HHCCCCCHHH	HHHHHHHHCC	CCCCCCCHHH	HHHHHCCC
SS8	LLLLHHHHHH	HHHSLLLHHH	HHHHHHHHLT	TLSSLELHHH	HHHHHTTL
ACC	EEMMMEMBME	MBEEEEEEEM	BEEBBEEMME	EEEEEBMBEM	BMEMBEEE
DISO	**........	....	....	....	.......*

7. Q14602|ID2B_HUMAN

	1	11	21
SEQ	SIRKNSLLDH	RLGISQSKTP	VDDLMSLL
SS3	CCCCCCCCCC	CCCCCCCCCC	CCCCCCCC
SS8	LLLLLLLLLL	LLLLLLLLLL	LLLLLLLL
ACC	EEEEEEEEEE	EEEEEEEEEE	EEEEEEEE
DISO	**********	**********	********

8. Q14C86|GAPD1_HUMAN

	1	11	21	31	41
SEQ	LVNFMKSVMS	GDQLREDRMA	LDNLLANLPP	AKPGKSSSLE	MTPYNTPQ
SS3	CCCCEECCCC	CCCCHHHHHH	HHHHHHCCCC	CCCCCCCCCC	CCCCCCCC
SS8	LLLEEEELLL	LLLLHHHHHH	HHHHHHTSLL	LLLLLLLLLL	LLLLLLLL
ACC	EEEEEEEEEE	EEEEEEMMEM	BEEBMEEMEE	EEEEEEEEEE	EEEEEEEE
DISO	**********	**********	**********	**********	********

9. Q16760|DGKD_HUMAN

	1	11	21	31	41
SEQ	TESSEESEVM	AKKCSVLKEK	LDSLLKTLDD	ESQASSSLPN	PPPTIAEE
SS3	CCCHHHHHHH	HHHHHHHHHH	HHHHHHHHHH	HHHHCCCCCC	CCCCCCCC
SS8	LLLHHHHHHH	HHHHHHHHHH	HHHHHHHHHH	HHHHLLLLLL	LLLLLLLL
ACC	EEEEEEBEEM	EEEBEEEEEE	BMEBMEEEEE	EEEEEEEEEE	EEEEEEEE
DISO	****......	....	....******	**********	********

10. Q5W0V3|F16B1_HUMAN

	1	11	21	31	41
SEQ	YYIETSDDKA	PVTDTNIPSH	LEQMLDILVQ	EENERESGET	GPCMEYLL
SS3	CCCCCCCCCC	CCCCCCHHHH	HHHHHHHHHH	HHHHCCCCCC	CCCHHCCC
SS8	LLLLLLLLLL	LLLLLLHHHH	HHHHHHHHHH	HHHHHLLLLL	LLHHHHHL
ACC	EEEEEEEEEE	EEEEMEBMEM	BMMBBEMBME	EEEEEEEEEE	EEEEEEEE
DISO	**********	******....	....	.*********	********

11. Q63HR2|TENC1_HUMAN

	1	11	21	31
SEQ	MKSSGPVERL	LRALGRRDSS	RAASRPRKAE	PHSF
SS3	CCCCCHHHHH	HHHHCCCCCH	HHHCCCCCCC	CCCC
SS8	LLLLLHHHHH	HHHHLLLLLH	HHHLLLLLLL	LLLL
ACC	EEEEMEBMEB	MEEMEEEEEE	EEEEEEEEEE	EEEE
DISO	**********	**********	**********	****

12. Q7Z3Z2|RD3_HUMAN

	1	11	21	31	41
SEQ	WLRWNEAPSR	LSTRSPAEMV	LETLMMELTG	QMREAERQQR	ERSNAVRK
SS3	CCCCCCCCCC	CCCCCHHHHH	HHHHHHHHHH	HHHHHHHHHH	HHHHHHCC
SS8	LLLLLLLLLL	LLLLLHHHHH	HHHHHHHHHH	HHHHHHHHHH	HHHHHHLL
ACC	EEEMEEEEEE	EEEEEEEEEE	MEEEEEEEEE	EEEEEEEEEE	EEEEEEEE
DISO	**********	*******...	.........*	**********	********

13. Q8IV76|PASD1_HUMAN

	1	11	21	31	41
SEQ	EQLEERTWLL	HDAIQNQQNA	LELMMDHLQK	QPNTLRHVVI	PDLQSSEA
SS3	CHHHHHHHHH	HHHHHHHHHH	HHHHHHHHHC	CCCCCCCCCC	CCCCCCCC
SS8	LHHHHHHHHH	HHHHHHHHHH	HHHHHHHHHT	SLLLLLLLLL	LLLLLLLL
ACC	EEEEEEEEEE	EEEEEEEEEE	EEEEMEEBEE	EEEEEEEEEE	EEEEEEEE
DISO	*.........	....	....	.*********	********

14. Q8IWP9|CC28A_HUMAN

	1	11	21	31	41
SEQ	LYGELEELPE	DKRKTASDSN	LDRLLSDLEE	LNSSIQKLHL	ADAQDVPN
SS3	CCCCCCCCCC	HHHHHHHHHH	HHHHHHHHHH	HHHHHHHHHH	CCCCCCCC
SS8	LLLLLLLLLH	HHHHHHHHHH	HHHHHHHHHH	HHHHHHHHHH	HLLLLLLL
ACC	EEEEEEEEEE	EEEEEEEEEE	EEEEEEEEEE	EEEEEEEEEE	EEEEEEEE
DISO	**********	**********	**********	**********	********

15. Q8N3J3|CQ053_HUMAN

	1	11	21	31
SEQ	TAQNLEAEAS	PEEELPEADD	LDGLLSELPE	DFFCGTSS
SS3	CCCCCCCCCC	CCCCCCCCCC	CCCHCCCCCC	CCCCCCCC
SS8	LLLLLLLLLL	LLLLLLLLLL	LLLHHLLLLL	LLLLLLLL
ACC	EEEEEEEEEE	EEEEEEEEEE	EEEEEEEMEE	EEEEEEEE
DISO	**********	**********	**********	********

16. Q8NBR9|CK072_HUMAN

	1	11	21	31	41
SEQ	QLPELGLRSP	NNKSPTGPHP	LEHLLARLLK	RRRRSTLMSS	PRSLLCSI
SS3	CCCCCCCCCC	CCCCCCCCCH	HHHHHHHHHH	HHHHHCCCCC	CCCCCCCC
SS8	LLLLLLLLLL	LLLLLLLLLH	HHHHHHHHHH	HHHHHHHLLL	LLLLLLLL
ACC	EEEEEEEEEE	EEEEEEEEEE	BEEBBEEBME	EMEEEEEEEE	EEEEEEEE
DISO	**********	**********	**********	**********	********

17. Q8NDD1|CA131_HUMAN

	1	11	21	31	41
SEQ	PTMSQEQGPG	SSTPPSSPTL	LDALLQNLYD	FGGTEGETEQ	KKIIKKRE
SS3	CCCCCCCCCC	CCCCCCCHHH	HHHHHHHHCC	CCCCCCHHHH	HHHHHHCC
SS8	LLLLLLLLLL	LLLLLLLHHH	HHHHHHHHHL	TTLLLLHHHH	HHHHHHLL
ACC	EEEEEEEEEE	EEEEEEMEEE	BMEBBEEBME	EEEEEEEEEE	EEEEEEEE
DISO	**********	**********	**********	**********	********

18. Q8TC57|M1AP_HUMAN

	1	11	21	31	41
SEQ	MLPSTFPLLP	EDPHDDSLKN	VESMLDSLEL	EPTYNPLHVQ	SHLYSHLS
SS3	CCCCCCCCCC	CCCCHHHHHH	HHHHHHCCCC	CCCCCCCCCC	CCCCCCCC
SS8	LLLLLLLLLL	LLLLHHHHHH	HHHHHHHLLL	LLLLLLLLLL	LLGGGGLL
ACC	EEEEEMEEEE	EEMEEEMMEE	BEEMBEEMEE	EEEMEEBMBE	MMBMEEME
DISO	**********	**********	**********	**********	********

19. Q8WZA0|LZIC_HUMAN

	1	11	21	31	41
SEQ	MASRGKTETS	KLKQNLEEQL	DRLMQQLQDL	EECREELDTD	EYEETKK
SS3	CCCCCCHHHH	HHHHHHHHHH	HHHHHHHHHH	HHHHHHCCHH	HHHHHCC
SS8	LLLLLLHHHH	HHHHHHHHHH	HHHHHHHHHH	HHHHHHLLHH	HHHHHHL
ACC	EEEEEEEEEE	EEEEEEEEEE	EEEEEEEEEE	EEEEEEEEEE	EEEEEEE
DISO	**********	*.........	....	...*******	*******

20. Q92542|NICA_HUMAN

	1	11	21	31	41
SEQ	LELWMHTDPV	SQKNESVRNQ	VEDLLATLEK	SGAGVPAVIL	RRPNQSQP
SS3	CCCCCCCCCC	CCCCHHHHHH	HHHHHHHHHH	HCCCCCCEEE	CCCCCCCC
SS8	LLLEELLLLL	LLHHHHHHHH	HHHHHHHHHH	HLLLLLLEEE	LLLLLLLL
ACC	EEMMBMEEEE	EEEEEEBMEM	BMEBBEMBEE	EEEEMEEBMM	EEEEEEEE
DISO	**********	**********	**********	**********	********

21. Q92859|NEO1_HUMAN

	1	11	21	31
SEQ	MLEDSESSYE	PDELTKEMAH	LEGLMKDLNA	ITTA
SS3	CCCCCCCCCC	HHHHHHHHHH	HHHHHHHHHH	HHCC
SS8	LLLLLLLLLL	HHHHHHHHHH	HHHHHHHHHH	HHLL
ACC	EEEEEEEEEE	EEEEEEEEEE	EEEEEEEEEE	EEEE
DISO	**********	**********	**********	****

22. Q96FS4|SIPA1_HUMAN

	1	11	21	31	41
SEQ	GTPKSDAEPE	PGNLSEKVSH	LESMLRKLQE	DLQKEKADRA	ALEEEVRS
SS3	CCCCCCCCCC	CCCHHHHHHH	HHHHHHHHHH	HHHHHHHHHH	HHHHHHCC
SS8	LLLLLLLLLL	LLLHHHHHHH	HHHHHHHHHH	HHHHHHHHHH	HHHHHHLL
ACC	EEEEEEEEEE	EEEEEEEEEE	EEEEEEEEEE	EEEEEEEEEE	EEEEEEEE
DISO	**********	**********	**********	**********	********

23. Q96GC5|RM48_HUMAN

	1	11	21
SEQ	LSVKEHTEED	FKGRFKARPE	LEELLAKLK
SS3	CCCCCCCHHH	HHCCCCCCHH	HHHHHHHCC
SS8	LLLLLLLHHH	HHHLLLLLHH	HHHHHHHLL
ACC	EEEEEMEEEM	EEEEEEEEEE	BEEMMEEME
DISO	**********	****......	....*****

24. Q9BUN5|CC28B_HUMAN

	1	11	21	31	41
SEQ	EDGVTEGLPE	EQKKTMADRN	LDQLLSNLED	LSNSIQKLHL	AENAEPEE
SS3	CCCCCCCCCH	HHHHHHHHHH	HHHHHHHHHH	HHHHHHHHHH	HHCCCCCC
SS8	LLLLLLLLLH	HHHHHHHHHH	HHHHHHHHHH	HHHHHHHHHH	HHLLLLLL
ACC	EEEEEEEEEE	EEEEEEEEEE	EEEEEEEEEE	EEEEEEEEEE	EEEEEEEE
DISO	**********	*****.....	....	.........*	********

25. Q9H3T2|SEM6C_HUMAN

	1	11	21	31	41
SEQ	RVLVRPPPPG	CPGQAVEVTT	LEELLRYLHG	PQPPRKGAEP	PAPLTSRA
SS3	CCCCCCCCCC	CCCCCEEECC	HHHHHHHHHC	CCCCCCCCCC	CCCCCCCC
SS8	LLLLLLLLLL	LLLLLEEELL	HHHHHHHHHL	LLLLLLLLLL	LLLLLLLL
ACC	EEEEEEEEEE	MEEEEEEMEM	BMMBBEMBEE	EEEEEEEEEE	EEEEEEEE
DISO	**********	**********	**********	**********	********

26. Q9NVE4|CCD87_HUMAN

	1	11	21	31	41
SEQ	LPPLLGVVTR	HPAAGHRLEE	LEKMLRNLQE	EEASGQWDPQ	PPKSFPLH
SS3	CCCCHHHHHC	CCCHHHHHHH	HHHHHHHHHH	HHHCCCCCCC	CCCCCCCC
SS8	LLLHHHHHHL	LLLHHHHHHH	HHHHHHHHHH	HHHTLLLLLL	LLLLLLLL
ACC	EEEEEEEEEE	EEEEEEEMEE	BMEMBEEBEE	EEEEEEEEEE	EEEEEEEE
DISO	**********	**********	**********	**********	********

27. Q9P1Z9|CC180_HUMAN

	1	11	21	31	41
SEQ	KRMEQHRQKH	SLESQVQEAH	LDRLLDQLRQ	QSDKETLAFH	LEKVKDYL
SS3	CHHHHHHHHH	CHHHHHHHHH	HHHHHHHHHC	CCCHHHHHHH	HHHHHHCC
SS8	LHHHHHHHHH	HHHHHHHHHH	HHHHHHHHHT	SLLHHHHHHH	HHHHHHHL
ACC	EEEEEEEEEE	EEEEEEEEEE	EEEEEEEEEE	EEEEEEBEEM	EEEBEEEE
DISO	*******...	....	....	....	.......*

28. Q9UHF0|TKNK_HUMAN

	1	11	21	31	41
SEQ	SKRDPDLYQL	LQRLFKSHSS	LEGLLKALSQ	ASTDPKESTS	PEKRDMHD
SS3	CCCCHHHHHH	HHHHHCCCCC	HHHHHHHHHH	CCCCCCCCCC	CHHHCCCC
SS8	LLLLHHHHHH	HHHHHHSLLL	HHHHHHHHHH	LLLLLLLLLL	LHHHLLLL
ACC	EEEEEEBMEM	BMEBMEEEME	MEEBBEEBEE	EEEEEEEEEE	EEEEEEEE
DISO	**********	**********	**********	**********	********

29. Q9UHV2|SRTD1_HUMAN

	1	11	21	31	41
SEQ	KPGPEDGPGK	EEAPELDEAE	LDYLMDVLVG	TQALERPPGP	GR
SS3	CCCCCCCCCC	CCCCCCCHHH	HHHHHHHHHC	CCCCCCCCCC	CC
SS8	LLLLLLLLLL	LLLLLLLHHH	HHHHHHHHHH	LLLLLLLLLL	LL
ACC	EEEEEEEEEE	EEEEEEEEEE	EEEEEEEEEE	EEEEEEEEEE	EE
DISO	**********	**********	**********	**********	**

30. Q9UKX3|MYH13_HUMAN

	1	11	21	31	41
SEQ	ETANSKCASL	EKTKQRLQGE	VEDLMRDLER	SHTACATLDK	KQRNFDKV
SS3	CHHHHHHHHH	HHHHHHHHHH	HHHHHHHHHH	HHHHHHHHHH	HHHHHHCC
SS8	LHHHHHHHHH	HHHHHHHHHH	HHHHHHHHHH	HHHHHHHHHH	HHHHHHLL
ACC	EEEEEEEEEE	EEEEEEEEEE	EEEEEEEEEE	EEEEEEEEEE	EEEEEEEE
DISO	********..	....	....	.........*	********

31. Q9UPN4|AZI1_HUMAN

	1	11	21	31	41
SEQ	AGDNLEMMAP	SRGSAKSRGP	LEELLHTLQL	LEKEPDVLPR	PRTHHRGR
SS3	CCCCCCCCCC	CCCCCCCCCC	HHHHHHHHHH	HHHCCCCCCC	CCCCCCCC
SS8	LLLLLLLLLL	LLLLLLLLLL	HHHHHHHHHH	HHHLLLLLLL	LLLLLLLL
ACC	EEEEEEEEEE	EEEEEEEEEE	BEEBBEMBEM	MEEEEEEMEE	EEEEEEEE
DISO	**********	**********	**.......*	**********	********

32. Q9Y2G9|SBNO2_HUMAN

	1	11	21	31	41
SEQ	AQADPAALAH	QGCDINFKEV	LEDMLRSLHA	GPPSEGALGE	GAGAGGAA
SS3	CCCCHHHHHC	CCCCCCHHHH	HHHHHHHHHC	CCCCCCCCCC	CCCCCCCC
SS8	LLLHHHHHHH	TTLLLLHHHH	HHHHHHHHHL	LLLLLLLLLL	LLLLLLLL
ACC	EEEEEEEEEE	EEMEMEBEEB	BMEBBEEBEE	EEEEEEEEEE	EEEEEEEE
DISO	**********	**********	**********	**********	********

33. Q9Y4J8|DTNA_HUMAN

	1	11	21	31	41
SEQ	DELEQRMSAL	QESRRELMVQ	LEGLMKLLKT	QGAGSPRSSP	SHTISRPI
SS3	CHHHHHHHHH	HHHHHHHHHH	HHHHHHHHHH	CCCCCCCCCC	CCCCCCCC
SS8	LHHHHHHHHH	HHHHHHHHHH	HHHHHHHHHH	LLLLLLLLLL	LLLLLLLL
ACC	EEEEEEEEEE	EEEEEEEEEE	EEEEMEEEEE	EEEEEEEEEE	EEEEEEEE
DISO	**********	**********	**********	**********	********

34. Q6ZRS2|SRCAP_HUMAN

	1	11	21	31	41
SEQ	NDAEAQRREI	ELLRREGELP	LEELLRSLPP	QLLEGPSSPS	QTPSSHDS
SS3	CCHHHHHHHH	HHHHHHCCCC	HHHHHHHCCC	CCCCCCCCCC	CCCCCCCC
SS8	LLHHHHHHHH	HHHHHHTLSL	HHHHHHHSLL	LLLLLLLLLL	LLLLLLLL
ACC	EEEEEEEEEE	EEEEEEEEME	MEEBBEEMEE	EEMEEEEEEE	EEEEEEEE
DISO	**********	**********	**********	**********	********

Supplementary Figure 3. Binding Assays

Binding assays of known LD motifs and LD motifs proposed by LDMF-proposed to FAT, α-parvin and GIT1. ACA: anisotropy competition assay; DA: direct fluorescence anisotropy; MST: microscale thermophoresis; DSF: differential scanning fluorimetry.

Supplementary Figure 3.1: Binding of LD motif controls to FAT

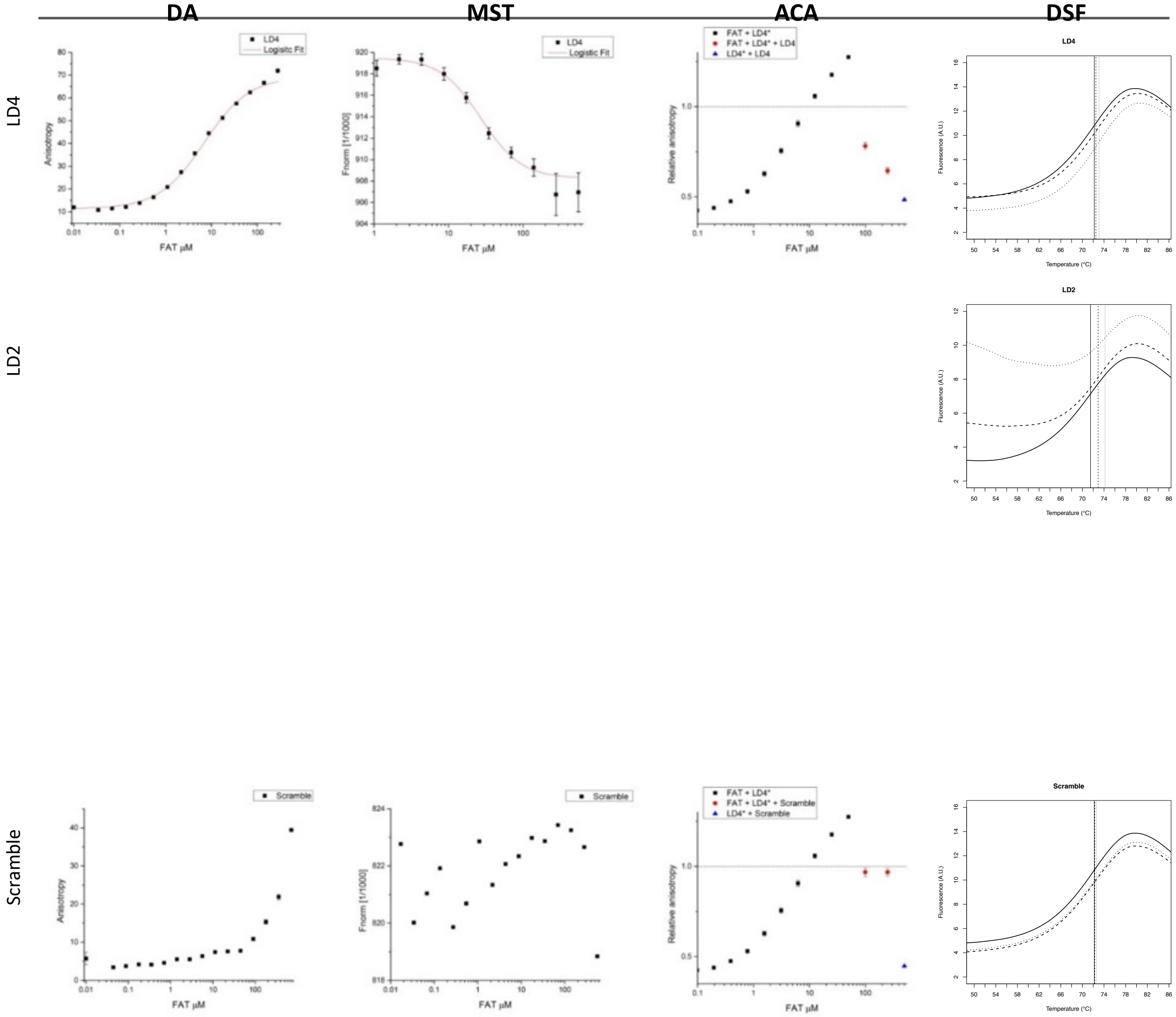

Supplementary Figure 3.2: Binding of highly likely LD motifs to FAT

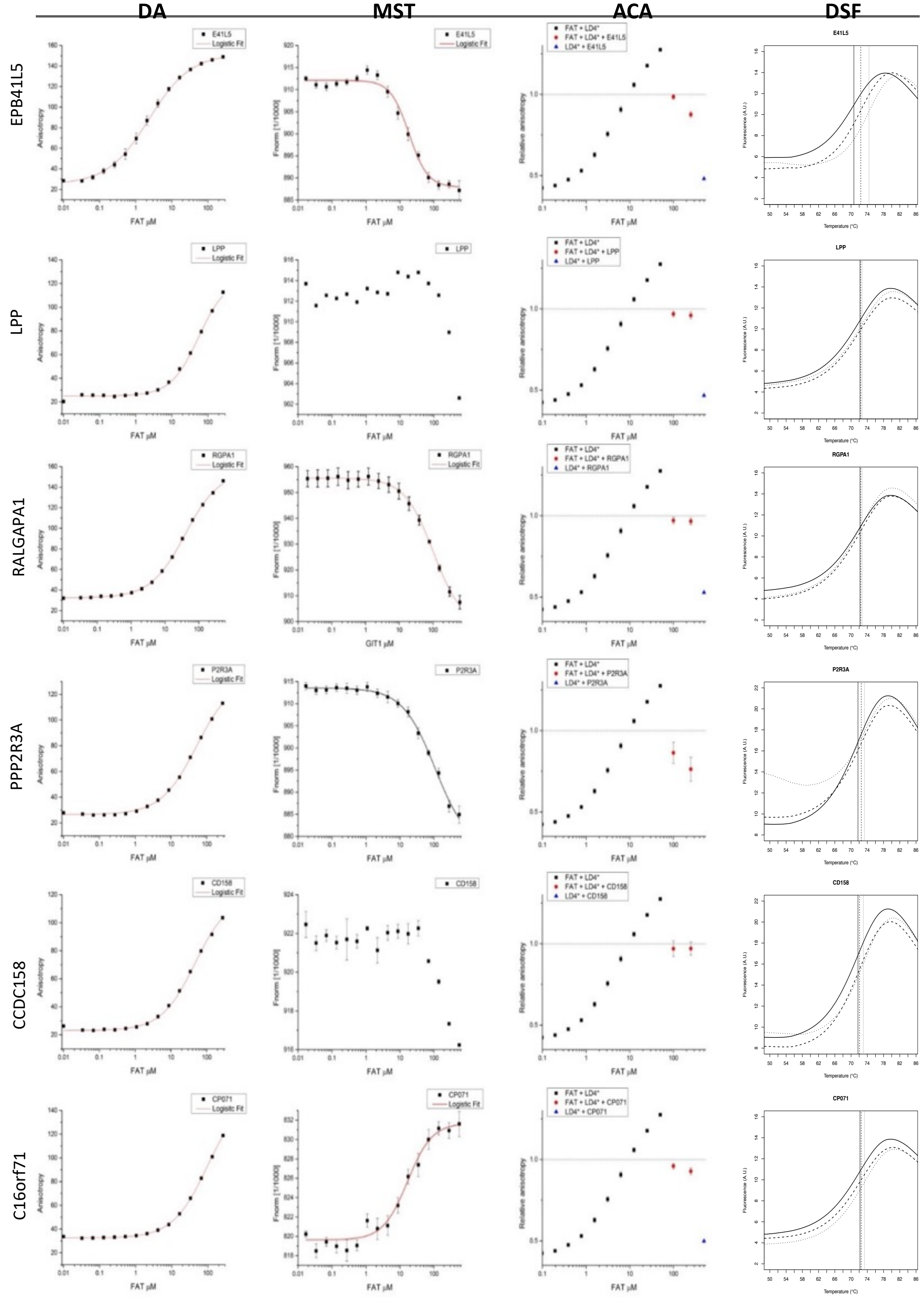

Supplementary Figure 3.3: Binding of less likely LD motifs to FAT

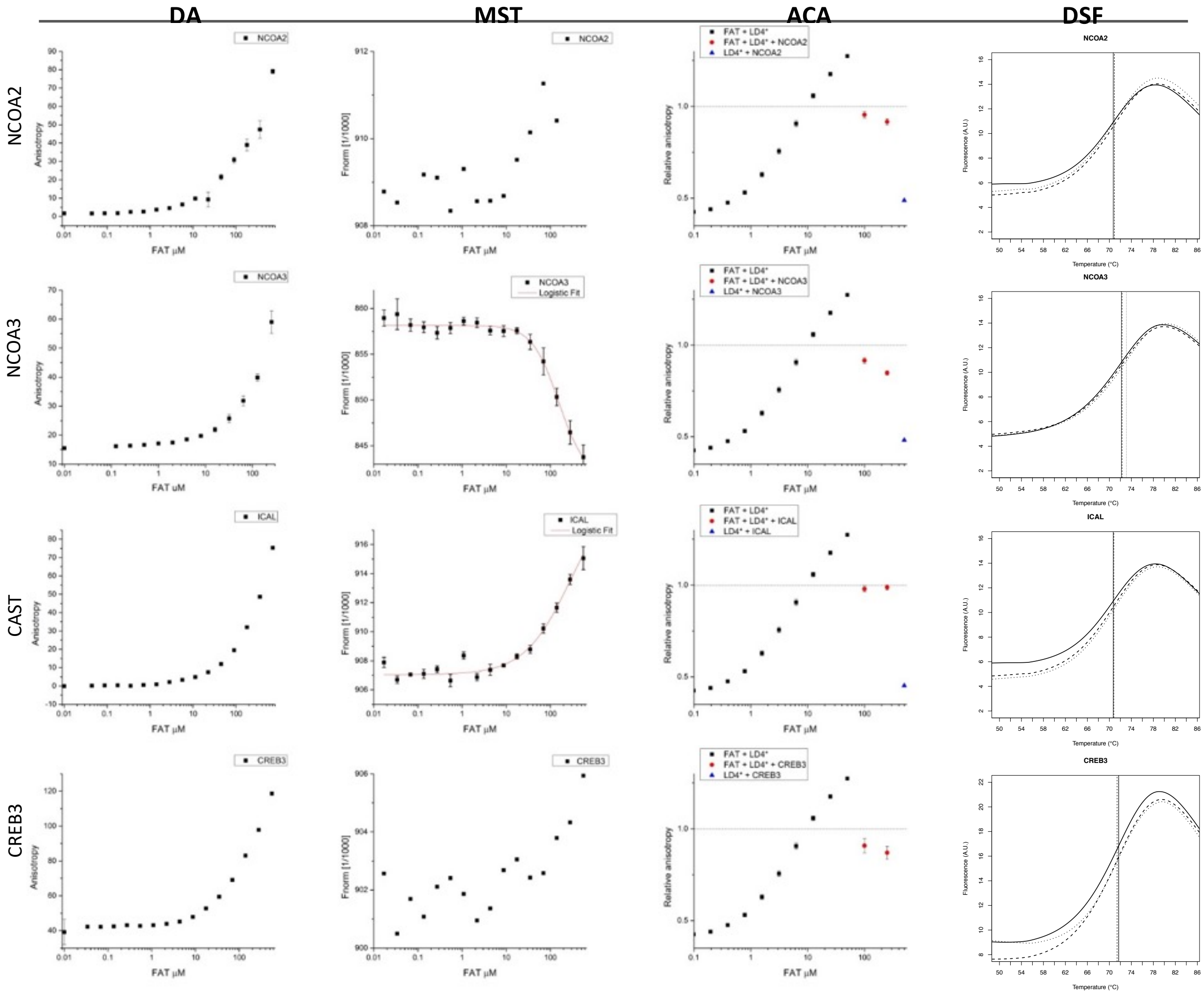

Supplementary Figure 3.4: Binding of least likely LD motifs to FAT and motifs discarded in round 1.

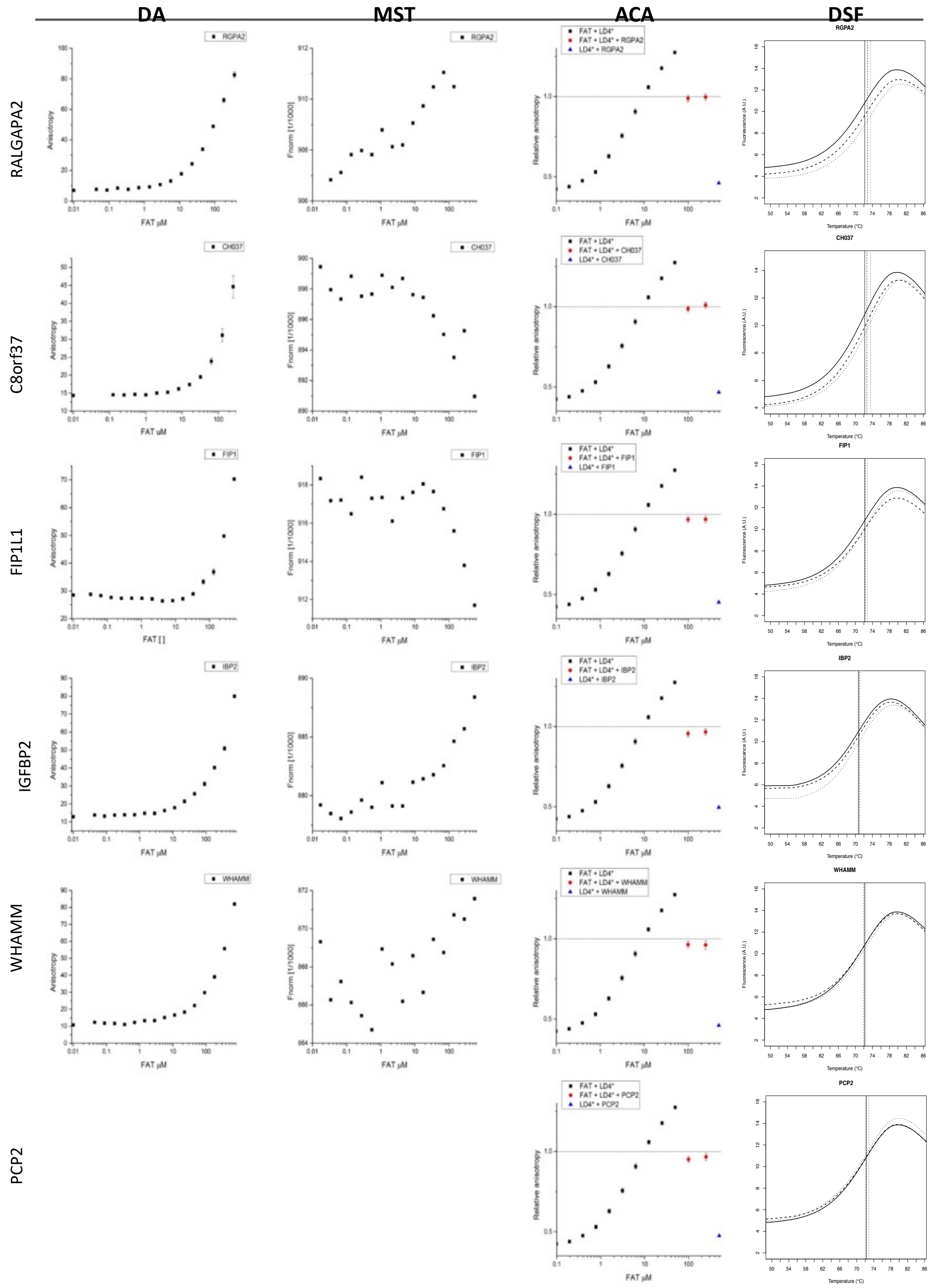

Supplementary Figure 3.5: Binding of LD motif controls to α-parvin

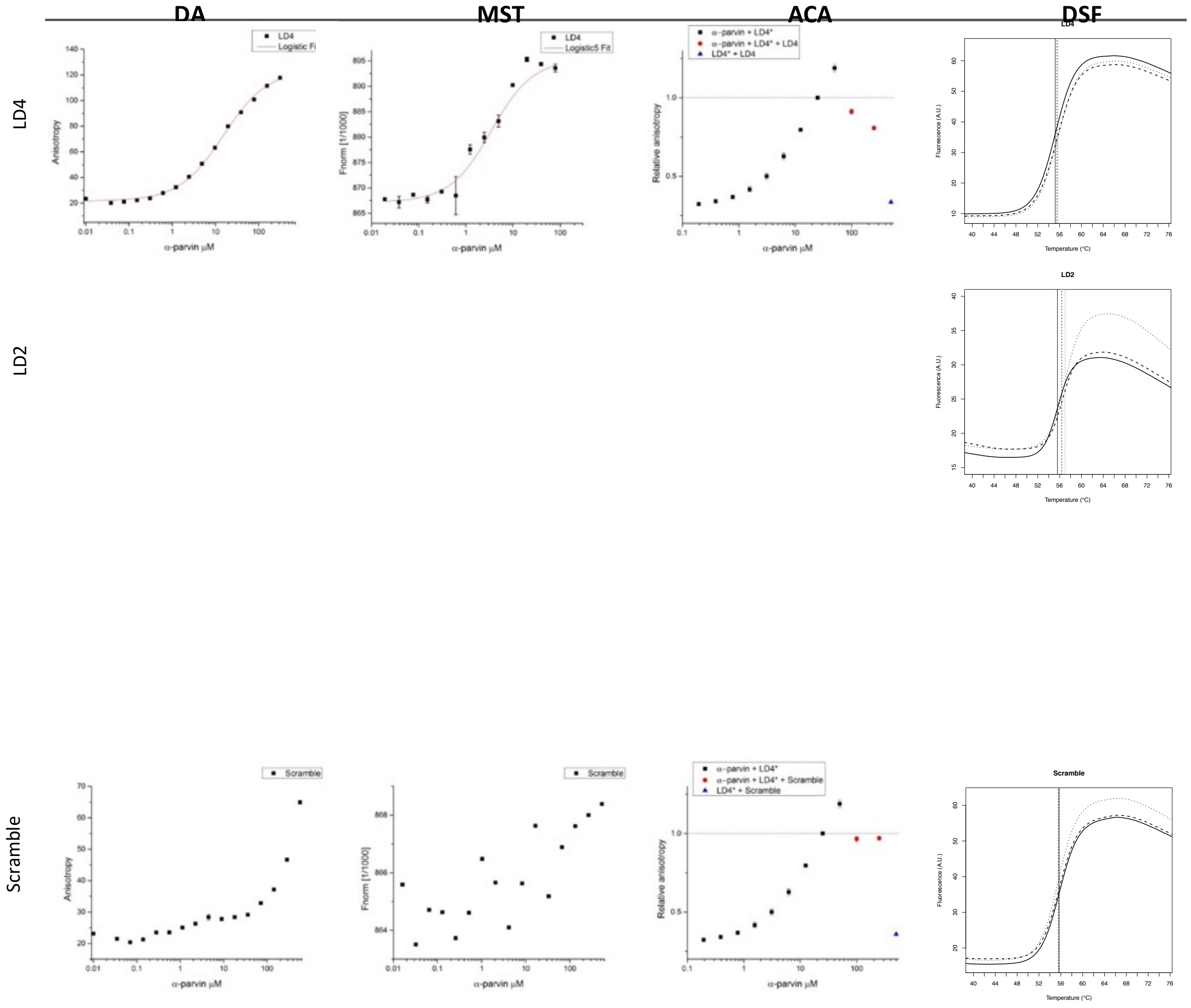

Supplementary Figure 3.6: Binding of highly likely LD motifs to α-parvin

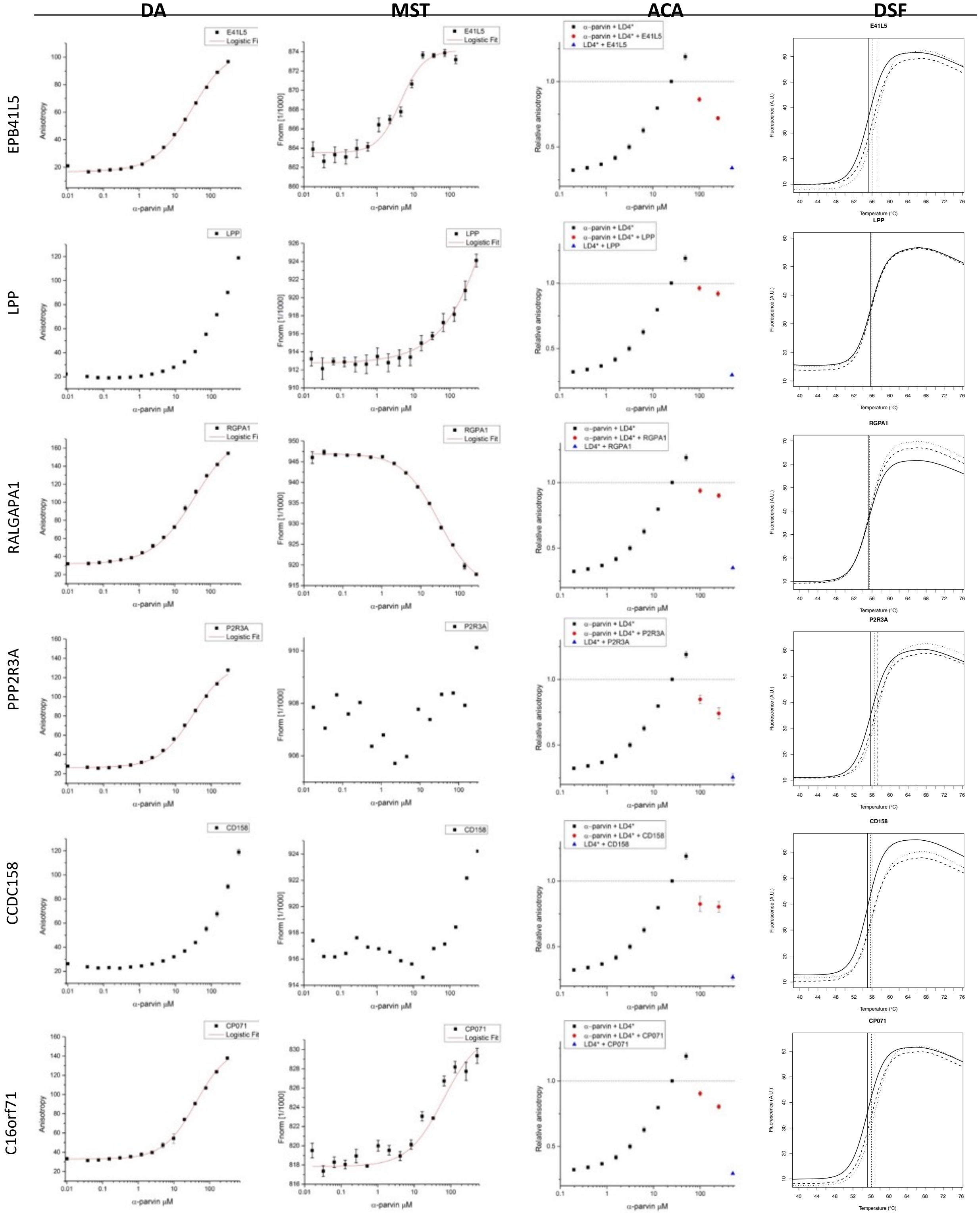

Supplementary Figure 3.7: Binding of less likely LD motifs to α-parvin

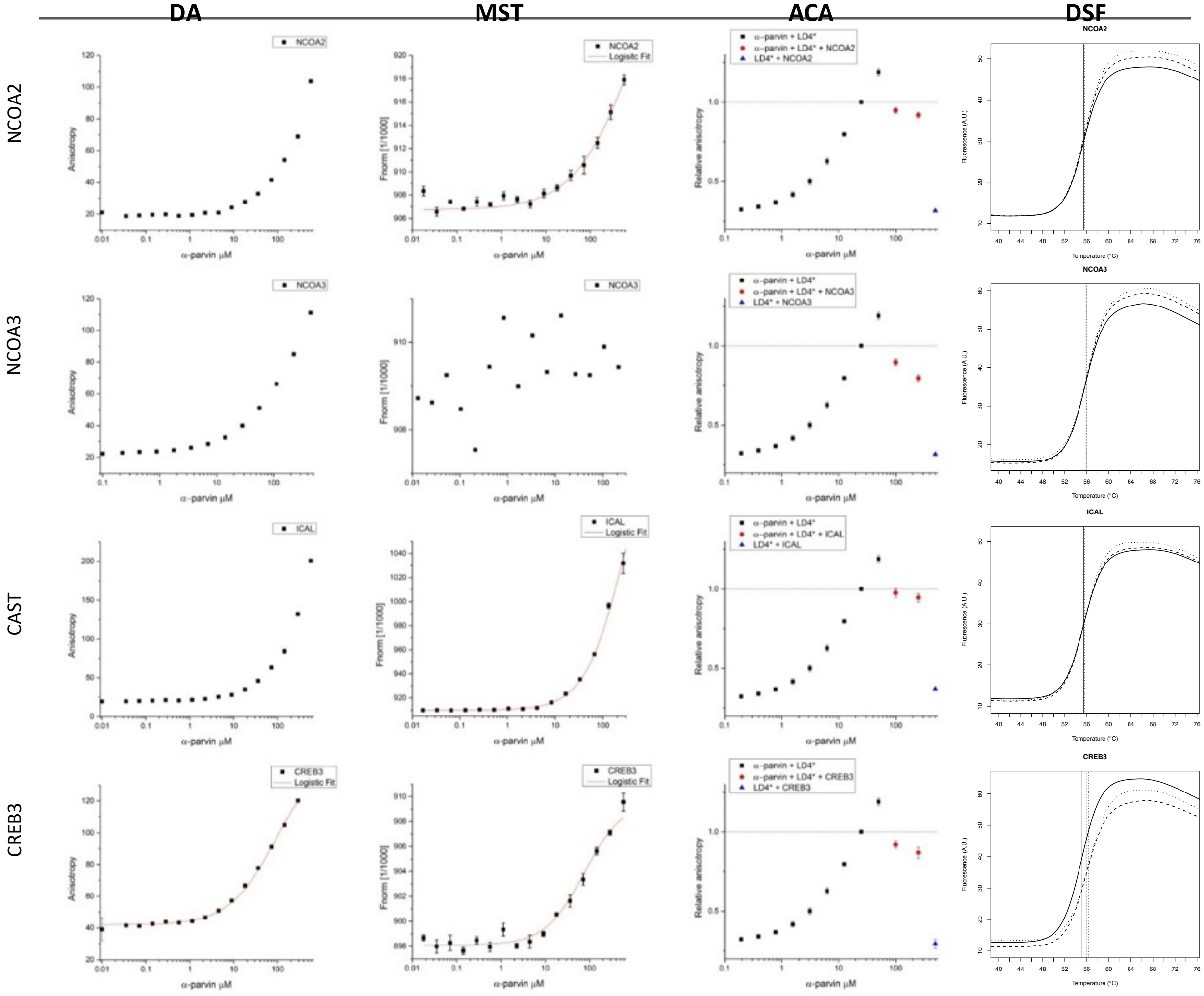

Supplementary Figure 3.8: Binding of least likely LD motifs to α-parvin and motifs discarded in round 1

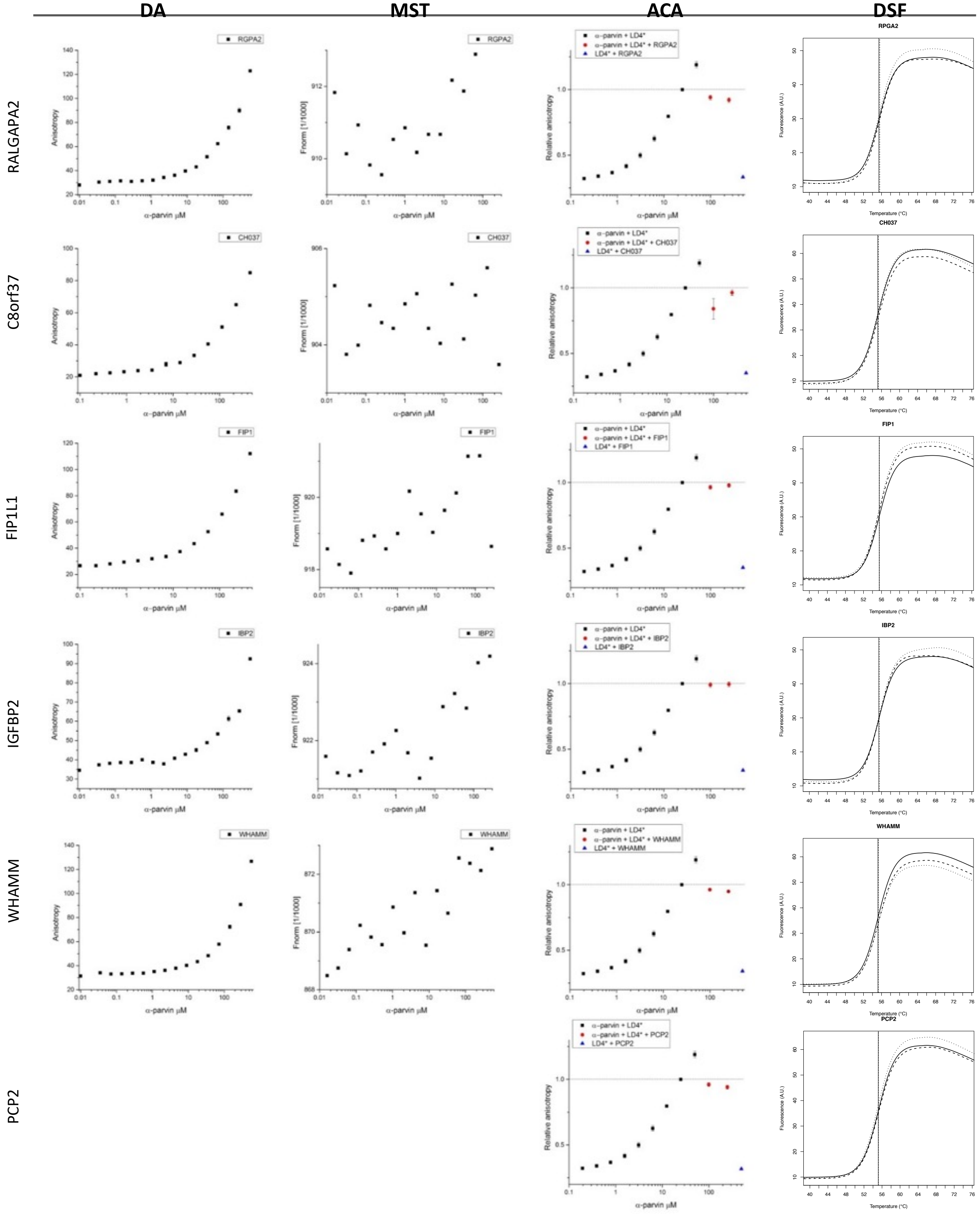

Supplementary Figure 3.9: Binding of LD motif candidates to GIT1.

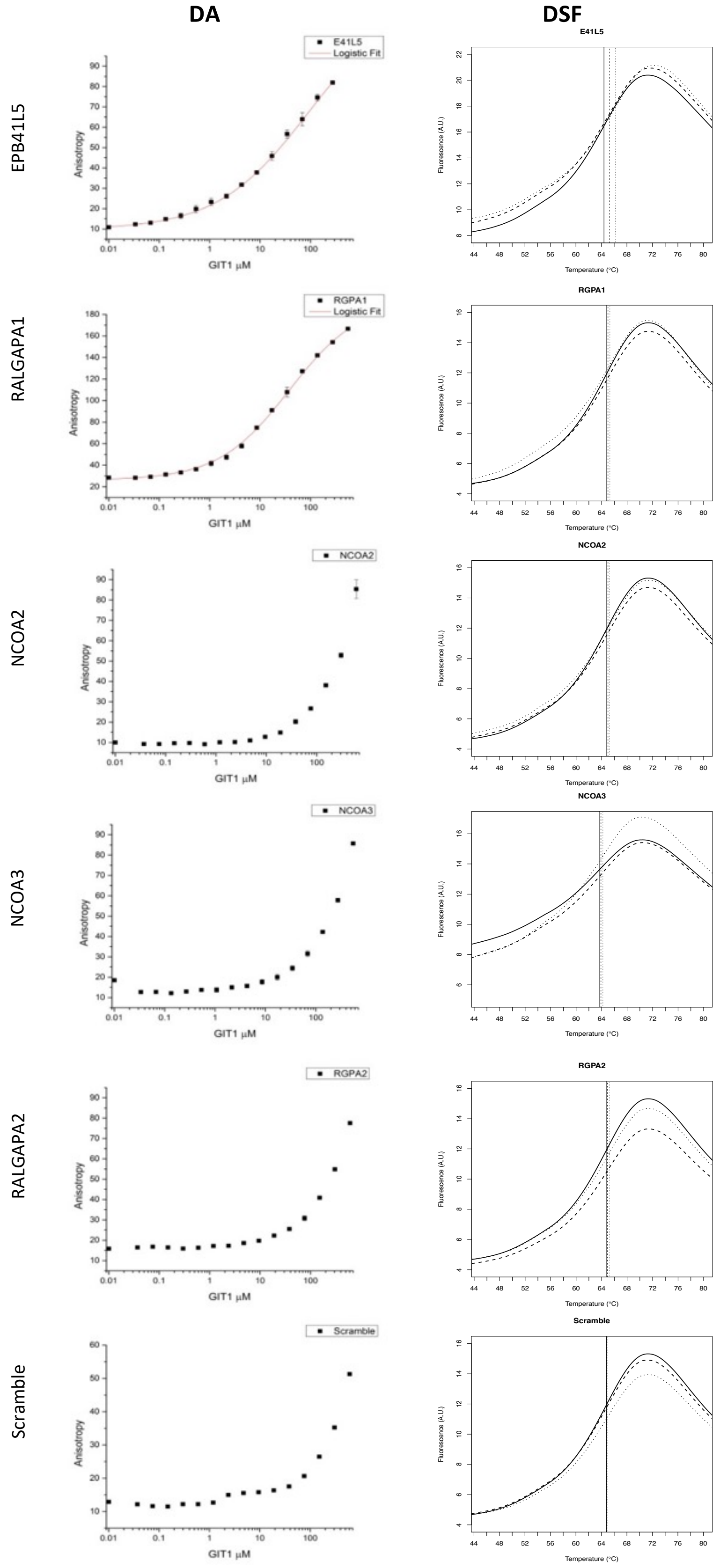

Supplementary Figure 3.10. Differential Scanning Fluorimetry (DSF)

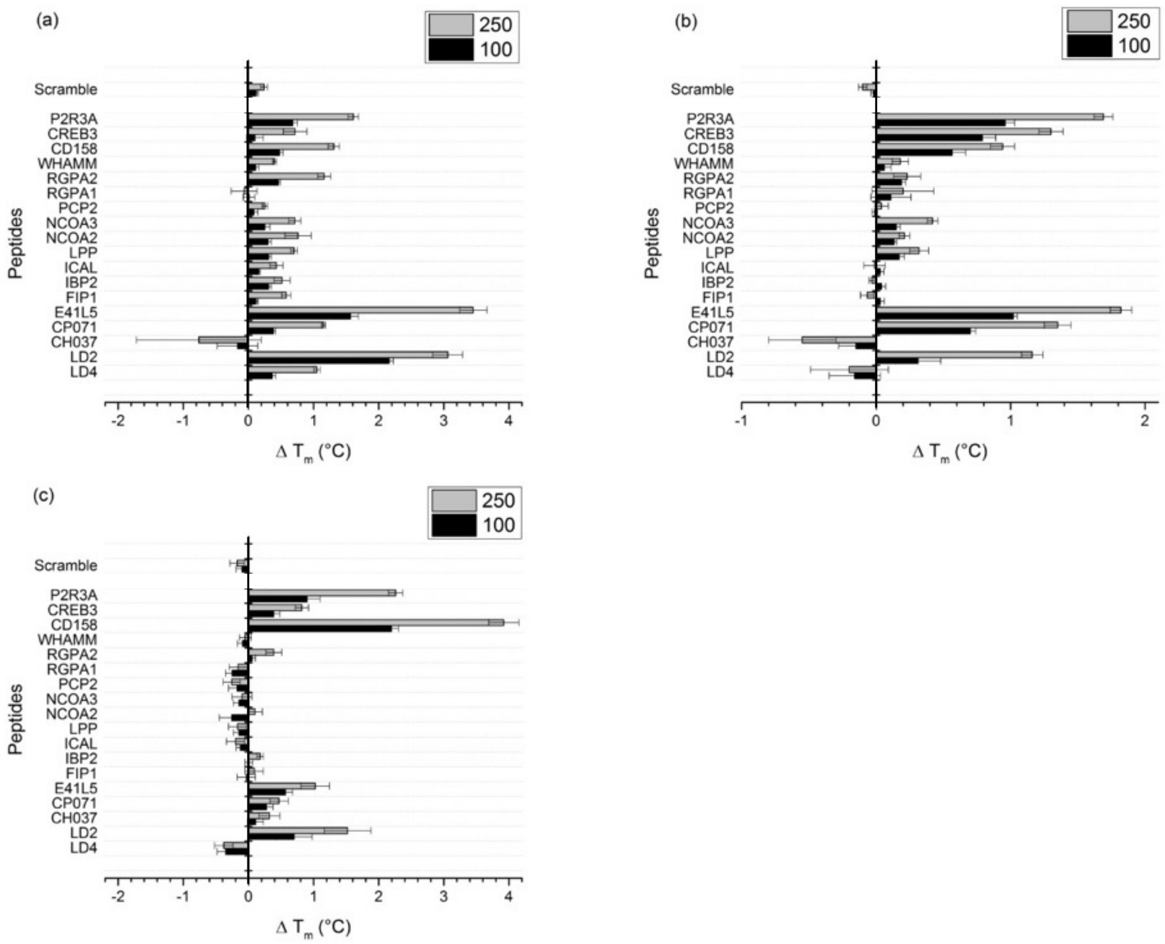

Tm shift in °C for differential scanning fluorimetry for peptides with (a) FAT (b) α-parvin and (c) GIT1. The Uniprot identifiers are given instead of protein gene names. Genes were both identifiers differ are P2R3A: PPP2R3A; CD158:CCDC158; RGPA1/2: RALGAPA1/2; ICAL: CAST; IBP2: IGFBP2; FIP1: FIP1L1; E41L5: EPB41L5; CP071: C16orf71; CP037: C8orf37.

Supplementary Figure 3.11: Anisotropy competition assay plotted as difference in fluorescence anisotropy.

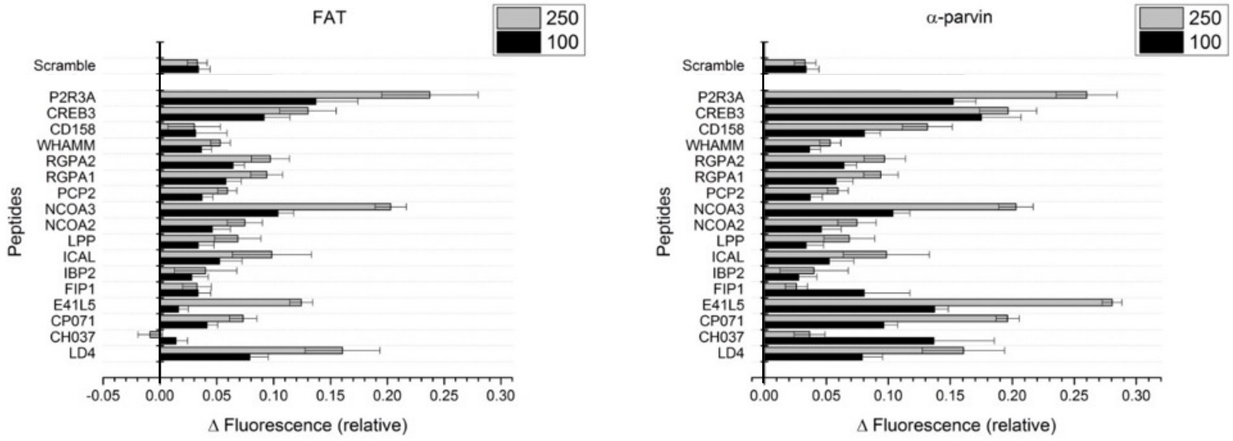

Proteins were kept at a concentration corresponding to the K_d of their interaction with labeled LD4 (10 μM for FAT and 25 μM for α-parvin), in the presence of 0.1 μM labeled LD4. To that, each non-labeled LD motif candidate peptide was added at 100 or 250 μM. Plotted are the resulting relative changes of the fluorescence anisotropy in presence of the unlabeled candidate peptides. The Uniprot identifiers are given instead of protein gene names. Genes were both identifiers differ are P2R3A: PPP2R3A; CD158:CCDC158; RGPA1/2: RALGAPA1/2; ICAL: CAST; IBP2: IGFBP2; FIP1: FIP1L1; E41L5: EPB41L5; CP071: C16orf71; CP037: C8orf37.

Supplementary Figure 3.12: Titration of FAT on to LD motifs

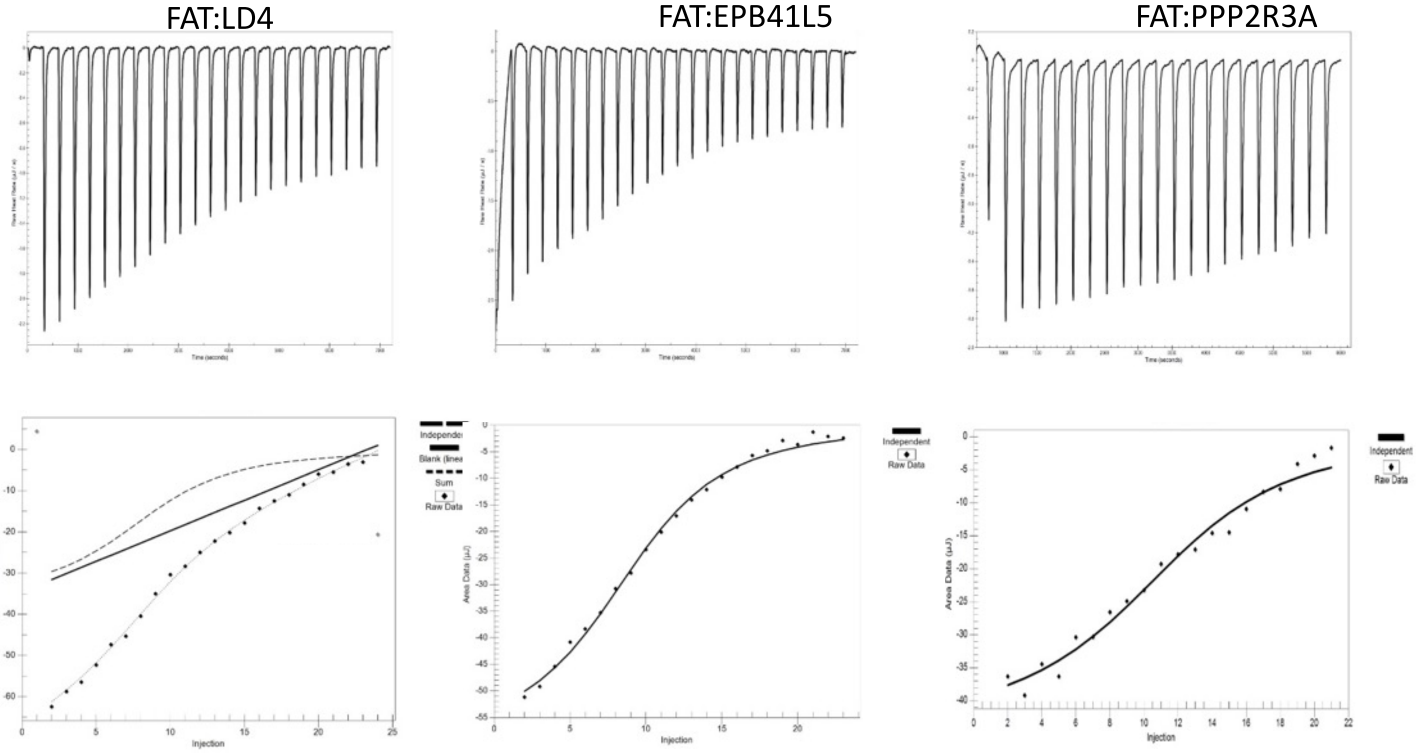

Supplementary Figure 3.13: Titration of GIT1 on to LD2 and EPB41L5

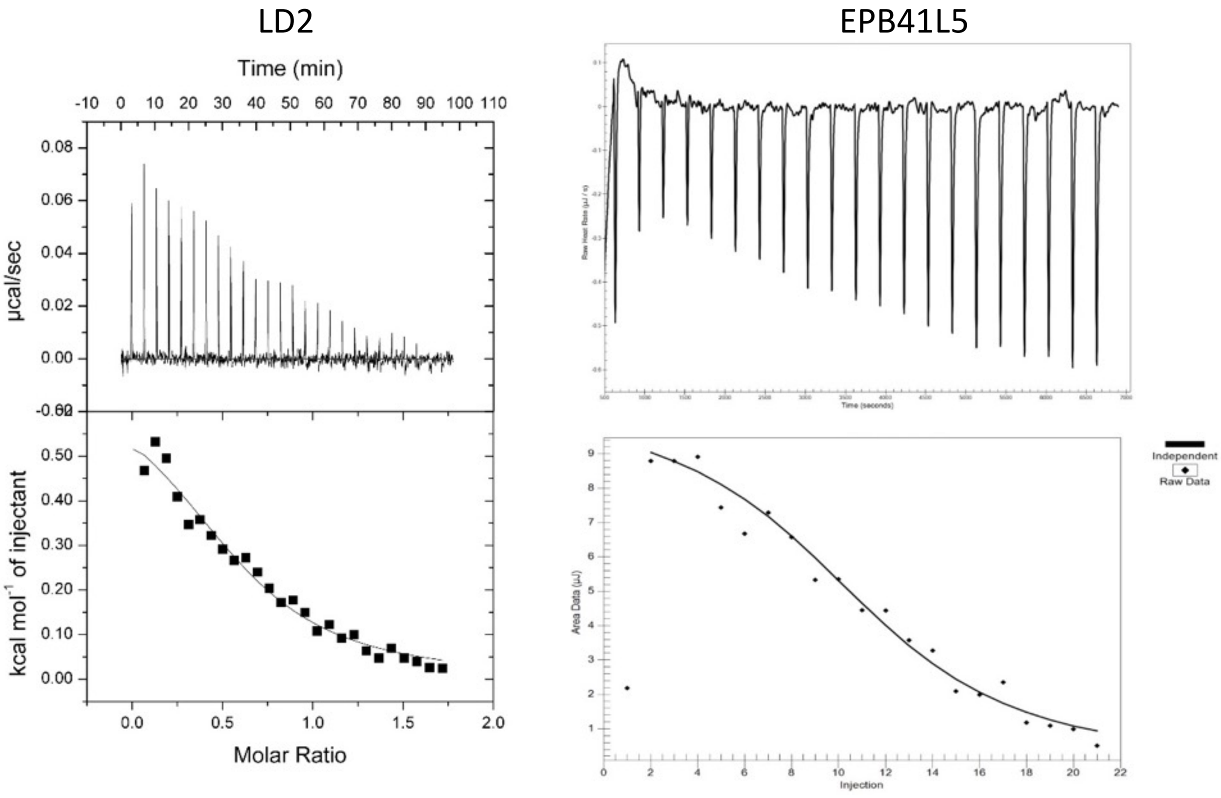

Supplementary Figure 4: RaptorX results for predicted human proteins

		20 residues upstream of predicted motif			20 residues downstream of predicted motif		
LD motif region of protein	LD motif region of protein (Uniprot Name)	Exposed	Moderately exposed	Buried	Exposed	Moderately exposed	Buried
EPB41L5	E41L5_HUMAN	18	2	0	10	9	1
LPP	LPP_HUMAN	19	1	0	20	0	0
RALGAPA1	RGPA1_HUMAN	15	4	1	16	3	1
PPP2R3A	P2R3A_HUMAN	20	0	0	18	0	2
CCDC158	CD158_HUMAN	20	0	0	18	1	1
C16orf71	CP071_HUMAN	13	7	1	17	1	2
NCOA2	NCOA2_HUMAN	20	0	0	16	3	1
NCOA3	NCOA3_HUMAN	20	0	0	17	1	2
CAST	ICAL_HUMAN	20	0	0	20	0	0
CREB3	CREB3_HUMAN	15	2	3	11	7	2
RALGAPA2	RGPA2_HUMAN	20	0	0	15	4	1
C8org37	CH037_HUMAN	3	-	-	20	0	0

Summary of RaptorX results for predicted 12 proteins

Secondary structure predictions (SS3: three states, namely H: helix, E: beta strand, C: coil; SS8: eight states, namely H: α helix, G: 3-helix, I: 5-helix, E: extended β ladder, B: β bridge, T: hydrogen bonded turn, S: bend, L: loop), solvent accessibility (ACC; B: buried; M: medium exposed, E: solvent exposed) and disorder (DISO: order [.] and disorder [*]) for the non-paxillin motifs suggested by SlimSearch4 (Krystkowiak and Davey, 2017), which was the only algorithm which predicted a reasonable number of LD motif candidate in the human proteome (see **Supplementary Table 1**). The feature predictions were established by the RaptorX server (Kallberg, et al., 2014). The suggested LD motif region is boxed. Amino acid are numbered starting with 20 positions upstream of the LD motif (unless the LD motif is situated at the N-terminus, which is then taken as number 1).

E41L5_HUMAN

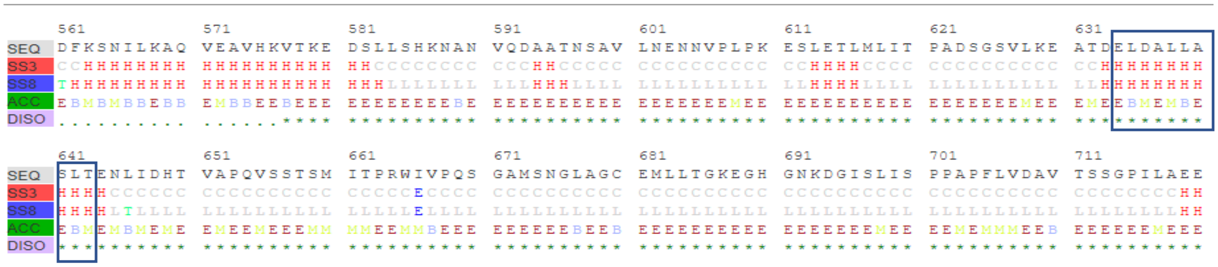

LPP_HUMAN

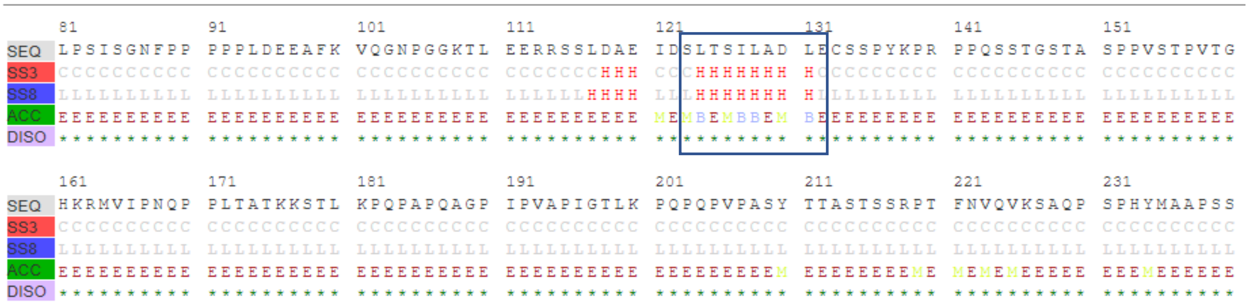

RGPA1_HUMAN

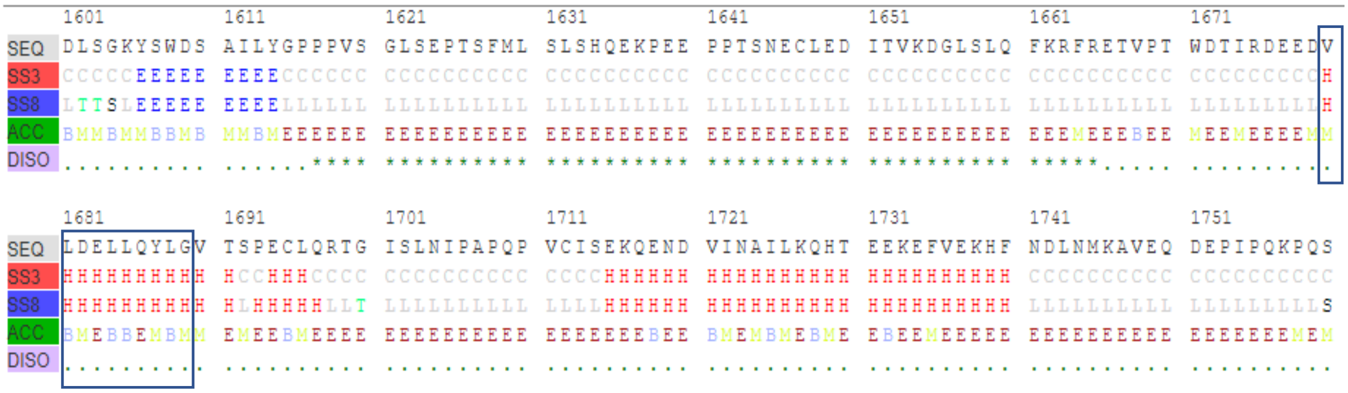

P2R3A_HUMAN

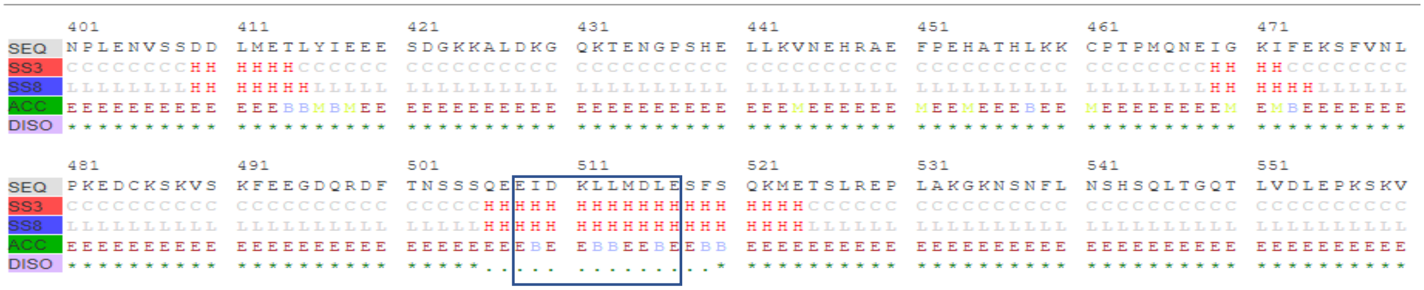

CD158_HUMAN

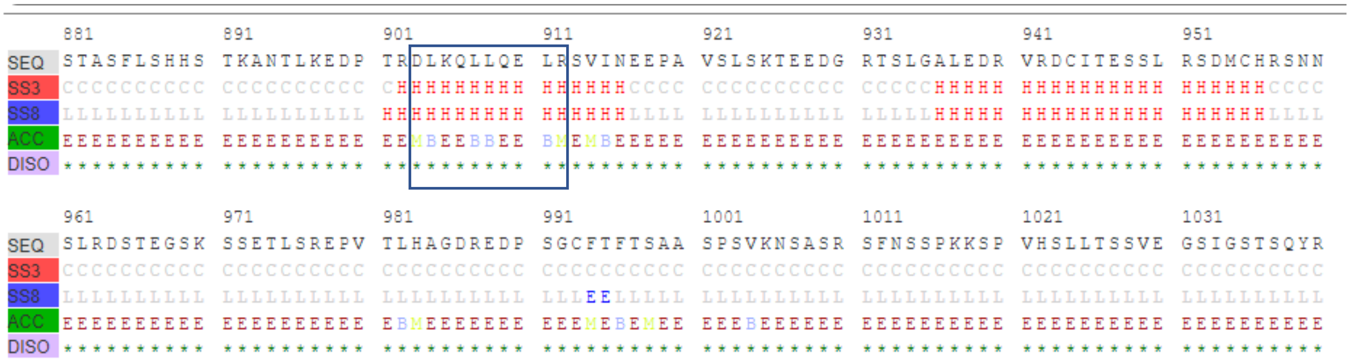

CP071_HUMAN

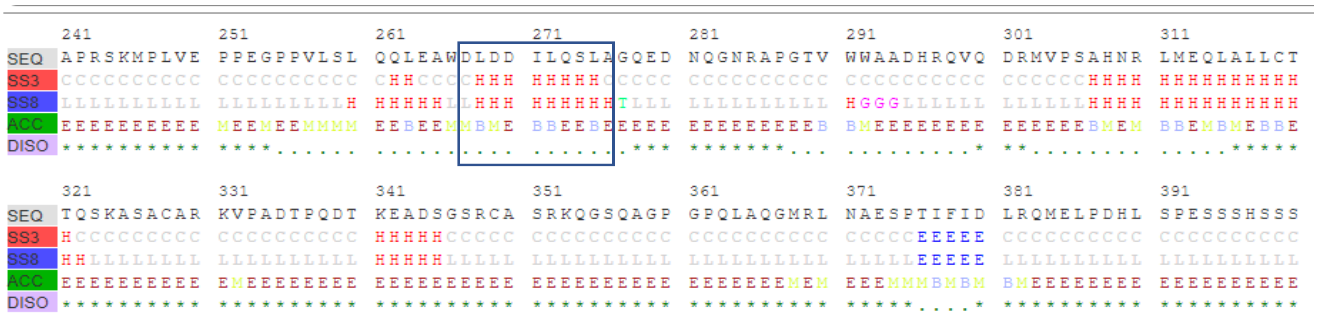

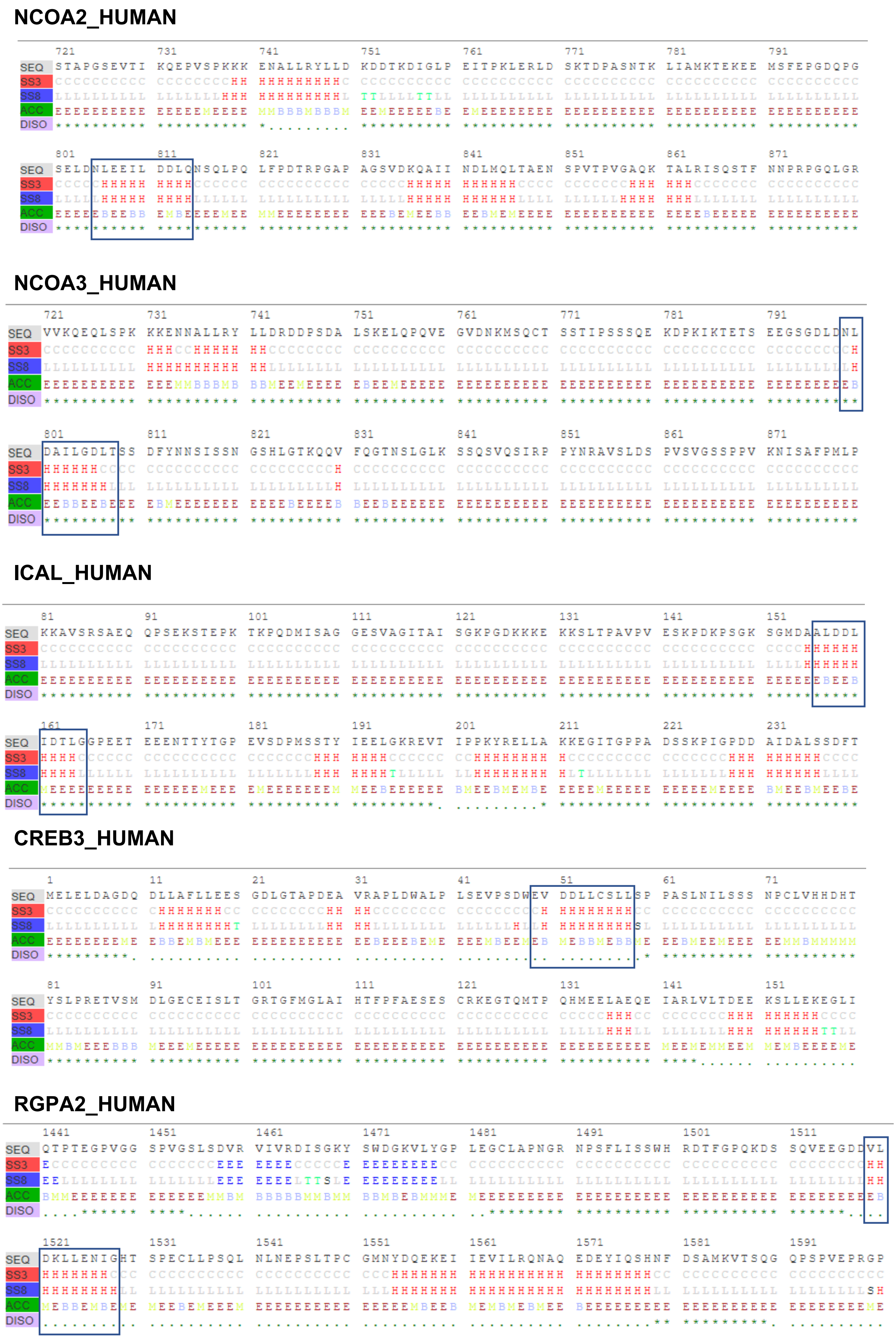
NCOA2_HUMAN
NCOA3_HUMAN
ICAL_HUMAN
CREB3_HUMAN
RGPA2_HUMAN

CH037_HUMAN

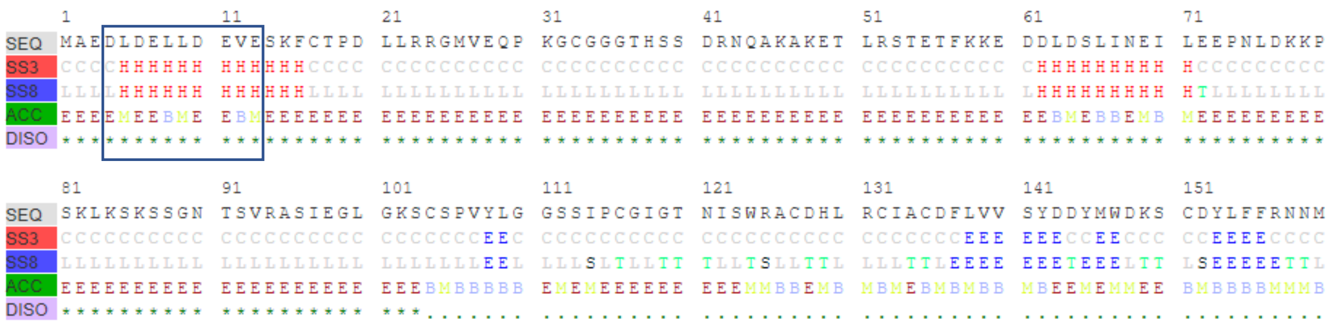

Supplementary Figure 5:

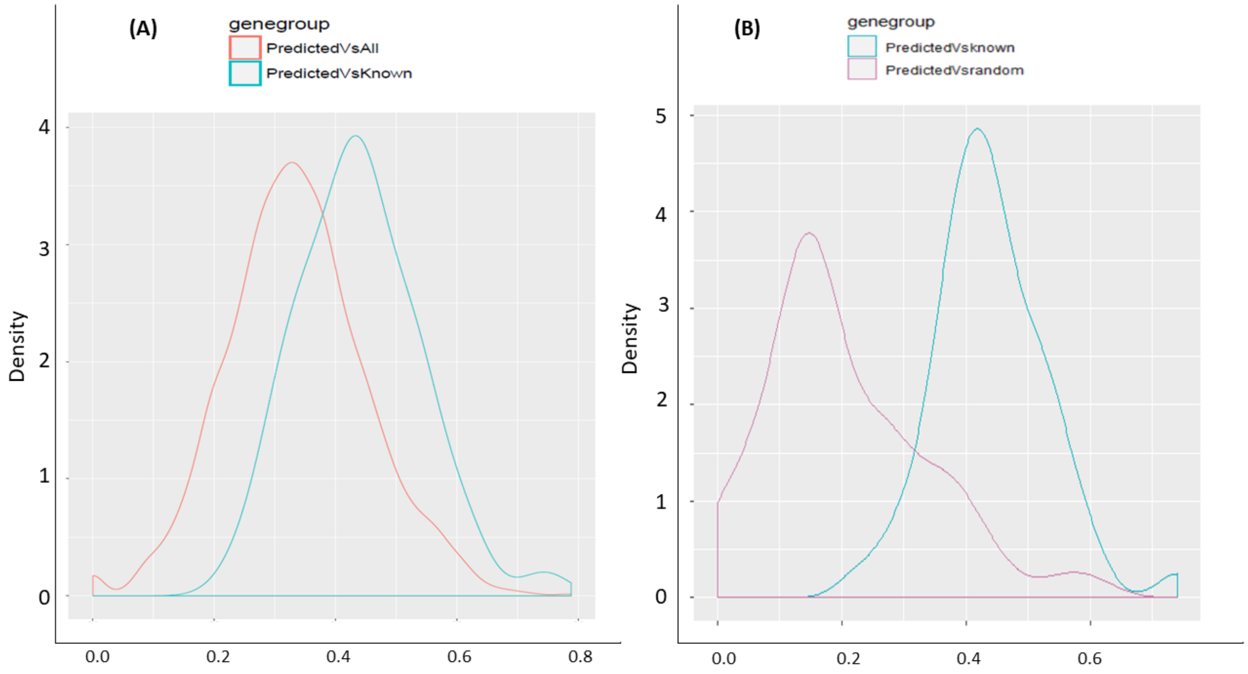

GO ANALYSIS: **A)** Distribution of Semantic Similarity between LDMF-predicted proteins and known LD motif proteins (cyan) and between LDMF-predicted proteins and all proteins, except the known LD motif proteins (red). The p-value of Mann-Whitney U test for the distributions is 6.32e-10.

B) Distribution of Semantic Similarity between LDMF-predicted proteins and known LD motif proteins (cyan) and between LDMF-predicted proteins and same number of random proteins from human (red). The p-value of Mann-Whitney U test for the distributions is 30648 e-14.

Supplementary Figure 6: ^{1}H-^{15}N HSQC titration experiments.

Shown are the NMR chemical shifts of ^{15}N-labelled FAT domain titrated with LD4, LD2, LPP, and CD158.

Supplementary Figure 6.1: FAT/LD2 Titration.

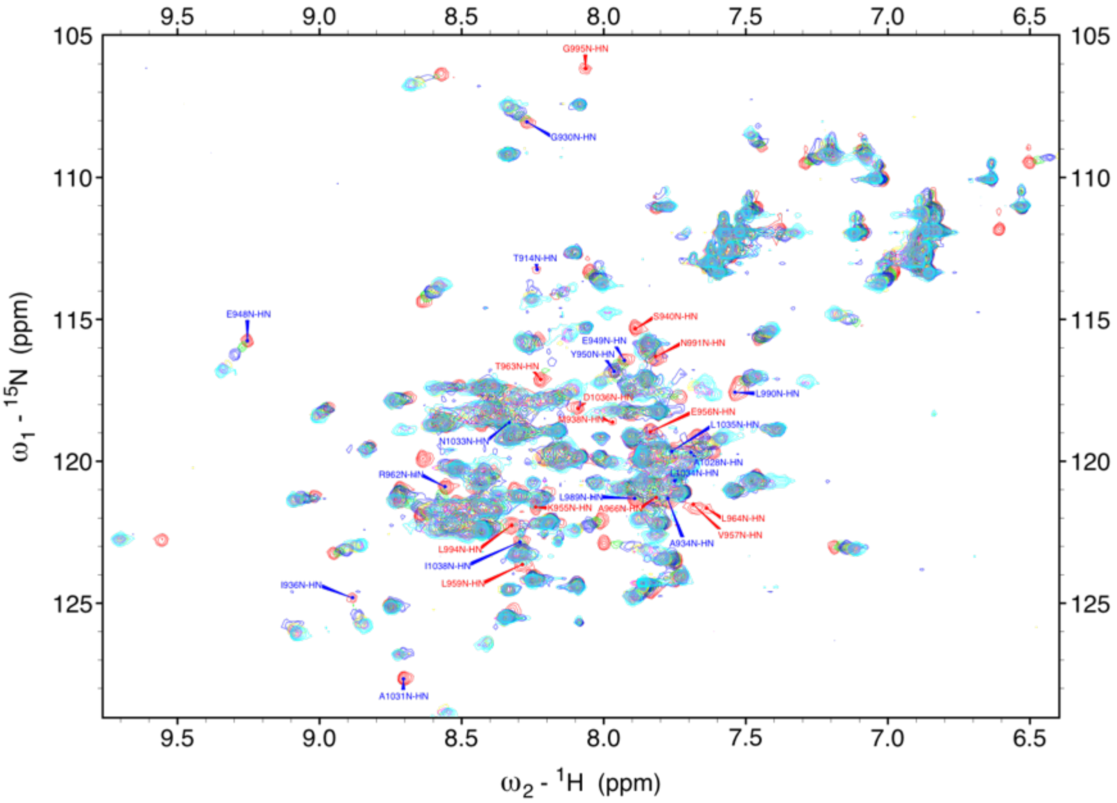

Overlay of ^{1}H-^{15}N-HSQC spectra of 100 μM ^{15}N-FAT in the absence (red) and presence of 0.5 (green), 1 (blue), 2 (yellow), 3 (magenta) and 4 (cyan) times molar excess of LD2 peptide. Resonances that disappeared upon LD2 addition are labelled in red. Resonances that significantly shifted >2σ= 0.16 are labelled in blue. All spectra were recorded at 25°C at a proton frequency of 950 MHz.

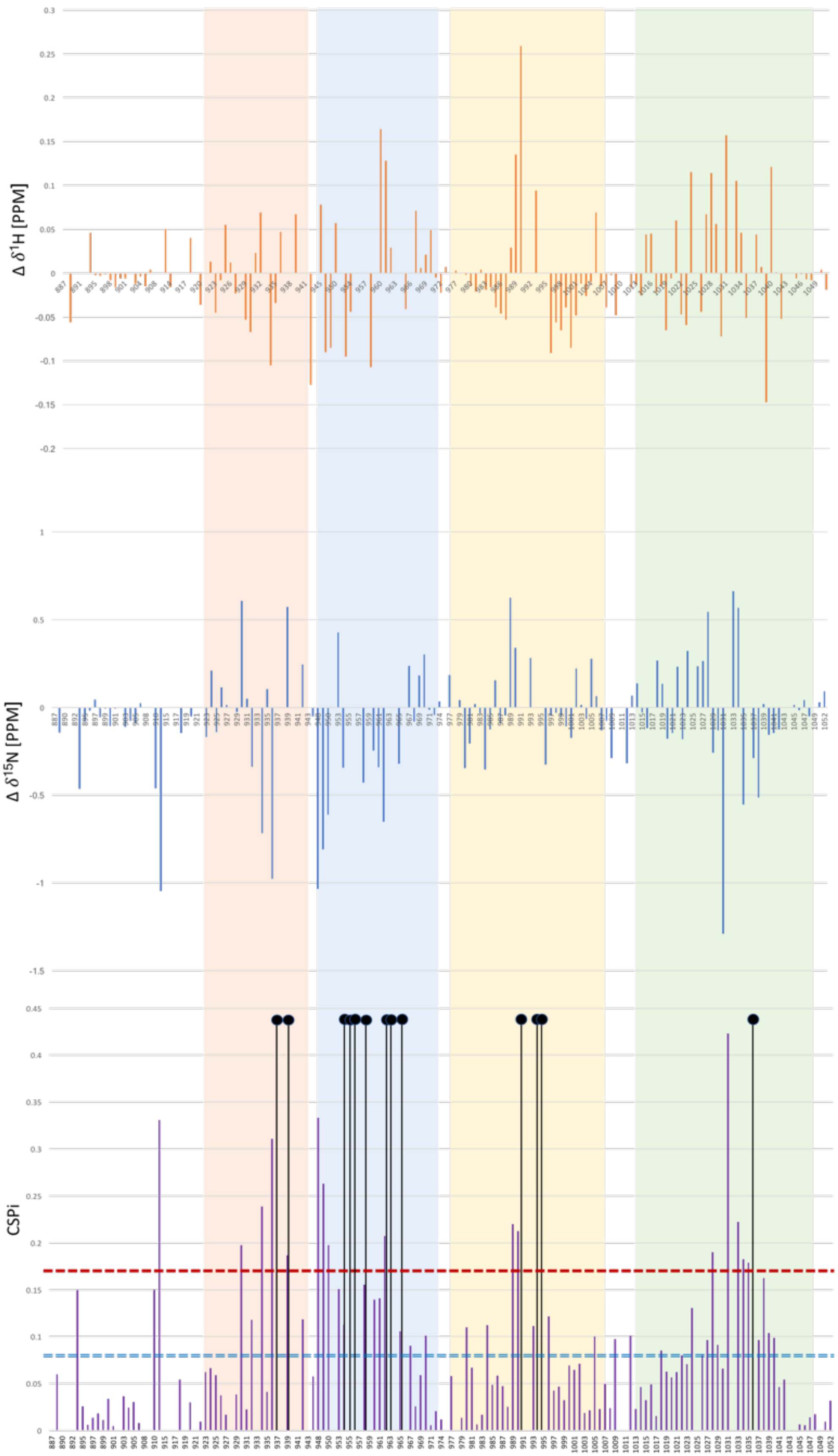

Supplementary Figure 6.2: Chemical shift changes in FAT induced by the LD2 peptide. Chemical shift differences in ppm where calculated for ^{1}H (top panel), ^{15}N (middle panel) and the weighted combined ^{1}H,^{15}N (lower panel) chemical shift perturbation of FAT in the presence of a four times molar excess of LD2 peptide. Red dashed line indicates the upper threshold of 2σ= 0.16 and the blue double-dashed line indicates the lower threshold of σ= 0.08. Others that disappeared upon LD2 addition are marked by full black circles. The shaded areas represent the helices (orange for helix1, blue for helix2, yellow for helix3, green for helix4).

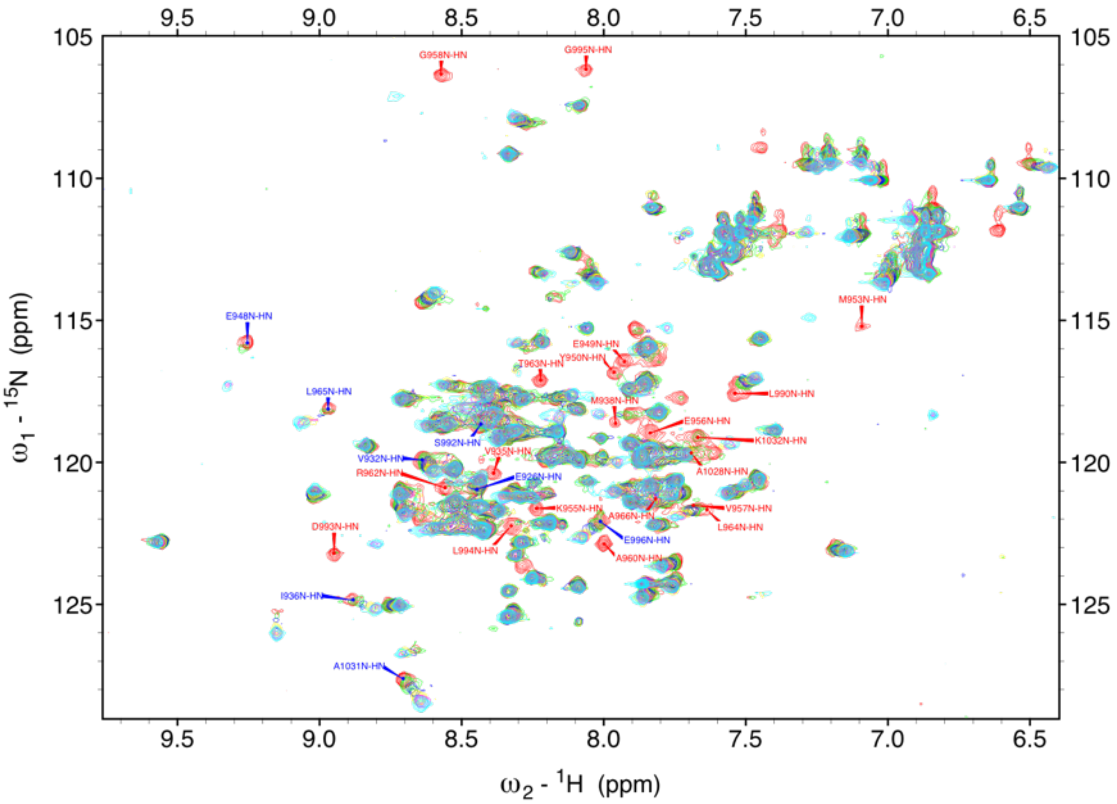

Supplementary Figure 6.3: FAT/LD4 Titration. Overlay of ^{1}H-^{15}N-HSQC spectra of 100 µM ^{15}N-FAT in the absence (red) and presence of 0.5 (green), 1 (blue), 2 (yellow), 3 (magenta) and 4 (cyan) times molar excess of LD4 peptide. Resonances that disappeared upon LD4 addition are labelled in red. Resonances that significantly shifted >2σ= 0.12 are labelled in blue. All spectra were recorded at 25°C at a proton frequency of 950 MHz.

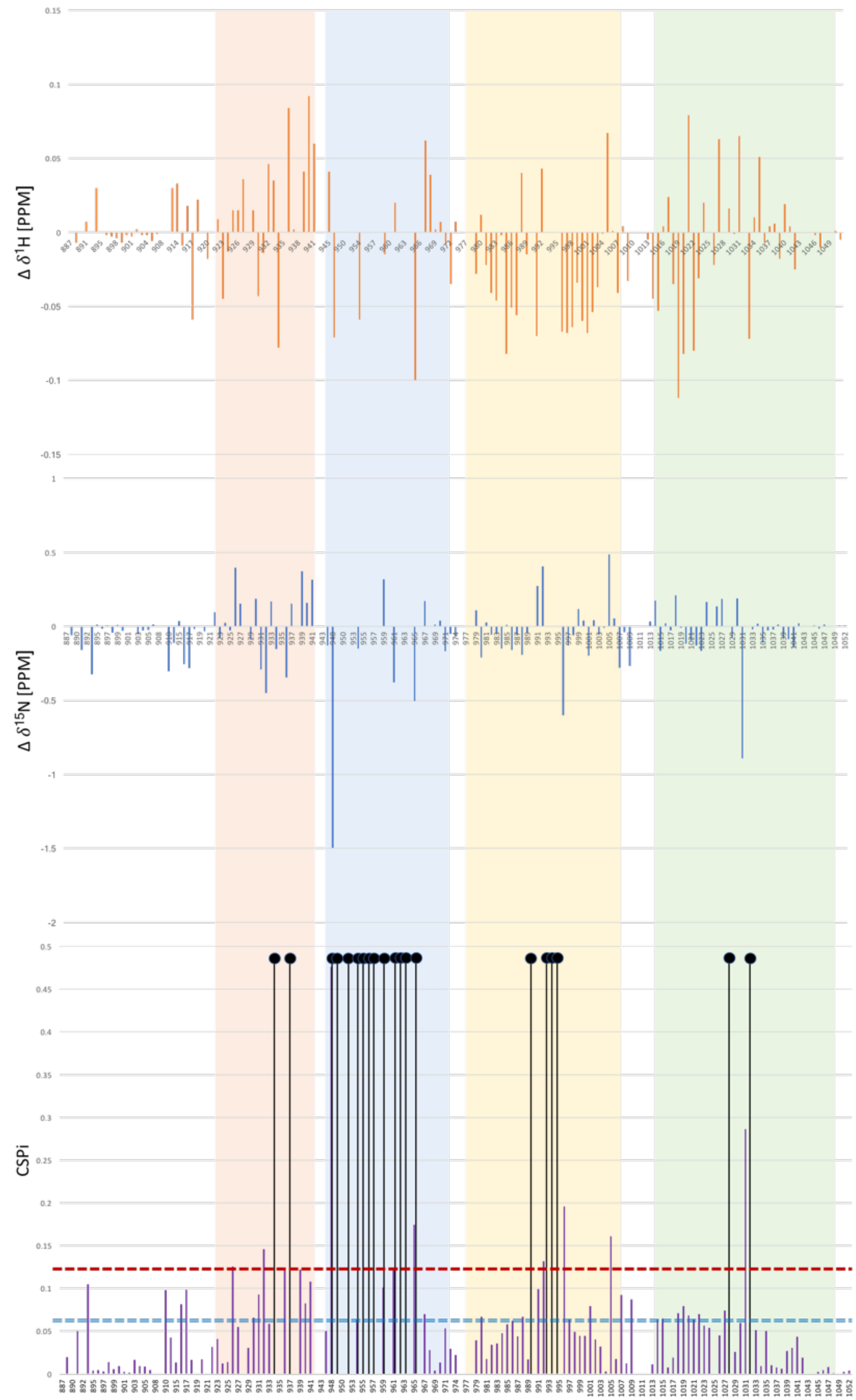

Supplementary Figure 6.4: Chemical shift changes in FAT induced by LD4 peptide. Chemical shift differences in ppm where calculated for ^{1}H (top panel), ^{15}N (middle panel) and the weighted combined ^{1}H,^{15}N (lower panel) chemical shift perturbation of FAT in the presence of a four times molar excess of LD4 peptide. Red dashed line indicates the upper threshold of 2σ= 0.12 and the blue double-dashed line indicates the lower threshold of σ= 0.06. Others that disappeared upon LD4 addition are marked by full black circles. The shaded areas represent the helices (orange for helix1, blue for helix2, yellow for helix3, green for helix4).

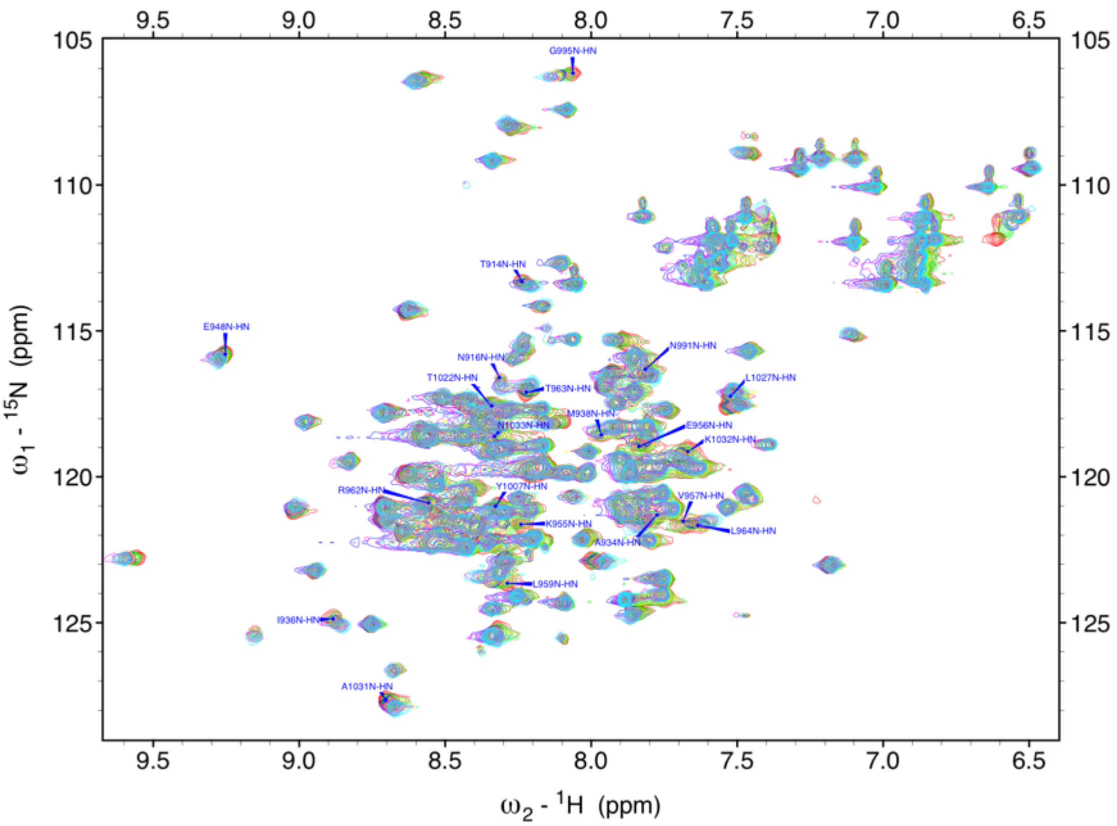

Supplementary Figure 6.5: FAT/LPP titration. Overlay of ^{1}H-^{15}N-HSQC spectra of 100 μM ^{15}N-FAT in the absence (red) and presence of 1 (green), 2 (blue), 3 (yellow), 4 (magenta) and 5 (cyan) times molar excess of LPP peptide. Resonances that disappeared upon LPP addition are labelled in red. Resonances that significantly shifted >2σ= 0.06 are labelled in blue. All spectra were recorded at 25°C at a proton frequency of 950 MHz.

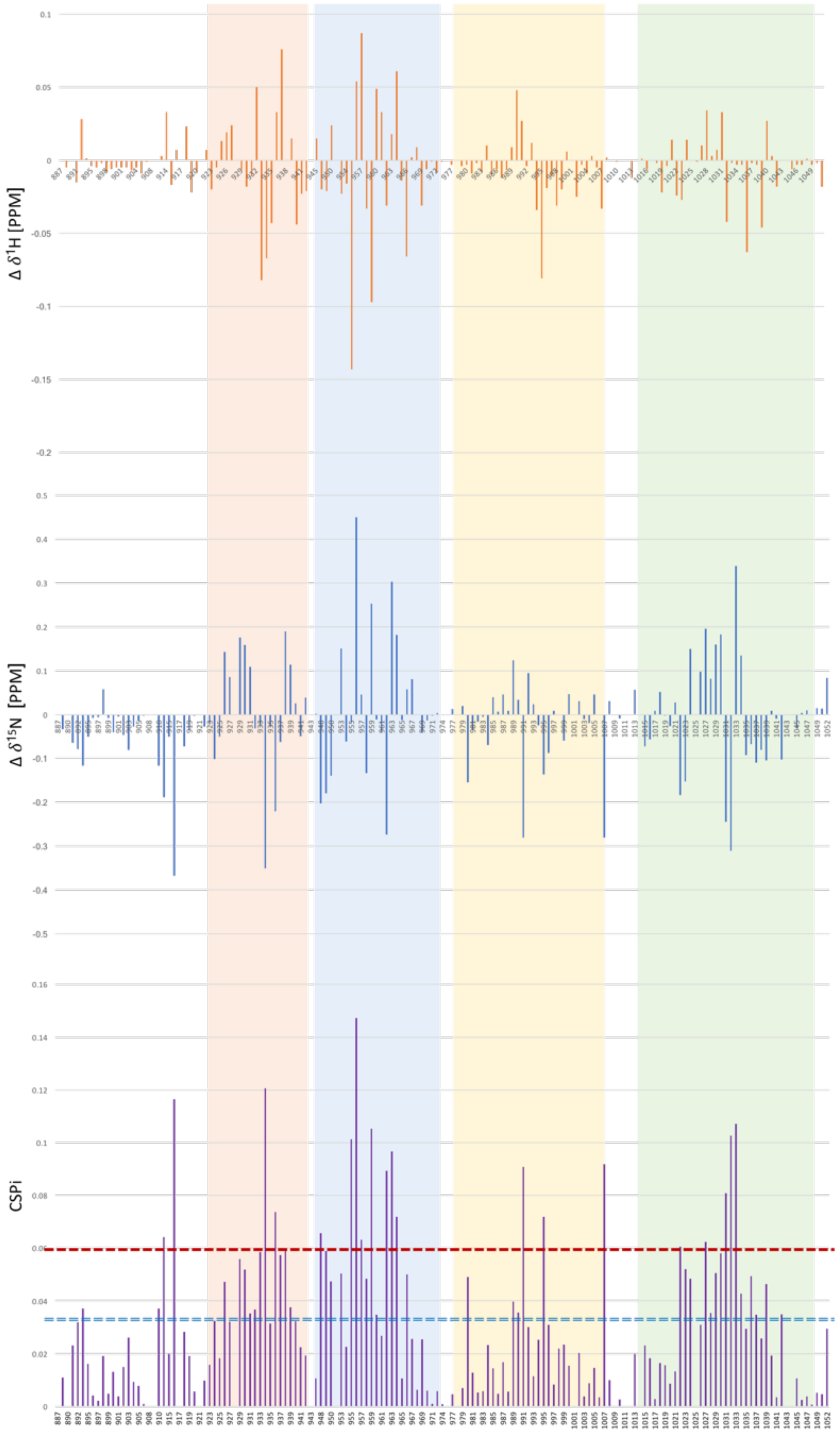

Supplementary Figure 6.6: Chemical shift changes in FAT induced by LPP peptide. Chemical shift differences in ppm where calculated for ^{1}H (top panel), ^{15}N (middle panel) and the weighted combined ^{1}H,^{15}N (lower panel) chemical shift perturbation of FAT in the presence of a five times molar excess of LPP peptide. Red dashed line indicates the upper threshold of 2σ= 0.06 and the blue double-dashed line indicates the lower threshold of σ= 0.03. The shaded areas represent the helices (orange for helix1, blue for helix2, yellow for helix3, green for helix4).

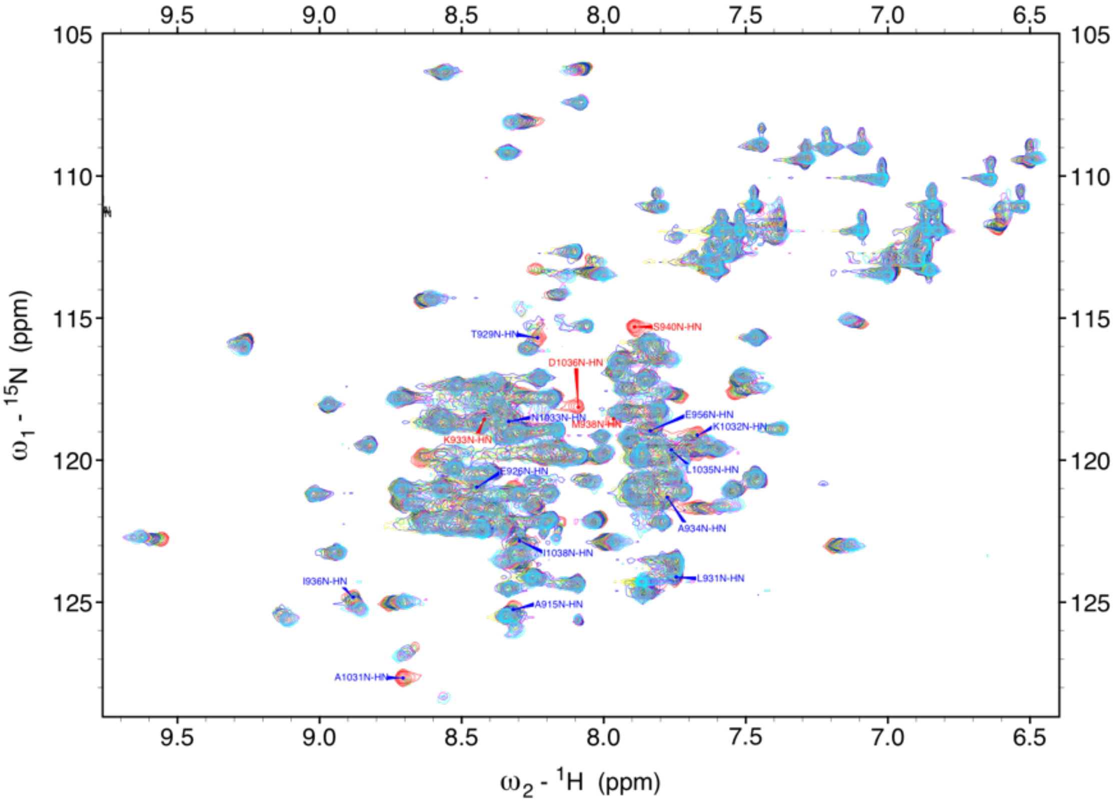

Supplementary Figure 6.7: FAT/CCDC158 Titration. Overlay of 1H15N-HSQC spectra of 100 μM 15N-FAT in the absence (red) and presence of 0.5 (green), 1 (blue), 2 (yellow), 3 (magenta) and 4 (cyan) times molar excess of CCDC158 peptide. Resonances that disappeared upon CCDC158 addition are labelled in red. Resonances that significantly shifted >2σ= 0.114 are labelled in blue. All spectra were recorded at 25°C at a proton frequency of 900 MHz.

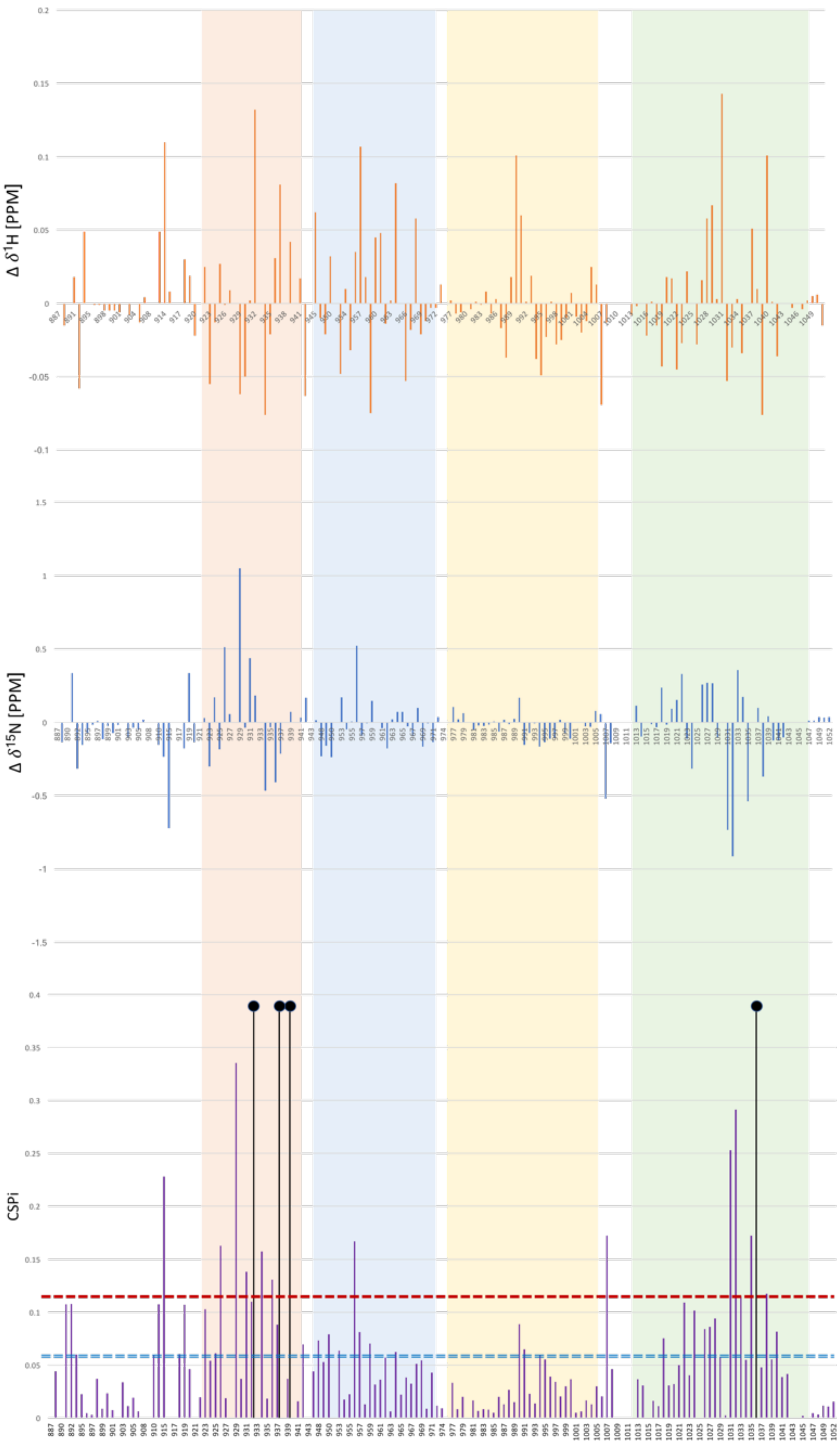

Supplementary Figure 6.8: Chemical shift changes in FAT induced by CCDC158 peptide. Chemical shift differences in ppm where calculated for ^{1}H (top panel), ^{15}N (middle panel) and the weighted combined ^{1}H, ^{15}N (lower panel) chemical shift perturbation of FAT in the presence of a four times molar excess of CCDC158 peptide. Red dashed line indicates the upper threshold of 2σ= 0.114 and the blue double-dashed line indicates the lower threshold of σ= 0.057. Others that disappeared upon CCDC158 addition are marked by full black circles. The shaded areas represent the helices (orange for helix1, blue for helix2, yellow for helix3, green for helix4).

Supplementary Figure 7: Analysis of homology among unicellular LD motifs candidates.

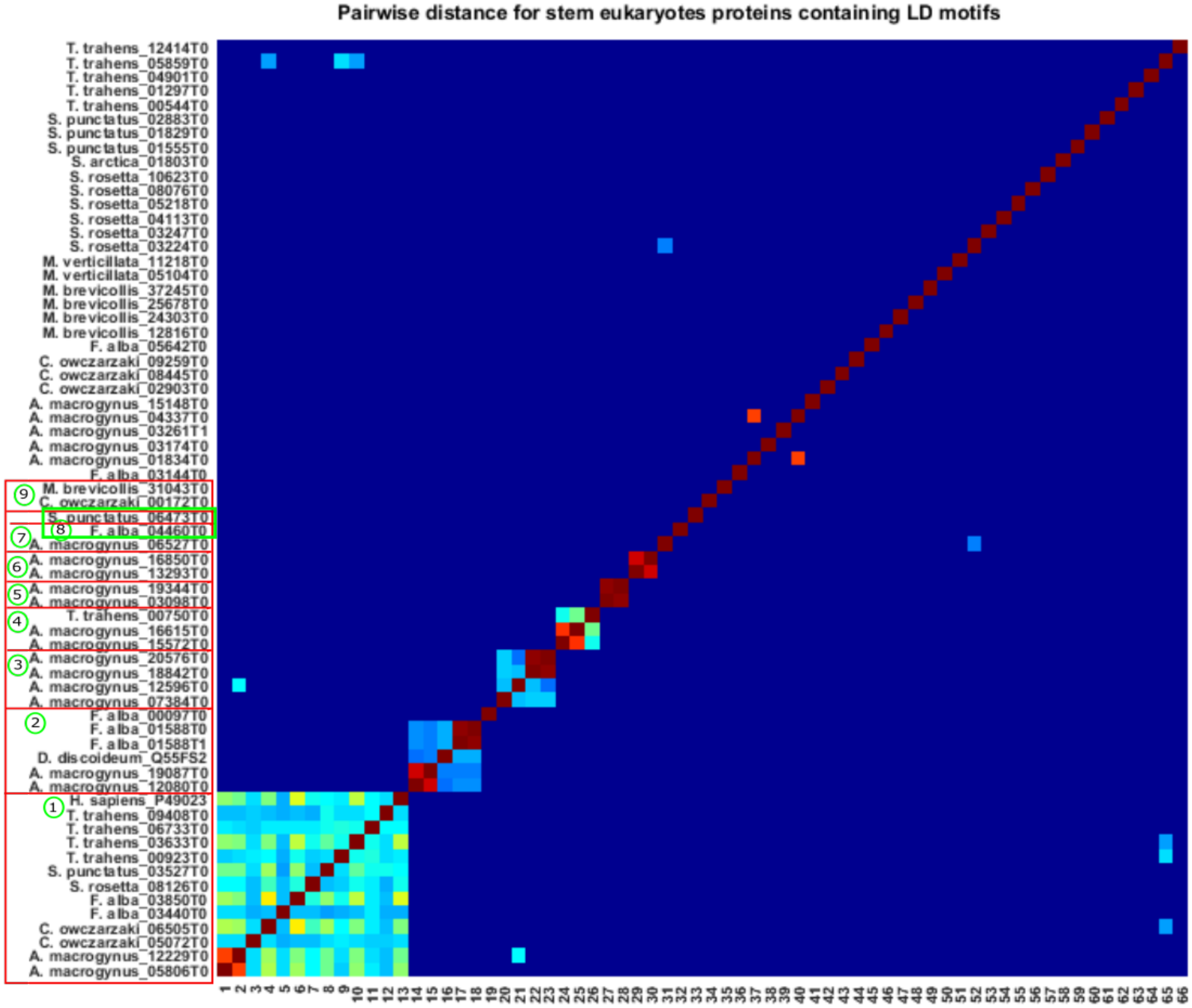

The conservation of LD motif-containing proteins in unicellular eukaryotes. Heat map shows pairwise identity matrix (in percentage) where E-value<1e-10. Proteins with annotated domains from PFAM are clustered on the sequence labels (on Y-axis) as follow: (1) LIM domain, (2) Protein kinase domain, (3) Formin Homology 2, (4) Retinal Maintenance, (5) Ubiquitin-activating enzyme active site (Thif family), (6) Mitochondrial carrier protein, (7) Ankyrin repeat, (8) RasGEF domain (RhoGEF), and (9) Ras association (RalGDS/AF-6) domain. The Y-axis shows the gene names. Each gene starts with the abbreviation of the species coming from. Abbreviations are as follows (1) Homo sapiens: *Homo sapiens*, (2) C. owczarzaki: *Capsaspora owczarzaki*, (3) M. brevicollis: *Monosiga brevicollis*, (4) M. verticillata: *Mortierella verticillata*, (5) D. discoideum: *Dictyostelium discoideum*, (6) S. arctica: *Sphaeroforma arctica*, (7) S. rosetta: *Salpingoeca rosetta*, (8) S. punctatus: *Spizellomyces punctatus*, (9) F. alba: *Fonticula alba*, (10) T. trahens: *Thecamonas trahens*, and (11) A. macrogynus: *Allomyces macrogynus*.

Supplementary Figure 8: Conservation of human non-paxillin LD motif proteins across species. In a first instance, we searched homologues of the newly identified LD motif-containing proteins in the most commonly used model species (i.e. fruit fly, zebrafish, chicken and mouse). When a homologue was not found in the model species, we considered other close species that have been sequenced (such as sharks or ostrich). The region encompassing the identified 10-residue LD motifs in humans are boxed.

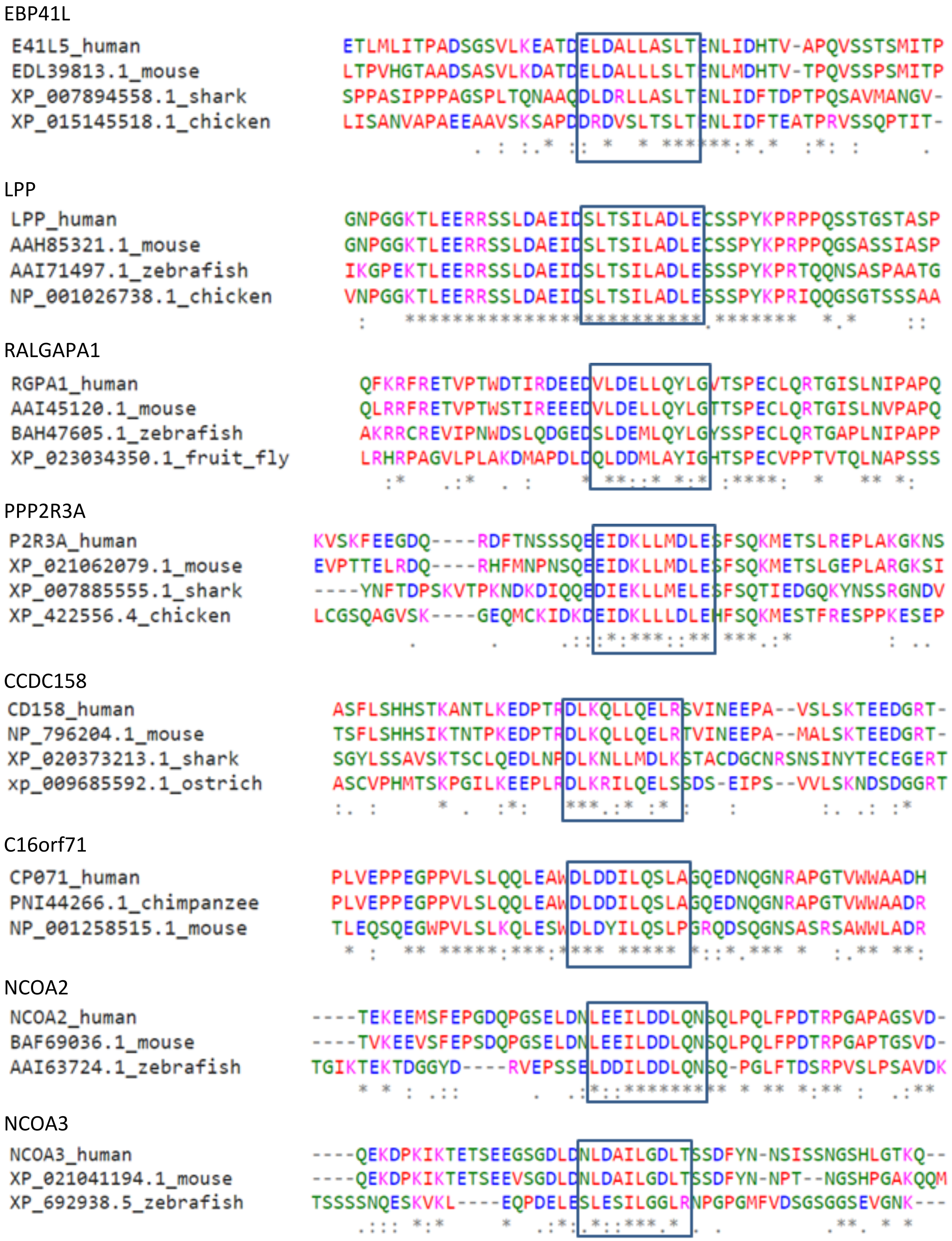

CAST

```
ICAL_human                PAVPVESKPDKPSGKSGM-DAALDDLIDTLGGPEETEEENTTYTGPEVSDP-
ICAL_mouse                PASPVQSTPSKPSDKSGM-DAALDDLIDTLGGHEDTNRDDPPYTGPVVLDP-
XP_007891289.1_shark      AAFSVSASQPAPKGKTGD-MGALDALSDML-QPEAPVHSGPKYTGPEVKEKA
ABP68381.1_chicken        -VASMVAAADKPNSEPAMDESALDSLIDTLGGSEEDVATRPVYTGPEITEN-
                           .  : :    *..: .   .*** * * *   *        **** : :
```

CREB3

```
CREB3_human               EAVRAPLDWALPL----SEVPSDWEVDDLLCSLLSPPASLNILSSSNPCLVHHD
CREB3_mouse               --VKASLDLELSPSENSVQELSDWEVEDLLSSLLSPSVSRDVLGSSSSSILHD-
XP_009678890.1_ostrich    EESSLLEDWGLSD----AQLLNNKEMDDFISSLLSPFVDEPGTLQGYSPTDSDS
                                 *  *       :  .: *::*::.***** ..     ..      .
```

RALGAPA2

```
RGPA2_human               -WHRDTFGPQKDSSQVEEGDDVLDKLLENIGHTSPECLLPSQLNLNEPSLT
BAA92774.1_mouse          -QLRRFRETVPTWSTIQEEEDVLDELLQYLGTTSPECLQRTGISLNVPAPQ
XP_005158962.1_zebrafish  -RSSVRFSKCSSELDVEDGVDVLDQLLEDLGHSSPECLPEPQLRLTQPPSP
XP_023168385.1_fruit_fly  LRHR-PPGVLPLAKDVAPDLDQLDDMLAYIGHTSPECVPPTISELNAPSLS
                                         :    * **.:*  :* :****:      *. *
```

C8orf37

```
CH037_human               MAEDLDELLDEVESKFCTPDLLRRG---MVEQPKGC
NP_080281.3_mouse         MAKDLDELLDEVETKFCRLDPLRLD---LGERPKGD
XP_007886093.1_shark      MADDLDQLLDEVESRFCGQSAPRQRNKGAGERV---
                          **.***:******::**  .  *       *:
```

RoXaN

```
Z3H7B_human               RTLPSTDSLDDFSDGDVFGPELDTLLDSLSLVQGGLSGSGVPSELPQLIP----
NP_001074485.1_mouse      RTLPGTEGLDDFSDGDVFGPELDTLLDSLSLVQGGLPGSGMPSELPQLIP----
XP_007907761.1_shark      VSLSVPESTEEFTDGEIIGEDIDTLLDSIQD----YPMSSAPGPIPTNVPANVS
KF061317.1_chicken        VPLPVPENVEDFTDGDIIGEELDSLLDSLAEGS-PYPLGTIPTNLPTEMPQ---
                            *   :. ::*:**:::* ::*:****:         .  *  :*  :*
```

DLC1

```
RHG07_human               GSILYSSSGDLADLENEDIFPELDDILYHVKGMQRIVNQWSEKFSDEGDS
XP_021074615.1_mouse      GSILYSSSGELADLENEDIFPELDDILYHVKGMQRIVNQWSEKFSDEGDS
XP_007889463.1_shark      GSHLYASTGDLLDLEKEDIFPHLDDILQHVNGLQQIVDHWSKNVLPELQD
                          ** **:*:*:* ***:*****.***** **:*:*:**::**::.  * :.
```

Supplementary Figure 9: Spreading Assay

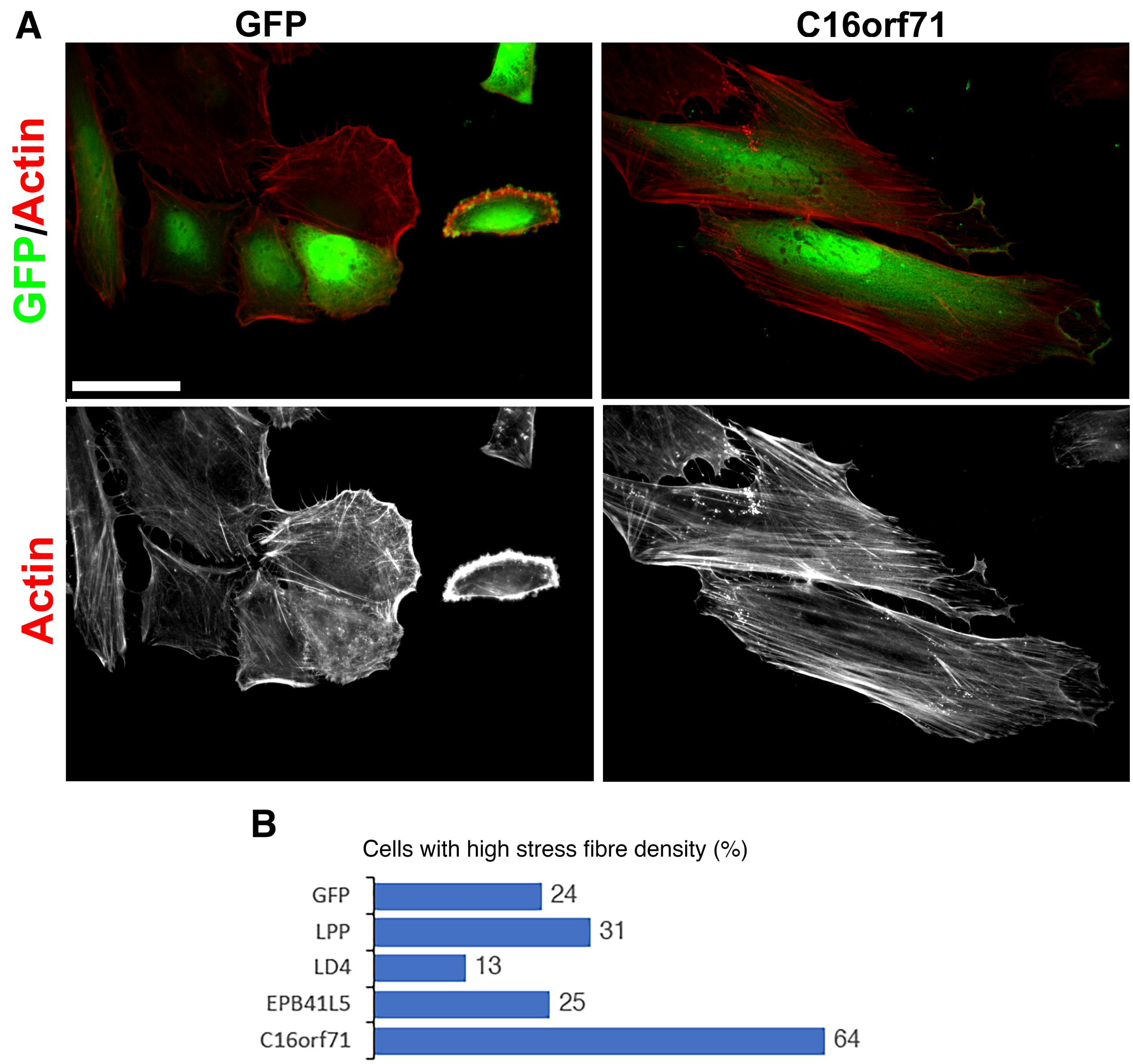

A) HeLa cells transfected with GFP alone (control cells) and GFP-tagged C16orf71 were plated on fibronectin, fixed and stained for the indicated antibodies. B) A large meshwork of actin stress fibres is observed in ^{eGFP}C16orf71, but not in control cells. The percentage of cells with high stress fibre density is shown. The quantification was performed on 23-42 cells. Scale bar= 50 µm.

Supplementary Table 1: Prediction results from other existing tools

Table 1.1. Predictions for known LD motifs

We used [LV] [DE] X [LM] [LM] XXL as a regular expression for generating output from existing tools. SlimSearch4 returned 37 proteins (44 motifs). PSSM search returned 881 proteins (1000 motifs). FIMO search returned 1432 proteins (1614 motifs). The table shows the amino acid positions of known human LD motifs. Rank refers to a motif's rank based on the conservation score for SlimSearch4, based on the PWM p-value for PSSMSearch, and based on the p-value for FIMO.

Index	Protein name	Start position	End position	Uniprot ID	SlimSearch4 rank	PSSMSearch rank	FIMO rank
**1**	PXN	3	12	P49023	12	6	447
**2**	PXN	144	153	P49023	1	167	460
**3**	PXN	216	225	P49023	14	22	386
**4**	PXN	265	274	P49023	Not found	Not found	216
**5**	PXN	333	342	P49023	8	11	235
**6**	LPXN	3	12	O60711	3	35	453
**7**	LPXN	92	101	O60711	2	85	43
**8**	LPXN	127	136	O60711	5	114	223
**9**	Hic-5	3	12	O43294	4	2	448
**10**	Hic-5	92	101	O43294	Not found	Not found	305
**11**	Hic-5	157	166	O43294	6	4	196
**12**	Hic-5	203	212	O43294	5	152	233
**13**	RoXaN	280	289	Q9UGR2	Not found	Not found	420
**14**	DLC1	905	914	Q96QB1	Not found	Not found	Not found

Supplementary Table 1.2: Predictions for additional LD motifs in the human proteome

We used [LV] [DE] X [LM] [LM] XXL as a regular expression for generating output from existing tools. SlimSearch4 returned 37 proteins (44 hits). PSSM search returned 881 proteins (1000 hits). PSSM search rank is out of 1000 hits. FIMO search returned 1432 proteins (1614 hits). FIMO search rank is out of 1614 hits. The table reports the rank of the LDMF predicted 12 proteins, based on the conservation score for SlimSearch4; based on the PWM p-value for PSSMSearch; based on p-value for FIMO (Grant, et al., 2011; Krystkowiak and Davey, 2017; Krystkowiak, et al., 2018).

Index	Protein name	Start position	End position	Uniprot ID	SlimSearch 4 rank	PSSMSearch rank	FIMO rank
1	EPB41L5	634	643	Q9HCM4	Not found	Not found	518
2	LPP	123	132	Q93052	Not found	515	Not found
3	RALGAPA1	1680	1689	Q6GYQ0	Not found	Not found	236
4	PPP2R3A	508	517	Q06190	Not found	10	Not found
5	CCDC158	903	912	Q5M9N0	Not found	40	512
6	C16orf071	267	276	Q8IYS4	Not found	101	Not found
7	NCOA2	805	814	Q15596	Not found	42	Not found
8	NCOA3	799	808	Q9Y6Q9	Not found	63	Not found
9	CAST	156	165	P20810	Not found	33	Not found
10	CREB3	49	58	O43889	Not found	Not found	122
11	RALGAPA2	1519	1528	Q2PPJ7	Not found	70	1432
12	C8orf37	4	13	Q96NL8	Not found	163	Not found

Supplementary Table 2: Results of predictions from final model

Prediction results of the final LDMF model using different combination of features

Features	Number of Features	Sensitivity (%)	Specificity (%)	Accuracy (%)
All	40	88.889	100.00	99.968
Sequence	5	83.333	99.968	99.921
Secondary Structure	5	94.444	80.251	80.292
AAindex	30	66.667	97.143	97.056

The sensitivity, specificity, accuracy stated are based on the performance of the machine-learning model on the test set. We used the known LD motifs to build the machine learning model. We then tested the performance of the computational model using a leave-one-out cross validation approach. Given the imbalanced nature of our training data, 'sensitivity' appears as the most appropriate evaluation metric.

Supplementary Table 3: Round1-round2_predictions

The LD motif sequences used in LDMF are given, according to: *bona fide* LD motifs used in the initial training of LDMF, and LD motif candidates predicted in the second round of LDMF.

Supplementary Table 3.A: Information for the *bona fide* LD motifs.

<table>
<tr><th>Index</th><th>Protein name</th><th>Start position</th><th>End position</th></tr>
<tr><td>1.</td><td>Paxillin | PXN</td><td>3</td><td>12</td></tr>
<tr><td colspan="4">Primary and secondary sequence
------------------MDDLDALLADLESTTSHISKRPVFLSEETPYS
------------------CCCHHHHHHHHHHCCCCCCCCCCCCCCCCCCC</td></tr>
<tr><td>2.</td><td>Paxillin | PXN</td><td>144</td><td>153</td></tr>
<tr><td colspan="4">Primary and secondary sequence
QKSAEPSPTVMSTSLGSNLSELDRLLLELNAVQHNPPGFPADEANSSPPL
CCCCCCCCCCCCCCCCCCHHHHHHHHHHHHCCCCCCCCCCCCCCCCCCCC</td></tr>
<tr><td>3.</td><td>Paxillin | PXN</td><td>216</td><td>225</td></tr>
<tr><td colspan="4">Primary and secondary sequence
PLTKEKPKRNGGRGLEDVRPSVESLLDELESSVPSPVPAITVNQGEMSSP
CCCCCCCCCCCCCCCCCCCCCHHHHCCCCCCCCCCCCCCCCCCCCCCCCC</td></tr>
<tr><td>4.</td><td>Paxillin | PXN</td><td>265</td><td>274</td></tr>
<tr><td colspan="4">Primary and secondary sequence
PQRVTSTQQQTRISASSATRELDELMASLSDFKIQGLEQRADGERCWAAG
CCCCCCCCCCCCCCCCCCCCCHHHHHCCCCCCCCCCCCCCCCCCCCCCCC</td></tr>
<tr><td>5.</td><td>Paxillin | PXN</td><td>333</td><td>342</td></tr>
<tr><td colspan="4">Primary and secondary sequence
MAQGKTGSSSPPGGPPKPGSQLDSMLGSLQSDLNKLGVATVAKGVCGACK
CCCCCCCCCCCCCCCCCCCCCCCCHHHHHHHHHHHHCCCCCCCCCCCCCC</td></tr>
<tr><td>6.</td><td>Leupaxin | LPXN</td><td>3</td><td>12</td></tr>
<tr><td colspan="4">Primary and secondary sequence
------------------MEELDALLEELERSTLQDSDEYSNPAPLPLDQ
------------------CCHHHHHHHHHHHCCCCCCCCCCCCCCCCCCC</td></tr>
<tr><td>7.</td><td>Leupaxin | LPXN</td><td>92</td><td>101</td></tr>
<tr><td colspan="4">Primary and secondary sequence
YSEAQEPKESPPPSKTSAAAQLDELMAHLTEMQAKVAVRADAGKKHLPDK
CCCCCCCCCCCCCCCCCCCHHHHHHHHHHHHHHHHHCCCCCCCCCCCCCC</td></tr>
<tr><td>8.</td><td>Leupaxin | LPXN</td><td>127</td><td>136</td></tr>
<tr><td colspan="4">Primary and secondary sequence
VAVRADAGKKHLPDKQDHKASLDSMLGGLEQELQDLGIATVPKGHCASCQ
HCCCCCCCCCCCCCCCCCCCCCCCCCHHCCCHHHHHHCCCCCCCCCCCCC</td></tr>
<tr><td>9.</td><td>Paxillin-B | paxB</td><td>10</td><td>19</td></tr>
<tr><td colspan="4">Primary and secondary sequence
-----------MATKGLNMDDLDLLLADLGRPKSSIKVTATVQTTATPSS
-----------CCCCCCCHHHHHHHHHCCCCCCCCCCCCCCCCCCCCCCC</td></tr>
<tr><td>10.</td><td>Paxillin-B | paxB</td><td>108</td><td>117</td></tr>
<tr><td colspan="4">Primary and secondary sequence
VSSQPAPQPPQQSQQIDGLDDLDELMESLNTSISTALKAVPTTPEEHITH
CCCCCCCCCCCCCCCCCCCCHHHHHHHHHHCCCCCCCCCCCCCCCCCCCC</td></tr>
<tr><td>11.</td><td>Paxillin-B | paxB</td><td>231</td><td>240</td></tr>
<tr><td colspan="4">Primary and secondary sequence
SQSQPQPYKVTATNSQPSSDDLDELLKGLSPSTTTTTTVPPPVQRDQHQH
CCCCCCCCCCCCCCCCCCCCHHHHHHCCCCCCCCCCCCCCCCCCCCCCCC</td></tr>
<tr><td>12.</td><td>Paxillin-B | paxB</td><td>310</td><td>319</td></tr>
<tr><td colspan="4">Primary and secondary sequence
NTPNNNNNNNTNSPKVVHGDDLDNLLNNLTSQVKDIDSTGPTSRGTCGGC
CCCCCCCCCCCCCCCCCCCCHHHHHHHHHHHHHCCCCCCCCCCCCCCCCC</td></tr>
<tr><td>13.</td><td>Transforming growth factor beta-1-induced transcript 1 protein | TGFB1I1</td><td>3</td><td>12</td></tr>
<tr><td colspan="4">Primary and secondary sequence
------------------MEDLDALLSDLETTTSHMPRSGAPKERPAEPL
------------------CCHHHHHHHHCCCCCCCCCCCCCCCCCCCCCC</td></tr>
<tr><td>14.</td><td>Transforming growth factor beta-1-induced transcript 1 protein | TGFB1I1</td><td>92</td><td>101</td></tr>
<tr><td colspan="4">Primary and secondary sequence
AAPAAPPFSSSSGVLGTGLCELDRLLQELNATQFNITDEIMSQFPSSKVA
CCCCCCCCCCCCCCCCCCCHHHHHHHHHHHHHCCCCCCCCCCCCCCCCCC</td></tr>
<tr><td>15.</td><td>Transforming growth factor beta-1-induced transcript 1 protein | TGFB1I1</td><td>157</td><td>166</td></tr>
<tr><td colspan="4">Primary and secondary sequence
SLPSSPSPGLPKASATSATLELDRLMASLSDFRVQNHLPASGPTQPPVVS
CCCCCCCCCCCCCCCCCCCCHHHHHHHCCCCHHHCCCCCCCCCCCCCCCC</td></tr>
<tr><td>16.</td><td>Transforming growth factor beta-1-induced transcript 1 protein | TGFB1I1</td><td>203</td><td>212</td></tr>
<tr><td colspan="4">Primary and secondary sequence
PVVSSTNEGSPSPPEPTGKGSLDTMLGLLQSDLSRRGVPTQAKGLCGSCN
CCCCCCCCCCCCCCCCCCCCCCCCHHHHHHHHHCCCCCCCCCCCCCCCCC</td></tr>
<tr><td>17.</td><td>Zinc finger CCCH domain-containing protein 7B | ZC3H7B</td><td>280</td><td>289</td></tr>
<tr><td colspan="4">Primary and secondary sequence
RTLPSTDSLDDFSDGDVFGPELDTLLDSLSLVQGGLSGSGVPSELPQLIP
CCCCCCCCCCCCCCCCCCCCCHHHHHHHCCCCCCCCCCCCCCCCCCCCCC</td></tr>
<tr><td>18.</td><td>Rho GTPase-activating protein 7 | Dlc1</td><td>905</td><td>914</td></tr>
<tr><td colspan="4">Primary and secondary sequence
SILYSSSGELADLENEDIFPELDDILYHVKGMQRIVNQWSEKFSDEGDSD
CCCCCCCCCCCCCCCCCCCCHHHHHHHHHHHHHHHHHHCCCCCCCCCCCC</td></tr>
</table>

Supplementary Table 3.B: Information of the 13 predict LD motifs from round1 predicted by LDMF

Index	Protein name	Start position	End position
1.	Band 4.1-like protein 5 | EPB41L5	634	643
**Primary and secondary sequence**	ETLMLITPADSGSVLKEATDELDALLASLTENLIDHTVAPQVSSTSMITP<br>HHHHCCCCCCCCCCCCCCHHHHHHHHHHHHHHCCCCCCCCCCCCCCCCCC		
2.	Insulin-like growth factor-binding protein 2 | IGFBP2	230	239
**Primary and secondary sequence**	LGLEEPKKLRPPPARTPCQQELDQVLERISTMRLPDERGPLEHLYSLHIP<br>CCCCCCCCCCCCCCCCCHHHHHHHHHHHHHCCCCCCCCCCCCCCCCCCCC		
3.	Protein C8orf37 | C8orf37	4	13
**Primary and secondary sequence**	-----------------MAEDLDELLDEVESKFCTPDLLRRGMVEQPKGC<br>-----------------CHHHHHHHHHHHHHCCCCCCCCCCCCCCCCCCC		
4.	Ral GTPase-activating protein subunit alpha-1 | RALGAPA1	1680	1689
**Primary and secondary sequence**	QFKRFRETVPTWDTIRDEEDVLDELLQYLGVTSPECLQRTGISLNIPAPQ<br>CCCCCCCCCCCCCCCCCCHHHHHHHHHHCCCCCCCCCCCCCCCCCCCCCC		
5.	Uncharacterized protein C16orf71 | C16orf71	267	276
**Primary and secondary sequence**	PLVEPPEGPPVLSLQQLEAWDLDDILQSLAGQEDNQGNRAPGTVWWAADH<br>CCCCCCCCCCCCCCCCCCCCCHHHHHHHHHCCCCCCCCCCCCCCCCCCCC		
6.	Lipoma-preferred partner | LPP	123	132
**Primary and secondary sequence**	GNPGGKTLEERRSSLDAEIDSLTSILADLECSSPYKPRPPQSSTGSTASP<br>CCCCCCCCCCCCCCCCCCHHHHHHHHHHCCCCCCCCCCCCCCCCCCCCCC		
7.	Pre-mRNA 3'-end-processing factor FIP1 | FIP1L1	5	14
**Primary and secondary sequence**	----------------MSAGEVERLVSELSGGTGGDEEEEWLYGGPWDVH<br>----------------CCHHHHHHHHHHHCCCCCCCCCCCCCCCCCCCCC		
8.	Calpastatin | CAST	156	165
**Primary and secondary sequence**	PAVPVESKPDKPSGKSGMDAALDDLIDTLGGPEETEEENTTYTGPEVSDP<br>CCCCCCCCCCCCCCCCCCCHHHHHHHHHCCCCCCCCCCCCCCCCCCCCCC		
9.	Nuclear receptor coactivator 2 | NCOA2	805	814
**Primary and secondary sequence**	KTEKEEMSFEPGDQPGSELDNLEEILDDLQNSQLPQLFPDTRPGAPAGSV<br>CCCCCCCCCCCCCCCCCCHHHHHHHHHHHHCCCCCCCCCCCCCCCCCCCC		
10.	Nuclear receptor coactivator 3 | NCOA3	799	808
**Primary and secondary sequence**	QEKDPKIKTETSEEGSGDLDNLDAILGDLTSSDFYNNSISSNGSHLGTKQ<br>CCCCCCCCCCCCCCCCCCCCCCHHHHHCCCCCCCCCCCCCCCCCCCCCCC		
11.	WASP homolog-associated protein with actin, membranes and microtubules | WHAMM	22	31
**Primary and secondary sequence**	VCESPAERPRDSLESFSCPGSMDEVLASLRHGRAPLRKVEVPAVRPPHAS<br>CCCCCCCCCCCCCCCCCCCCCHHHHHHHHHCCCCCCCCCCCCCCCCCCCC		
12.	Ral GTPase-activating protein subunit alpha-2 | RALGAPA2	1519	1528
**Primary and secondary sequence**	WHRDTFGPQKDSSQVEEGDDVLDKLLENIGHTSPECLLPSQLNLNEPSLT<br>CCCCCCCCCCCCCCCCCCCCCHHHHHHHHHCCCCCCCCCCCCCCCCCCCC		
13.	Purkinje cell protein 2 homolog | PCP2	62	71
**Primary and secondary sequence**	RCSLQAGPGQTTKSQSDPTPEMDSLMDMLASTQGRRMDDQRVTVSSLPGF<br>CCCCCCCCCCCCCCCCCCCCCHHHHHHHHHHHHCCCCCCCCCCCCCCCCC		

Supplementary Table 3.C: Information of the 12 new LD motifs finally suggested by LDMF.

Index	Protein name	Start position	End position
1.	Band 4.1-like protein 5 | EPB41L5	634	643
	**Primary and secondary sequence**<br>ETLMLITPADSGSVLKEATDELDALLASLTENLIDHTVAPQVSSTSMITP<br>HHHHCCCCCCCCCCCCCCHHHHHHHHHHHHHHHCCCCCCCCCCCCCCCCC		
2.	Serine/threonine-protein phosphatase 2A regulatory subunit B'' subunit alpha | PPP2R3A	508	517
	**Primary and secondary sequence**<br>KVSKFEEGDQRDFTNSSSQEEIDKLLMDLESFSQKMETSLREPLAKGKNS<br>CCCCCCCCCCCCCCCCCCHHHHHHHHHHHHHHHHHHHHHCCCCCCCCCCC		
3.	Coiled-coil domain-containing protein 158 | CCDC158	903	912
	**Primary and secondary sequence**<br>ASFLSHHSTKANTLKEDPTRDLKQLLQELRSVINEEPAVSLSKTEEDGRT<br>HHHHCCCCCCCCCCCCCCHHHHHHHHHHHHHHHHCCCCCCCCCCCCCCCC		
4.	Ral GTPase-activating protein subunit alpha-1 | RALGAPA1	1680	1689
	**Primary and secondary sequence**<br>QFKRFRETVPTWDTIRDEEDVLDELLQYLGVTSPECLQRTGISLNIPAPQ<br>CCCCCCCCCCCCCCCCCCHHHHHHHHHHHCCCCCCCCCCCCCCCCCCCCC		
5.	Uncharacterized protein C16orf71 | C16orf71	267	276
	**Primary and secondary sequence**<br>PLVEPPEGPPVLSLQQLEAWDLDDILQSLAGQEDNQGNRAPGTVWWAADH<br>CCCCCCCCCCCCCCCCCCCCCHHHHHHHHHCCCCCCCCCCCCCCCCCCCC		
6.	Lipoma-preferred partner | LPP	123	132
	**Primary and secondary sequence**<br>GNPGGKTLEERRSSLDAEIDSLTSILADLECSSPYKPRPPQSSTGSTASP<br>CCCCCCCCCCCCCCCCCCHHHHHHHHHHCCCCCCCCCCCCCCCCCCCCCC		
7.	Cyclic AMP-responsive element-binding protein 3 | CREB3	49	58
	**Primary and secondary sequence**<br>EAVRAPLDWALPLSEVPSDWEVDDLLCSLLSPPASLNILSSSNPCLVHHD<br>HHHHCCCCCCCCCCCCCCCCCHHHHHHHCCCCCCCCCCCCCCCCCCCCCC		
8.	Calpastatin | CAST	156	165
	**Primary and secondary sequence**<br>PAVPVESKPDKPSGKSGMDAALDDLIDTLGGPEETEEENTTYTGPEVSDP<br>CCCCCCCCCCCCCCCCCCHHHHHHHHHHCCCCCCCCCCCCCCCCCCCCCC		
9.	Nuclear receptor coactivator 2 | NCOA2	805	814
	**Primary and secondary sequence**<br>KTEKEEMSFEPGDQPGSELDNLEEILDDLQNSQLPQLFPDTRPGAPAGSV<br>CCCCCCCCCCCCCCCCCCHHHHHHHHHHHCCCCCCCCCCCCCCCCCCCCC		
10.	Nuclear receptor coactivator 3 | NCOA3	799	808
	**Primary and secondary sequence**<br>QEKDPKIKTETSEEGSGDLDNLDAILGDLTSSDFYNNSISSNGSHLGTKQ<br>CCCCCCCCCCCCCCCCCCCCCHHHHHCCCCCCCCCCCCCCCCCCCCCCCC		
11.	Protein C8orf37 | C8orf37	4	13
	**Primary and secondary sequence**<br>-----------------MAEDLDELLDEVESKFCTPDLLRRGMVEQPKGC<br>-----------------CHHHHHHHHHHHHCCCCCCCCCCCCCCCCCCCC		
12.	Ral GTPase-activating protein subunit alpha-2 | RALGAPA2	1519	1528
	**Primary and secondary sequence**<br>WHRDTFGPQKDSSQVEEGDDVLDKLLENIGHTSPECLLPSQLNLNEPSLT<br>CCCCCCCCCCCCCCCCCCCHHHHHHHHHHCCCCCCCCCCCCCCCCCCCCC		

Supplementary Table 4: Computational Validation

Summary of the bioinformatic search for evidence supportive of interactions between known LD-motif binding proteins and the LDMF-predicted LD motif–containing proteins from the human proteome. We assessed the *LDMF* predictions using four computational methods, namely PrePPI (a Bayesian framework that combines structural, functional, evolutionary and expression information (Zhang, et al., 2012)), GeneFriends (an RNAseq-based gene co-expression network (van Dam, et al., 2015)), CoCiter (which evaluates the significance of literature co-citations (Qiao, et al., 2013)).

To allow straightforward reproducibility, gene names are given in the table. The corresponding protein names for the LDBD-containing proteins are XPO1: exportin; PABPC1: polyadenulate-binding protein 1 (PABP-1); VLC: vinculin; TLN1: talin; PARVA: α-parvin; PARVB: β-parvin; PARVG: γ-parvin; PDCD10: programmed cell death protein 10/cerebral cavernous malformations 3 protein (CCM3); PTK2: focal adhesion kinase (FAK); PTK2B: Protein-tyrosine kinase 2β (PYK2); GIT1: Arf GPTase-activating protein/GRK-interacting protein 1 (GIT1); GIT2: Arf GPTase-activating protein/GRK-interacting protein 2 (GIT2); BCL2: Apoptosis regulator Bcl-2. The corresponding protein names for the predicted LD motif–containing proteins are EPB41L5: Band 4.1-like protein 5 (E41L5); LPP: lipoma-preferred partner (LPP); RALGAPA1: Ral GTPase-activating protein subunit α-1 (RGPA1); PPP2R3a: Serine/threonine-protein phosphatase 2A regulatory subunit B'' subunit α (P2R3A); CCDC158: coiled-coil domain-containing protein 158 (CD158); C16orf71: uncharacterized protein C16orf71 (CP071); NCOA2: nuclear receptor coactivator 2 (NCOA2); NCOA3: nuclear receptor coactivator 3 (NCOA3); CAST: calpastatin (CAST); CREB3: cyclic AMP-responsive element-binding protein 3 (CREB3); RALGAPA2: Ral GTPase-activating protein subunit α-2 (RGPA2); C8orf37: uncharacterized protein C8orf37 (CP037).

Highly likely

gene name	XPO1	PABPC1	VCL	TLN1	PARVA	PARVB	PARVG	PDCD10	PTK2	PTK2B	GIT1	GIT2	BCL2
EPB41L5	0.51	-	0.78	0.99, Small*	0.55	Small*	0.6, Small*	0.67	0.99, Small	1, Small*	-	Small	0.9
LPP	0.98	0.019	Medium, 0.011	Small, 0.015	0.51, Large, 0.01	Small*	Small*	-	Small	Small*, 0.002	0.001	Small	0.047
RALGAPA1	Small	Small*	-	-	-	Small*	-	-	0.76, Small	1	-	Medium	Small
PPP2R3A	-	Small*	Small	-	Small	-	Small*	-	Small	Small*	-	-	-
CCDC158	Small*	-	-	-	-	-	-	-	-	-	Small	-	-
C16orf71	-	-	-	-	-	-	-	-	-	-	Small	-	-

less likely

gene name	XPO1	PABPC1	VCL	TLN1	PARVA	PARVB	PARVG	PDCD10	PTK2	PTK2B	GIT1	GIT2	BCL2
NCOA2	Small	-	0.041	Small	Small*	-	Small	-	-	Small, 0.007	0.046	Large	Small, 0.015
NCOA3	0.98, Small	Small	-	-	Small*	-	-	-	-	Small, 0.016	-	Small	0.77, Small, 0.026
CAST	-	-	Medium, 0.033	Medium	Small	-	-	-	-	Small*, 0.007	0.036	Small	Small, 0.047
CREB3	-	-	Small	Small	Medium	-	Small*	-	0.77	0.87, Small*	Small	Small*	-

least likely

gene name	XPO1	PABPC1	VCL	TLN1	PARVA	PARVB	PARVG	PDCD10	PTK2	PTK2B	GIT1	GIT2	BCL2
RALGAPA2	-	Small	-	Small	Small*	-	Small	-	-	0.93, Small	-	Medium	Small
C8orf37	Small	-	-	Small*	-	-	-	Small	Small	-	-	-	-

Blue is probability score>0.5 from PrePPI tool.

Red is the pearson correlation score from GeneFriends tool between 2 associated genes based on the idea of co-expression.
Small is in a range [0.1,0.3). Medium is in a range [0.3,0.5). Large is in a range [0.5,1]. Star(*) means negative correlation.

Green is the p-value<0.05 from CoCiter tool.

Supplementary Table 5: List of protein accession IDs containing an LD motif

Index	Protein name	Uni-prot accession ID	Organism
1	PAXI_HUMAN	P49023	Homo sapiens
2	LPXN_HUMAN	O60711	Homo sapiens
3	PAXB_DICDI	Q8MML5	Dictyostelium discoideum (Slime mold)
4	TGFI1_HUMAN	O43294	Homo sapiens
5	Z3H7B_HUMAN	Q9UGR2	Homo sapiens
6	RHG07_HUMAN	Q96QB1	Homo sapiens
7	E41L5_HUMAN	Q9HCM4	Homo sapiens
8	P2R3A_HUMAN	Q06190	Homo sapiens
9	CD158_HUMAN	Q5M9N0	Homo sapiens
10	RGPA1_HUMAN	Q6GYQ0	Homo sapiens
11	CP071_HUMAN	Q8IYS4	Homo sapiens
12	LPP_HUMAN	Q93052	Homo sapiens
13	CREB3_HUMAN	O43889	Homo sapiens
14	ICAL_HUMAN	P20810	Homo sapiens
15	NCOA2_HUMAN	Q15596	Homo sapiens
16	NCOA3_HUMAN	Q9Y6Q9	Homo sapiens
17	CH037_HUMAN	Q96NL8	Homo sapiens
18	RGPA2_HUMAN	Q2PPJ7	Homo sapiens
19	CAOG_06505	A0A0D2WU78	Capsaspora owczarzaki
20	H696_03850	A0A058Z589	Fonticula alba
21	AMSG_03633	A0A0L0D4P1	Thecamonas trahens
22	AMAG_12229	A0A0L0SXV7	Allomyces macrogynus
23	AMAG_05806	A0A0L0SDD3	Allomyces macrogynus
24	PTSG_08126	F2UI26	Salpingoeca rosetta
25	SPPG_03527	A0A0L0HLP9	Spizellomyces punctatus
26	AMSG_09408	A0A0L0DLI7	Thecamonas trahens
27	AMSG_06733	A0A0L0DF30	Thecamonas trahens
28	AMAG_15572	A0A0L0T9T0	Allomyces macrogynus
29	AMAG_16615	A0A0L0TBM7	Allomyces macrogynus
30	AMSG_00750	A0A0L0DE48	Thecamonas trahens
31	CAOG_05072	A0A0D2X3J7	Capsaspora owczarzaki
32	H696_03440	A0A058Z6W5	Fonticula alba
33	CAOG_08445	A0A0D2WJ75	Capsaspora owczarzaki
34	CAOG_09259	A0A0D2TZK1	Capsaspora owczarzaki
35	H696_03144	A0A058ZA31	Fonticula alba
36	H696_05642	A0A058Z0Y1	Fonticula alba
37	MONBRDRAFT_12816	A9VDE8	Monosiga brevicollis
38	MONBRDRAFT_24303	A9UW06	Monosiga brevicollis
39	MONBRDRAFT_25678	A9V040	Monosiga brevicollis
40	MONBRDRAFT_37245	A9V0I7	Monosiga brevicollis
41	AMAG_19344	A0A0L0SUQ3	Allomyces macrogynus
42	AMAG_13293	A0A0L0T008	Allomyces macrogynus
43	AMAG_16850	A0A0L0TC97	Allomyces macrogynus
44	SPPG_06473	A0A0L0HB27	Spizellomyces punctatus
45	CAOG_00172	A0A0D2U023	Capsaspora owczarzaki
46	MONBRDRAFT_31043	A9UQW5	Monosiga brevicollis
47	AMAG_01834	A0A0L0S0B6	Allomyces macrogynus
48	AMAG_03174	A0A0L0S4M5	Allomyces macrogynus
49	AMAG_03261	A0A0L0S578	Allomyces macrogynus

50	AMAG_04337	A0A0L0S8Q4	Allomyces macrogynus
51	MVEG_05104	KFH68286	Mortierella verticillata
52	MVEG_11218	A0A086TMK6	Mortierella verticillata
53	AMSG_00923	A0A0L0DIS1	Thecamonas trahens
54	H696_01588T0	A0A058ZE07	Fonticula alba
55	H696_01588T1	A0A058ZFD2	Fonticula alba
56	STK4L_DICDI	Q55FS2	Dictyostelium discoideum
57	AMSG_12414	A0A0L0DSV4	Thecamonas trahens
58	H696_00097	A0A058ZGA4	Fonticula alba
59	AMAG_19087	A0A0L0SN30	Allomyces macrogynus
60	AMAG_12080	A0A0L0SYP4	Allomyces macrogynus
61	AMAG_07384	A0A0L0SI62	Allomyces macrogynus
62	AMAG_20576	A0A0L0TDJ3	Allomyces macrogynus
63	AMAG_12596	A0A0L0SZN5	Allomyces macrogynus
64	AMAG_18842	A0A0L0SIJ4	Allomyces macrogynus
65	AMAG_03098	A0A0L0S4M3	Allomyces macrogynus
66	AMAG_15148	A0A0L0T5Z2	Allomyces macrogynus
67	CAOG_02903	A0A0D2U9S0	Capsaspora owczarzaki
68	PTSG_03224	F2U4K6	Salpingoeca rosetta
69	PTSG_03247	F2U4M7	Salpingoeca rosetta
70	PTSG_04113	F2U6M3	Salpingoeca rosetta
71	PTSG_05218	F2UAU8	Salpingoeca rosetta
72	PTSG_08076	F2UHX6	Salpingoeca rosetta
73	PTSG_10623	F2URW3	Salpingoeca rosetta
74	SARC_01803	A0A0L0GAM7	Sphaeroforma arctica
75	SPPG_01555	A0A0L0HRY4	Spizellomyces punctatus
76	SPPG_01829	A0A0L0HMU1	Spizellomyces punctatus
77	SPPG_02883	A0A0L0HMU5	Spizellomyces punctatus
78	AMSG_00544	A0A0L0D8R0	Thecamonas trahens
79	AMSG_01297	A0A0L0DMR4	Thecamonas trahens
80	AMSG_04901	A0A0L0D889	Thecamonas trahens
81	AMSG_05859	A0A0L0DD16	Thecamonas trahens
82	H696_04460	A0A058Z470	Fonticula alba
82	AMAG_06527	A0A0L0SH78	Allomyces macrogynus

References

Alam, T., *et al.* How to find a leucine in a haystack? Structure, ligand recognition and regulation of leucine-aspartic acid (LD) motifs. *The Biochemical journal* 2014;460(3):317-329.

Altschul, S.F., *et al.* Gapped BLAST and PSI-BLAST: a new generation of protein database search programs. *Nucleic acids research* 1997;25(17):3389-3402.

Arnold, K., *et al.* The SWISS-MODEL workspace: a web-based environment for protein structure homology modelling. *Bioinformatics* 2006;22(2):195-201.

Arold, S.T., Hoellerer, M.K. and Noble, M.E. The structural basis of localization and signaling by the focal adhesion targeting domain. *Structure* 2002;10(3):319-327.

Astro, V., *et al.* Liprin-alpha1 regulates breast cancer cell invasion by affecting cell motility, invadopodia and extracellular matrix degradation. *Oncogene* 2011;30(15):1841-1849.

Backer, J.M., *et al.* Phosphatidylinositol 3'-kinase is activated by association with IRS-1 during insulin stimulation. *The EMBO journal* 1992;11(9):3469-3479.

Bajic, V.B., *et al.* Computer model for recognition of functional transcription start sites in RNA polymerase II promoters of vertebrates. *Journal of molecular graphics & modelling* 2003;21(5):323-332.

Brown, M.C., Curtis, M.S. and Turner, C.E. Paxillin LD motifs may define a new family of protein recognition domains. *Nat Struct Biol* 1998;5(8):677-678.

Carragher, N.O., *et al.* A novel role for FAK as a protease-targeting adaptor protein: regulation by p42 ERK and Src. *Curr Biol* 2003;13(16):1442-1450.

Cooray, P., *et al.* Focal adhesion kinase (pp125FAK) cleavage and regulation by calpain. *The Biochemical journal* 1996;318 (Pt 1):41-47.

Cortes, C. and Vapnik, V. Support-vector networks. *Machine learning* 1995;20(3):273-297.

Durkin, M.E., *et al.* DLC-1:a Rho GTPase-activating protein and tumour suppressor. *Journal of cellular and molecular medicine* 2007;11(5):1185-1207.

Fauchere, J.L., *et al.* Amino acid side chain parameters for correlation studies in biology and pharmacology. *International journal of peptide and protein research* 1988;32(4):269-278.

Gorenne, I., *et al.* LPP expression during in vitro smooth muscle differentiation and stent-induced vascular injury. *Circ Res* 2006;98(3):378-385.

Gorenne, I., *et al.* LPP, a LIM protein highly expressed in smooth muscle. *American journal of physiology. Cell physiology* 2003;285(3):C674-685.

Grant, C.E., Bailey, T.L. and Noble, W.S. FIMO: scanning for occurrences of a given motif. *Bioinformatics* 2011;27(7):1017-1018.

Heon, E., *et al.* Mutations in C8ORF37 cause Bardet Biedl syndrome (BBS21). *Hum Mol Genet* 2016;25(11):2283-2294.

Hoellerer, M.K., *et al.* Molecular recognition of paxillin LD motifs by the focal adhesion targeting domain. *Structure* 2003;11(10):1207-1217.

Ishida, T. and Kinoshita, K. PrDOS: prediction of disordered protein regions from amino acid sequence. *Nucleic acids research* 2007;35(Web Server issue):W460-464.

Ito, A., *et al.* A truncated isoform of the PP2A B56 subunit promotes cell motility through paxillin phosphorylation. *The EMBO journal* 2000;19(4):562-571.

Kall, L., Krogh, A. and Sonnhammer, E.L. Advantages of combined transmembrane topology and signal peptide prediction--the Phobius web server. *Nucleic acids research* 2007;35(Web Server issue):W429-432.

Kallberg, M., *et al.* RaptorX server: a resource for template-based protein structure modeling. *Methods in molecular biology* 2014;1137:17-27.

Kallberg, M., *et al.* Template-based protein structure modeling using the RaptorX web server. *Nature protocols* 2012;7(8):1511-1522.

Kawada, M., *et al.* Cytostatin, an inhibitor of cell adhesion to extracellular matrix, selectively inhibits protein phosphatase 2A. *Biochimica et biophysica acta* 1999;1452(2):209-217.

Kawashima, S., *et al.* AAindex: amino acid index database, progress report 2008. *Nucleic Acids Res* 2008;36(Database issue):D202-205.

Krystkowiak, I. and Davey, N.E. SLiMSearch: a framework for proteome-wide discovery and annotation of functional modules in intrinsically disordered regions. *Nucleic acids research* 2017;45(W1):W464-W469.

Krystkowiak, I., Manguy, J. and Davey, N.E. PSSMSearch: a server for modeling, visualization, proteome-wide discovery and annotation of protein motif specificity determinants. *Nucleic acids research* 2018;46(W1):W235-W241.
la Cour, T*., et al.* Analysis and prediction of leucine-rich nuclear export signals. *Protein Eng Des Sel* 2004;17(6):527-536.
Lo, S.H. Tensin. *The international journal of biochemistry & cell biology* 2004;36(1):31-34.
Lorenz, S*., et al.* Structural analysis of the interactions between paxillin LD motifs and alpha-parvin. *Structure* 2008;16(10):1521-1531.
McGuffin, L.J., Bryson, K. and Jones, D.T. The PSIPRED protein structure prediction server. *Bioinformatics (Oxford, England)* 2000;16(4):404-405.
Moldoveanu, T., Gehring, K. and Green, D.R. Concerted multi-pronged attack by calpastatin to occlude the catalytic cleft of heterodimeric calpains. *Nature* 2008;456(7220):404-408.
Moscardo, A*., et al.* Serine/threonine phosphatases regulate platelet alphaIIbbeta3 integrin receptor outside-in signaling mechanisms and clot retraction. *Life Sci* 2013;93(20):707-713.
Nag, S*., et al.* Ca2+ binding by domain 2 plays a critical role in the activation and stabilization of gelsolin. *Proc Natl Acad Sci U S A* 2009;106(33):13713-13718.
Petit, M.M*., et al.* LPP, an actin cytoskeleton protein related to zyxin, harbors a nuclear export signal and transcriptional activation capacity. *Mol Biol Cell* 2000;11(1):117-129.
Petit, M.M., Meulemans, S.M. and Van de Ven, W.J. The focal adhesion and nuclear targeting capacity of the LIM-containing lipoma-preferred partner (LPP) protein. *The Journal of biological chemistry* 2003;278(4):2157-2168.
Qiao, N*., et al.* CoCiter: an efficient tool to infer gene function by assessing the significance of literature co-citation. *PloS one* 2013;8(9):e74074.
Quan, J. and Tian, J. Circular polymerase extension cloning of complex gene libraries and pathways. *PloS one* 2009;4(7):e6441.
Ravi, A*., et al.* Epidermal growth factor activates the Rho GTPase-activating protein (GAP) Deleted in Liver Cancer 1 via focal adhesion kinase and protein phosphatase 2A. *The Journal of biological chemistry* 2015;290(7):4149-4162.
Schell, C*., et al.* The FERM protein EPB41L5 regulates actomyosin contractility and focal adhesion formation to maintain the kidney filtration barrier. *Proceedings of the National Academy of Sciences of the United States of America* 2017;114(23):E4621-E4630.
Schmalzigaug, R*., et al.* GIT1 utilizes a focal adhesion targeting-homology domain to bind paxillin. *Cellular signalling* 2007;19(8):1733-1744.
Schwede, T*., et al.* SWISS-MODEL: An automated protein homology-modeling server. *Nucleic Acids Res* 2003;31(13):3381-3385.
Shawe-Taylor, J. and Cristianini, N. Kernel methods for pattern analysis. Cambridge university press; 2004.
Shirakawa, R*., et al.* Tuberous Sclerosis Tumor Suppressor Complex-like Complexes Act as GTPase-activating Proteins for Ral GTPases. *Journal of Biological Chemistry* 2009;284(32):21580-21588.
Stashi, E., York, B. and O'Malley, B.W. Steroid receptor coactivators: servants and masters for control of systems metabolism. *Trends Endocrinol Metab* 2014;25(7):337-347.
Stothard, P. The sequence manipulation suite: JavaScript programs for analyzing and formatting protein and DNA sequences. *BioTechniques* 2000;28(6):1102, 1104.
Tumbarello, D.A., Brown, M.C. and Turner, C.E. The paxillin LD motifs. *FEBS letters* 2002;513(1):114-118.
UniProt, C. UniProt: a hub for protein information. *Nucleic acids research* 2015;43(Database issue):D204-212.
van Dam, S., Craig, T. and de Magalhaes, J.P. GeneFriends: a human RNA-seq-based gene and transcript co-expression database. *Nucleic acids research* 2015;43(Database issue):D1124-1132.
van Zundert, G.C.P*., et al.* The HADDOCK2.2 Web Server: User-Friendly Integrative Modeling of Biomolecular Complexes. *Journal of molecular biology* 2016;428(4):720-725.
Vitour, D*., et al.* RoXaN, a novel cellular protein containing TPR, LD, and zinc finger motifs, forms a ternary complex with eukaryotic initiation factor 4G and rotavirus NSP3. *J Virol* 2004;78(8):3851-3862.
Wang, G. and Dunbrack, R.L., Jr. PISCES: a protein sequence culling server. *Bioinformatics (Oxford, England)* 2003;19(12):1589-1591.

Williamson, M.P. Using chemical shift perturbation to characterise ligand binding. *Prog Nucl Mag Res Sp* 2013;73:1-16.
Zacharchenko, T.*, et al.* LD Motif Recognition by Talin: Structure of the Talin-DLC1 Complex. *Structure* 2016;24(7):1130-1141.
Zhang, Q.C.*, et al.* Structure-based prediction of protein-protein interactions on a genome-wide scale. *Nature* 2012;490(7421):556-560.
